# Theoretical exploration of the reactivity of cellulose models under non‐thermal plasma conditions—mechanistic and NBO studies

**DOI:** 10.1002/jcc.26934

**Published:** 2022-06-07

**Authors:** Walid Lamine, Frédéric Guégan, François Jérôme, Gilles Frapper

**Affiliations:** ^1^ IC2MP UMR 7285 Université de Poitiers – CNRS Poitiers; ^2^ Université de Pau et des Pays de l'Adour, E2S UPPA, CNRS, IPREM, UMR 5254 Pau cedex 09 France

## Abstract

Mechanistic details of cellulose depolymerization by non‐thermal (atmospheric) plasma (NTAP) remains under‐explored given the complexity of the medium. In this study, we have investigated the reaction mechanism of glycosidic‐bond degradation triggered by reaction with hydroxyl radicals, considered as the principal reactive species in NTAP medium. In the first step of reaction sequence, H‐abstraction reactions by HO^‧^. radical on different C—H sites of the pyranose ring were found to be non‐selective and markedly exergonic giving rise to a set of cellobiosyl carboradicals likely to undergo further reactions. We then showed that cellobiosyl carboradicals are protected against direct hydrolysis, no activation of the (1–4)‐ β‐glycosidic bond being characterized. Interestingly, a simple homolytic bond cleavage allowed to obtain desired monomer. Among the 18 possible fragmentations, involving C—C and C—O bond breaking from cellobiosyl carboradicals, 14 transition states were successfully identified, and only three reaction pathways proved kinetically and thermodynamically feasible. Natural bond orbital (NBO) analysis was performed to shed light on electronic structures of different compounds.

## 1 INTRODUCTION

Cellulose, the most abundant linear poly (1–4)‐ β ‐D‐glucan organic polymer in the biosphere, has gained in the last decades a considerable attention as a huge reservoir of renewable carbon reducing the consumption of fossil‐fuel based materials.^1^, ^2^ In fact, cellulose can be depolymerized to glucose which is an important platform molecule for the production of valuable chemicals such as ethylene glycol, hexitol, levulinic acid, 5‐hydroxy‐methylfurfural, ethanol, liquid alkanes,^3^, ^4^, ^5^ among many others. However, despite the pivotal role cellulose can play in bioeconomy, and the great interest for its use to produce fuel and advanced materials, depolymerization of this biopolymer remains a critical challenge due to its high crystallinity, making it recalcitrant to hydrolysis at ambient conditions. Accordingly, many efforts have been given to the development of pretreatment methods allowing partial hydrolysis of resistant cellulose (ball‐milling, dissolution of cellulose in ionic liquids).^6^, ^7^, ^8^ Pioneer investigations reported by some of the present authors showed a significant enhancement of hydrolysis to valuable glucans could be obtained by non‐thermal atmospheric plasma (NTAP) treatment.^3^, ^9^, ^10^, ^11^, ^12^ Moreover, NTAP methodology allows to avoid laborious purification procedures, as no catalyst or solvent is necessary.

On the other hand, given the complex cocktail of excited species, and particularly radicals generated during NTAP treatment, exact mechanism governing depolymerization of cellulose is difficult to elucidate. Based on the previous studies,^13^, ^14^, ^15^ hydroxyl radical was proposed to be the principal reactive species created in NTAP medium, promoting the partial cleavage of the β‐1,4 glycosidic bond through the formation of carboradicals on the glucosyl units. F. Jérôme et al.^9^ have shown that under air, the water trapped in the cellulosic backbone plays a very important role; according to its content, two competitive reactions (depolymerization/re‐polymerization) are possible. For the highest water content, the depolymerization process is dominant, inducing the cleavage of the glycosidic bond by propagation reaction from the surface to the bulk via hydroxyl radicals generated by water and radicals formed in the reaction. However, a limited oxidation at the cellulose surface has been observed. In the case of a decreased amount of water, soluble branched glucans were formed by the assembling of the short chain cyclodextrins.

It must be noted that in any case the nature of the carrier gas appears to have a limited impact, as depolymerization is observed either under air or pure gases (oxygen, nitrogen), unlike what was observed in the case of inulin at an NTP/water interface (in this last case, depolymerization is presumed to be activated by in‐situ generated acids).^12^

**SCHEME 1:**
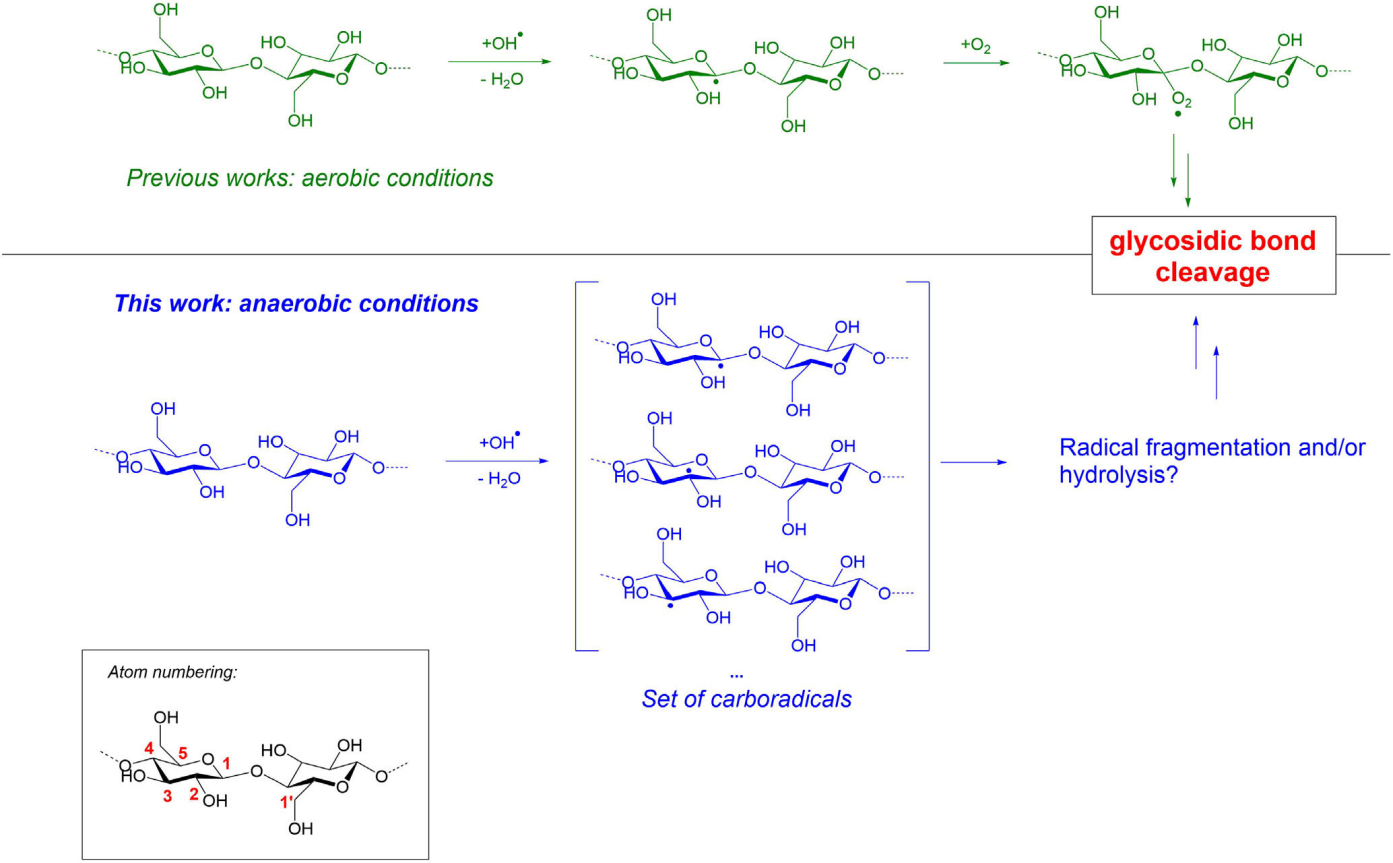
Proposed and studied reaction mechanisms for the NTAP‐induced glycosidic bond cleavage. Inset: Carbon atom numbering within the cellobiose moiety.

To the best of our knowledge, only three mechanistic pathways have been proposed for glycosidic bond cleavage in the presence of hydroxyl radicals, two of them involving an electron transfer between the saccharide radical and O_2_ gas (see Scheme 1).^16^, ^17^ Given that in our case—NT(A)P—depolymerization also occurs in the absence of O_2_, our aim is to propose a plausible mechanism independently of the pulsing gas. A recent study proposed different energetic profiles of cellulose degradation to small molecular products (oxaldehyde, malonaldehyde, and 2‐hydroxysuccinaldehyde) under the action of a large excess of hydroxyl radicals.^18^ In that case, the presence of such a large excess of radicals explains the observed degradation reactions (oxidations), which are not observed in the case of NTAP treatment. It must additionally be noted that in the former work only a subset of all possible reactions were considered owing to the chosen methodology (reactive force‐field dynamics).

Herein, we examine the reactivity of a model compound, cellobiose, against hydroxyl radicals and water molecules. In the first stage, we focused on the abstraction of hydrogen atoms by hydroxyl radicals on the various C—H bonds of the pyranose rings. Noteworthily, no selectivity is expected from this first step, all activation barriers being found in a narrow energy range. Then, in the second step, we evaluated the reactivity of the resulting carboradicals towards hydrolysis and fragmentation. Interestingly, no activation of the glycosidic bond toward hydrolysis can be evidenced, but its cleavage through fragmentation indeed appears possible and rather selective, alternative fragmentation reactions being kinetically and thermodynamically disfavored. It may be noted that the proposed mechanism strongly differs from that proposed in the case of the acid‐catalyzed hydrolysis.^19^, ^20^

## 2 COMPUTATIONAL METHODS

All calculations were performed at the uB3LYP/6–31++G(d,p) level of theory,^21^, ^22^ as implemented in Gaussian 09 rev B.01.^23^ Cellobiose starting structure was extracted from the crystal structure of the main polymorph of cellulose,^24^ and the dangling bonds saturated by hydroxyl groups. Its molecular structure and atom numbering are given in Scheme 1. All geometries were then fully relaxed, and nature of the found stationary points was confirmed by frequencies calculations. Intrinsic reaction coordinate (IRC) calculations were also performed to confirm the found transition states indeed connect the expected reagents and products. Thermodynamic corrections (*T* = 298 K, 1 atm) were considered for the construction of the reaction paths. When relevant, spin‐density maps were computed using Cubegen, and displayed using GaussView 5. All radicals were considered in a doublet spin state, and spin contamination was checked to ensure the correct state was reached. NBO analysis^25^ (NBO3.1 for Gaussian) was used to get a further insight on electronic structures of the various compounds under study. Additional calculations conducted at the wB97xD/6–311++G(2d,p) level showed comparable results to those found using B3LYP (see Tables S3–S4 in Section S5 in ESI for the details). Since these results were obtained at the expense of a significantly larger computational demand (convergences being quite complicated with this level of theory), B3LYP/6–31++G(d,p) was preferred for the rest of the study.

**SCHEME 2:**
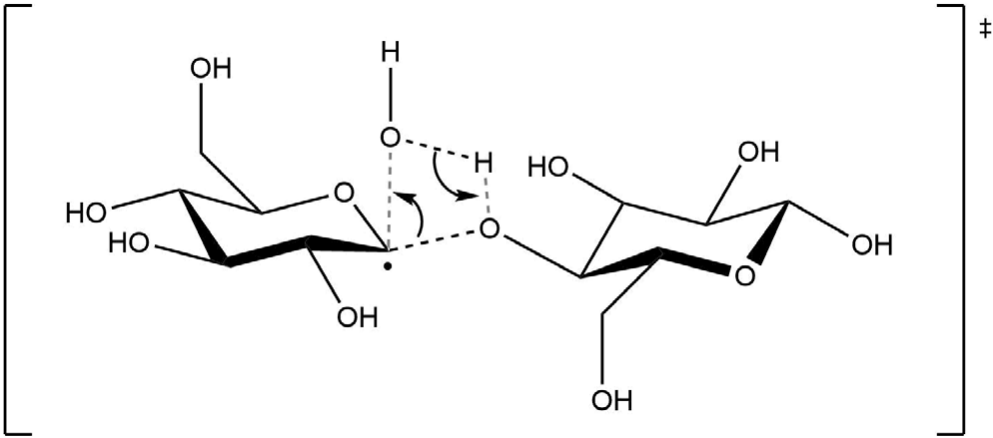
Schematic representation of the 4‐centers, concerted transition state of hydrolysis for radical **P1**.

## 3 RESULTS AND DISCUSSION

### 3.1 Hydrogen abstraction

As indicated in the previous experimental works, the depolymerization reaction is assumed to begin by the reaction of cellulose with an active species, likely a hydroxyl radical HO^‧^, leading to the abstraction of a H atom from a C—H bond on the pyranose ring. Such H‐abstractions have indeed been evidenced experimentally, by the ESR characterization of cellulose samples submitted to an argon‐based non thermal plasma.^15^ Furthermore, cellulose is known to incorporate a significant amount of water (e.g., microcrystalline cellulose Avicel PH 105 has a water content of 5wt %),^3^ and this water content proved experimentally to have a strong influence on the outcome of the reaction.^9^ When increasing the reaction time, a loss of water was observed in the reaction medium, which was accompanied by the observation of repolymerization products. These two observations thus suggested that water, and likely hydroxyl radicals, play a significant role in the propagation step of the mechanism—thus supporting the choice of HO^‧^ as model active species.

**FIGURE 1:**
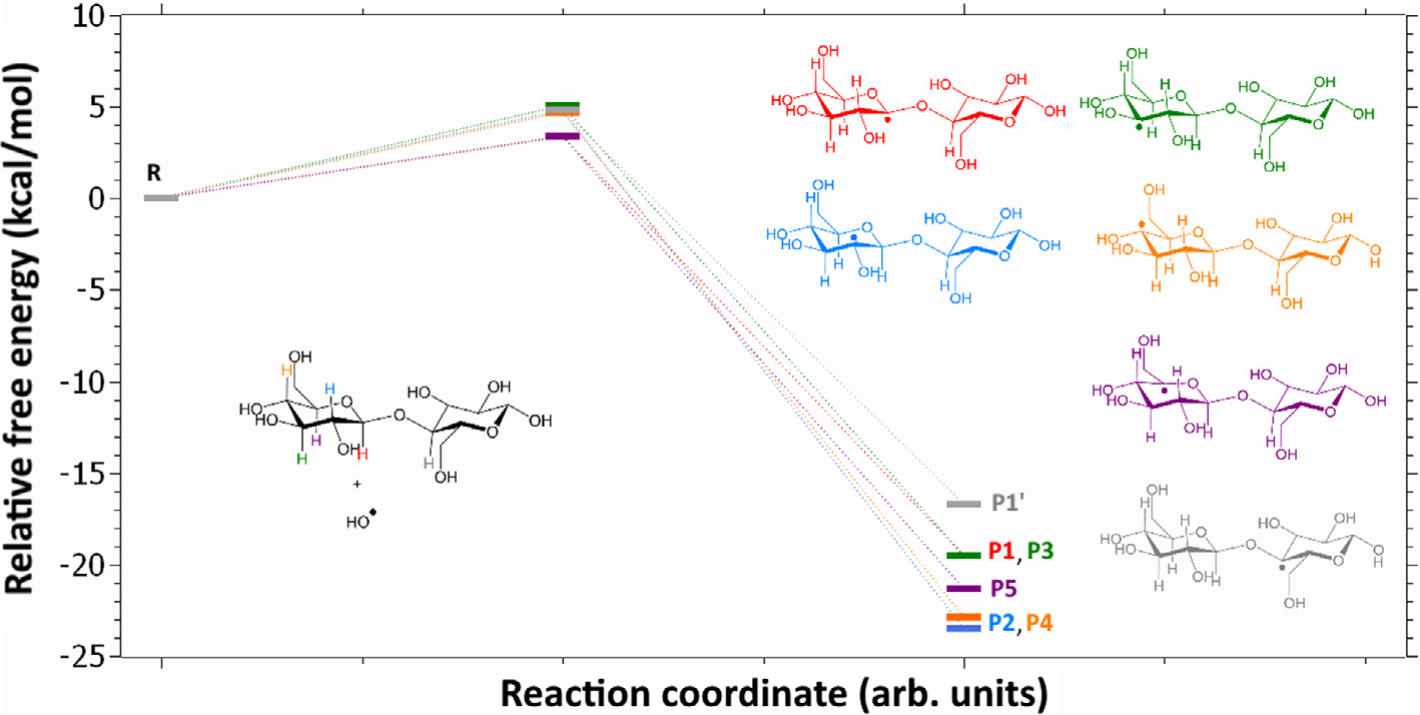
Free energy profiles for all considered H‐abstraction reaction. Reagents (cellobiose and HO^‧^ radical at infinity, denoted **R**) and resulting carboradical structures are depicted and colored according to the following scheme: Red, H abstraction at C1 position; blue, at C2; green, at C3; orange, at C4; purple, at C5; gray, and at C1' (H_2_O co‐product not shown)

The energy profiles associated to all possible H‐abstraction by HO^‧^ on the cellobiose ring positions are given in Figure 1. Several features are noteworthy. First, all H‐abstraction appear feasible in the reaction conditions, both from kinetics and thermodynamics point of view. Activation barriers are indeed rather moderate, the maximal free energy of activation being ca. 5.1 kcal/mol with respect to the separated reagents, whereas free energies of reaction are markedly negative (ranging from −16.7 kcal/mol to −23.5 kcal/mol).

Both facts could actually be expected: experiments showed the cellobiose‐derived carboradicals may be long lived, suggesting they are rather stable. Conversely, HO^‧^ is a quite unstable thus reactive compound. Abstraction products are thus comparatively more stable than reagents, hence accounting for the marked exergonic character of the reaction step. From the Hammond postulate,^26^ early transition states with moderate activation barriers are furthermore expected, as observed. The early character of the transition states additionally reflects in the localization of the spin density on the oxygen atom of HO^‧^, which is in all case still marked in the transition state.

**FIGURE 2:**
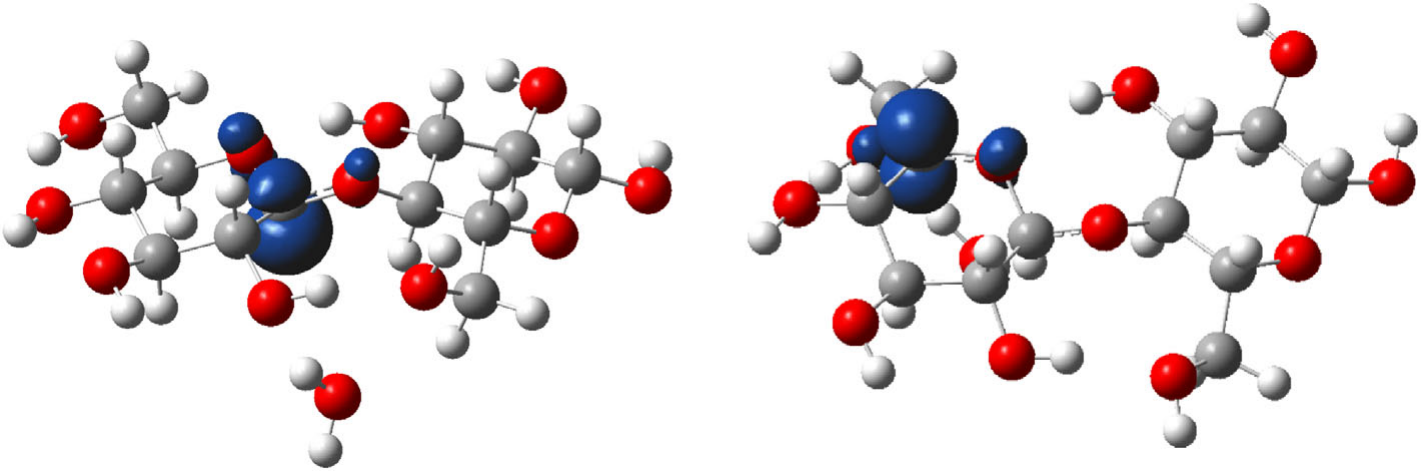
Spin density isosurfaces for products **P1** (left) and **P5** (right), for an isovalue of 0.01 a.u. atoms color scheme: Red, O; gray, C; white, H

But a more interesting and less predictable result is the low kinetic selectivity. Activation free energies are indeed all found in a narrow range, ca. 3.4–5.1 kcal/mol above the isolated reagents. Since these values are rather close and small, and as reaction was not performed at low temperature, one may consider that all H‐abstraction products may form experimentally. Thus, in contrast with the previous related studies, we cannot assume here that H‐abstraction only occurs at the C1 position. A complete set of carboradicals can be formed, and each of these radicals is likely to undergo further reactions. These radicals are all well‐localized on the corresponding C atoms, as revealed by spin‐density maps (see Figure 2 for products **P1** and **P5**). In the case of products **P1**–**P4** and **P1**', the shape of the spin‐density on the C atoms is reminiscent of a s‐p hybridized C atomic orbital, which could be expected from the local pyramidal geometry around the radicalar carbon. Conversely, in the case of product **P5** a planar radical is observed, and the shape of the spin‐density in this case is more reminiscent of pure p atomic orbital.

**FIGURE 3:**
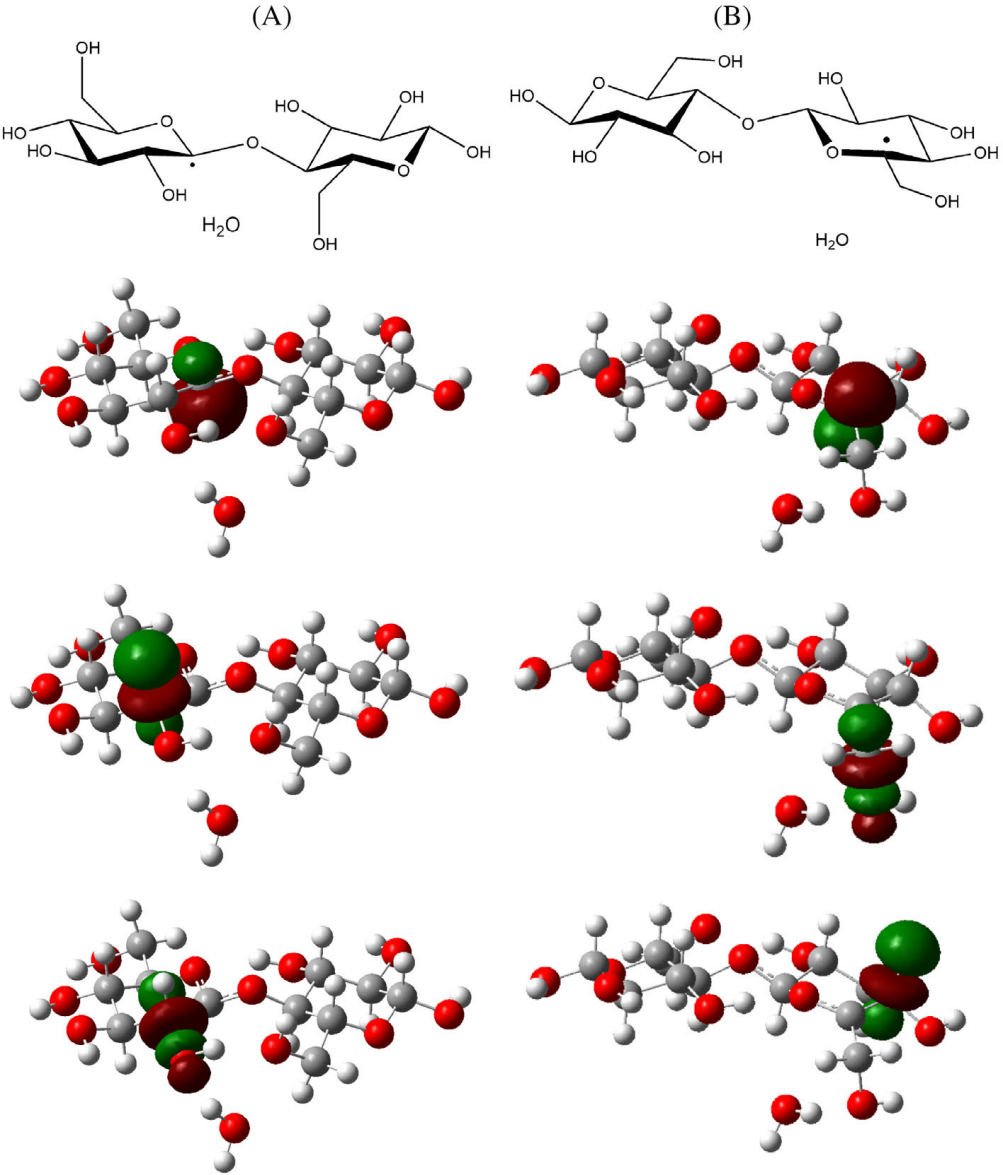
From top to bottom: Schematic representation of the molecular structures and orientation, calculated isosurface corresponding to the lone‐electron NBO, isosurface of the acceptor antibonding NBO associated with the highest second‐order stabilization with the donor lone‐electron NBO, isosurface of the acceptor antibonding NBO associated with the second highest second‐order stabilization with the donor lone‐electron NBO, for: (A), radical product **P1**; (B), radical product **P5**. Isosurfaces were drawn at 0.06 a.u

NBO analysis helps to gain more insight on these observations. For all abstraction product, the spin‐up electrons NBO diagram displays an additional orbital, developing only on the radical carbon. As we show in Figure 3, this orbital presents a significant degree of s‐p mixing for products **P1** to **P4** and **P1**', while it is a pure p atomic orbital in **P5** (see Section S1 in the SI for detailed compositions). Second‐order analysis furthermore suggests the unpaired electron in this orbital is stabilized through hyperconjugation with neighboring antibonding orbitals.^27^ In the case of products **P2** to **P4** and **P1**', the largest stabilization is observed with the σ*(C—H) orbitals in direct vicinity of the radical centers. These orbitals are mostly developed on the H atoms, and according to the second‐order stabilization is rather low (around 4–5 kcal/mol per C—H bond), thus the radical geometry is not perfectly planar. In the case of **P1**, both the C—H and C—O antibonding orbitals involving C2 interact with the lone‐electron orbital at second order, but the stabilization remain rather small (3.8 kcal/mol and 1.8 kcal/mol, respectively) as a consequence of a poor overlap between orbitals. Thus in that case to a certain degree of pyramidalisation is expected on the radical at C1. In the case of product **P5** on the other hand, one of the neighboring σ* orbital corresponds to a C—O bond, which is thus mostly located on the C atom, and it is more ideally oriented to interact with the lone‐electron orbital on the C atom. Stronger overlap is expected and according to the second‐order stabilization is also much larger (ca. 11 kcal/mol).

Thus, the unpaired electron is more efficiently stabilized by hyperconjugation, accounting for the planarity of the associated carbon atom C5.

### 3.2 Hydrolysis

Products of the first reaction step are carboradicals and a water molecule. It is known experimentally that cellulose can be hydrolysed in strongly acidic or basic aqueous solution—such harsh conditions being required to activate the otherwise inert glycosidic bond. It thus seemed relevant here to evaluate whether the H‐abstraction would provide any activation of this bond towards hydrolysis (through concerted addition of O—H over the C—O bond). We thus first focused on carboradicals **P1** and **P1**', since they are formed by abstraction of a H atom in direct vicinity of the glycosidic bond. In both cases, a 4‐centers transition state, corresponding the concerted, quasi‐synchronous addition of H_2_O on the glycosidic bond could be located (see Scheme 2 for a representation of the transition state structure in the case of **P1**).

However, these transition states are found at a very high energy, an activation barrier of 63.1 kcal/mol being for instance obtained in the case of **P1**. Such high activation barriers cannot be overcome in the experimental conditions (room temperature, atmospheric pressure). Aside from the possibly retained chemical inertia of the glycosidic bond, two factors can be put forward to account for the magnitude of the activation barrier: first, a non‐negligible Pauli repulsion can be expected in the transition state, as the electron‐rich O atom from the water molecule approaches the rather well‐localized unpaired electron on the carbon atom. Additionally, it may be surmised that the direct addition of the O—H bond on the glycosidic C—O bond requires a strong geometric distortion of both reagents, which results in a marked destabilization of the associated transition state. Involvement of a second water molecule, through a Grotthuss‐like mechanism,^28^ may here relieve a consequent part of this deformation energy (6‐centres transition state in a cyclohexane‐chair conformation).^29^ Such transition states could indeed be located, and a substantial stabilization of the transition states is evidenced; for instance, in the case of radical **P1** the barrier reduced to 50.6 kcal/mol. This value remains nonetheless too high to allow this reaction to occur in experimental conditions.

Here also, NBO analysis may be used to understand the apparent lack of activation of the glycosidic bond in the case of the radicals, at variance with the case of acidic catalysis for instance.^19^ The marked stability of the glycosidic bond in cellulose is, among other factors, accounted for by the marked hyperconjugation of one of the glycosidic oxygen lone pairs with the C1—O18 antibonding * orbital (*exo*‐anomeric effect).^19^ As shown in Table 1, in the case of cellobiose, this hyperconjugation amounts to ca. 16 kcal/mol. Interestingly, this value remains quite preserved in products **P1** to **P5** (ca. 15–20 kcal/mol by summing contribution from spin‐up and spin‐down orbitals)^i^: C—H abstraction does not activate the glycosidic bond, hence explaining the high activation barriers found here.

**TABLE 1:** Details of the NBO analysis for cellobiose (first line) and radicals **P1** to **P1**'. For each spin, energy of the anti‐bonding $\sigma^{⋆}$ MO associated with the endocyclic C1—O18 bond is given (in a.u.), along with its population, C—O relative atomic contributions (in percent), and second‐order stabilization with donor lone‐pair NBO from glycosidic oxygen O21 (in kcal/mol)

Radical	Spin	E(C1—O18)*	Pop	C—O polarization	2nd order
Cellobiose		0.25049	0.07123	68.31–31.69	16.05
**P1**	α	0.30716	0.03194	69.87–30.13	7.96
	β	0.31206	0.02663	69.87–30.13	4.14, 1.79
**P2**	α	0.25853	0.03495	67.81–32.19	7.50
		0.25828	0.03317	67.69–32.31	7.54
**P3**	α	0.24849	0.03539	68.27–31.73	8.06
	β	0.24880	0.03543	68.27–31.73	8.04
**P4**	α	0.26054	0.03419	68.10–31.90	7.81
	β	0.26061	0.03415	68.08–31.92	7.81
**P5**	α	0.21695	0.04794	68.95–31.05	8.91
	β	0.21792	0.04608	70.36–29.61	8.99
**P1**'	α	0.25397	0.03145	68.17–31.83	6.81
	β	0.25449	0.02986	68.27–31.73	1.91, 3.86*

### 3.3 Fragmentation

**FIGURE 4:**
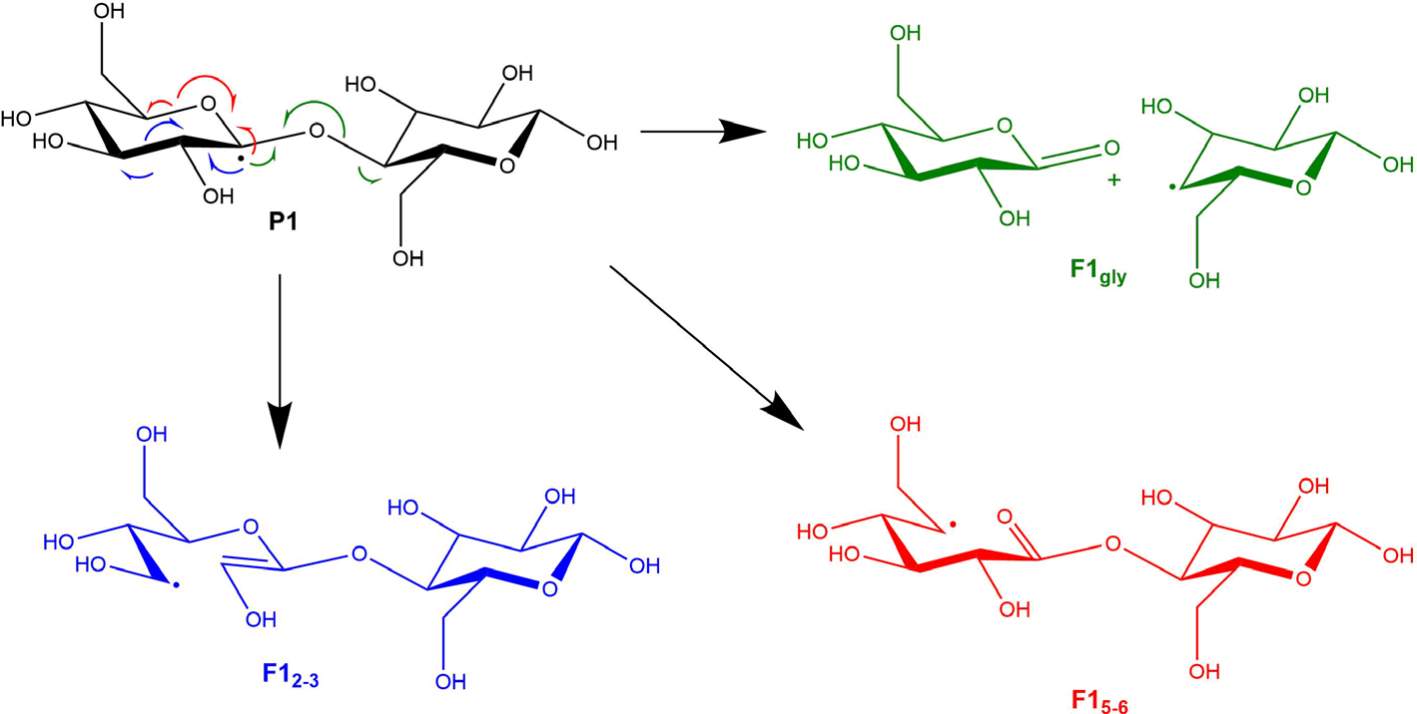
Possible electronic reorganization of radical **P1**

Since hydrolysis seems hardly possible in experimental conditions, we then turned our attention to a possible direct homolytic C—O bond cleavage. From the Lewis structures of products **P1**—**P1**', several electronic rearrangements can be proposed using classical organic chemistry knowledge and curly arrows; see, for instance, Figure 4 for the case of **P1** (and refer to Figures S4–S9 in Section S3 in SI for the complete list). In most cases, these rearrangements lead to the formation of another carboradical, but it must be noted that in the case of products **P2**, **P4**, and **P1**', formation of an oxoradical can also be proposed.

Overall, 18 fragmentation paths can be conceived this way. Interestingly, a transition state could be located for each fragmentation leading to a carboradical, while in the case of oxoradicals only two transition state are found, starting from **P1**' and **P4**. Homolytic fragmentation of C—O bond thus appears more difficult, which could in fact be expected from their stronger polarity, expected to favor heterolytic cleavage.

In the end, 14 reaction paths could be computed—see section S4 and Table 2 for the detailed list. Activation free enthalpies for fragmentations range between 11.7 kcal/mol (glycosidic bond cleavage from radical **P1**) to 31.2 kcal/mol (C3—C4 bond cleavage in radical **P5**), already suggesting some fragmentations may be kinetically disfavored.

**TABLE 2:** Activation and reaction free energies for all computed fragmentation reactions, ordered by radical and cleaved bond (*gly* referring to the glycosidic bond

Radical	Cleaved bond	$\Delta G^{†}$ (kcal/Mol)	$\Delta G^{†}$ (kcal/Mol)
**P1**	*gly*	11.7	−15.1
**P1**	C2—C3	28.8	11.9
**P1**	C5—O6	13.7	−7.6
**P2**	C1—O6	18.1	6.7
**P2**	C3—C4	29.1	20.4
**P3**	C1—C2	24.7	13.4
**P3**	C4—C5	22.0	12.6
**P4**	C2—C3	29.3	18.1
**P4**	C5—O6	19.7	14.9
**P5**	C1—O6	13.4	4.0
**P5**	C3—C4	31.2	10.2
**P1**'	C2'—C3'	23.5	3.1
**P1**'	*gly*	18.8	−16.5
**P1**'	C5'—O6'	21.6	3.9

As indicated before, H‐abstractions on cellobiose are associated with a reaction free energy of approximately −20 kcal/mol. If no energy dissipation were active, this free energy of reaction could be used for fragmentation—hence in the first crude approach we may consider that fragmentations associated with activation barriers below 20 kcal/mol may be realized.

With such a condition, only 6 fragmentations are left: the glycosidic bond cleavage in radicals **P1** ($\Delta G^{†}=11.7$ kcal/mol) and **P1**' ($\Delta G^{†}=18.8$ kcal/mol), the C1—O6 bond cleavage in radicals **P2** ($\Delta G^{†}=18.1$ kcal/mol) and **P5** ($\Delta G^{†}=13.4$ kcal/mol), and the C5—O6 bond cleavage in radicals **P1** ($\Delta G^{†}=13.7$ kcal/mol) and **P4** ($\Delta G^{†}=19.7$ kcal/mol).

Thus some selectivity is already evidenced at this stage, but it is further reinforced by taking thermodynamics into account. Indeed, among the previous fragmentation reactions, only three are associated with a negative free energy of reaction: the glycosidic bond cleavages starting from **P1** (ΔG=−15.1 kcal/mol) and **P1**' (ΔG=−16.5 kcal/mol), and the C5—O6 bond cleavage in **P1** (−7.6 kcal/mol).

Overall, a non‐negligible selectivity may be expected for the glycosidic‐bond cleavage, especially starting from radical **P1**, which is associated to the lowest activation barrier in the whole series of fragmentations, combined with a marked exergonicity.

Now one may wonder whether the associated fragmentation products are the expected ones. The radical resulting from the glycosidic‐bond cleavage in **P1** (in the species called $F1_{\mathrm{gly}}$ in Figure 4) could be trapped by an hydroxyl radical—which are supposed to be produced in rather large quantity in NTAP conditions—forming the desired glucan, here glucose. However in our proposed mechanism the counterpart is not a glucose molecule but an oxidation product (lactone).

Experimentally, very low contents of oxidation products were characterized. At first, this may seem to contradict our proposition. However, it must be reminded that experiments also showed that NTAP treatment itself is not sufficient to observe a significant depolymerization: in order to retrieve glucose, NTAP‐treated cellulose should then be subjected to mild acidic hydrolysis. The proposed explanation was that NTAP leads to the cleavage of a few glycosidic bonds in the solid, forming relatively long oligomers but which are expected to be more reactive towards hydrolysis than the starting cellulose.

**FIGURE 5:**
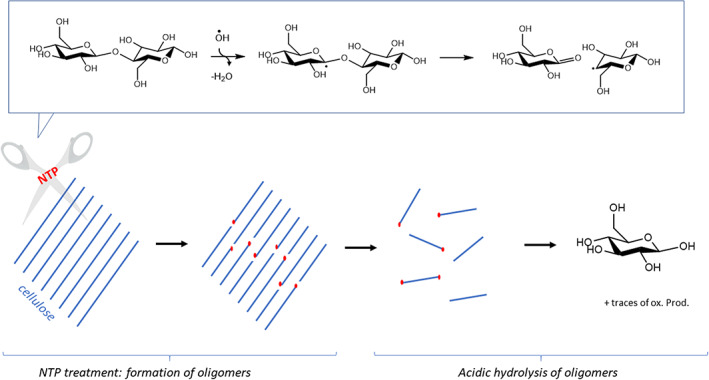
Proposed mechanism for the activation of cellulose hydrolysis by NTP treatment

Under such assumption, our proposition may meet experimental data (see a graphical summary on Figure 5): provided that long enough oligomers are produced, only a limited proportion of oxidation products should be formed. If NTAP treatment leads to the production of oligomers consisting in N glucose rings in average, after hydrolysis only 1 glucose molecule over N will be oxidized. Hence with a large enough N oxidation may be nearly impossible to detect.

## 4 CONCLUSION

In this publication, we modeled a possible reaction mechanism for the NTAP‐induced depolymerization of cellulose, using cellobiose and hydroxyl radicals as model reagents. In line with previous studies on related systems, the first step of the reaction is a H‐atom abstraction from endocyclic C—H bonds. No selectivity is observed in this first step, all C—H abstraction being found in a very narrow activation energy range. Despite the C—H abstraction, the resulting radicals proved to be essentially non‐reactive toward direct hydrolysis of the glycosidic bond. Detailed analysis of the electronic structure of these radicals (via NBO calculations) revealed that hyperconjugation to and from the glycosidic bond is almost left untouched by the C—H abstraction, explaining the apparent lack of activation of this bond toward hydrolysis.

On the other hand, simple homolytic‐bond cleavage, resulting from a reorganization of the radicals electron density, proved to be a feasible process. Interestingly, for each radical a set of reorganizations is in principle possible, but most of them appear to be kinetically and/or thermodynamically disfavored. In the end, three reaction paths appear kinetically feasible, and among these the glycosidic‐bond cleavage from radical **P1** (stemming from the H‐abstraction on carbon C1) is the most favored reaction. Hence a marked selectivity towards glycosidic bond cleavage is retrieved in the second step of this mechanism, in line with experimental observations. It is however important to remember that our model is quite simplified with respect to the actual reagent (cellulose). A further study of the impact of the embedding within a solid‐state matrix, as well as solid‐state interactions (intramolecular H bonds) on the reaction profiles, seems required in order to confirm the validity of the proposed mechanism. This will be the topic of a forthcoming publication.

## SUPPORTING INFORMATION

Detailed NBO analyses, reaction profiles, and geometries in Cartesian coordinates format.

## Supporting information


**APPENDIX S1** Supporting Information

## Data Availability

Basic data (optimised geometries and energies) are provided in supplementary material. Detailed original data are available on request from the authors.

## Endnote

^i^
In the case of **P1**, both lone pairs of O_21_ contribute to the hyperconjugation in the β spin system, while in the case of **P1’** the second lone pair is replaced by a strongly distorted C—O π bond (OC polarization: 90.46–9.54)
