# Supplementary material for: Theoretical exploration of the reactivity of cellulose models under non‐thermal plasma conditions—mechanistic and NBO studies
Source: J Comput Chem. 2022 Jun 7;43(20):1334–41. doi: 10.1002/jcc.26934 (PMC9327522; doi:10.1002/jcc.26934)
Supplement: Supplementary file 1 — APPENDIX S1 Supporting Information [file JCC-43-1334-s001.pdf]

Supporting Information - Theoretical exploration of the reactivity of cellulose models under non-thermal plasma conditions - mechanistic and NBO studies

W. Lamine,[†,‡] F. Jérôme,[†] G. Frapper,[†] and F. Guégan[*,†]

†*IC2MP UMR 7285, Université de Poitiers – CNRS, 4, rue Michel Brunet TSA 51106–86073 Cedex 9 Poitiers, France.*

‡*Present address: Université de Pau et des Pays de l'Adour, E2S UPPA, CNRS, IPREM, UMR 5254, 64053 Pau cedex 09, France*

E-mail: frederic.guegan@univ-poitiers.fr

S1 NBO analysis of the lone-electron orbitals in products P1-P1'

In Table 1, we provide the NBO characteristics of the C-H bond involved in the formation of products **P1** to **P1'**: number of electrons on the bonding σ(C-H) orbital, energy of this orbital, contribution of C atomic orbitals, and decomposition into s and p carbon AOs.

In Table 2, we provide the characteristic of the lone-electron NBO on the radical carbon: population, energy, s-p contributions and the two principal second-order stabilisation (with neighbouring antibonding C-H and C-O orbitals).

Table S1: NBO characteristics of the C-H bonds involved in the formation of products **P1** to **P1'**.

End product	Population	Energy (u.a.)	C contrib.	s-p contrib.
**P1**	1.9835	-0.55820	61.12%	27.5% s - 72.5% p
**P2**	1.9769	-0.52660	62.24%	25.1% s - 74.8% p
**P3**	1.9784	-0.53875	61.90%	24.7% s - 75.3% p
**P4**	1.9792	-0.52948	61.96%	24.8% s - 74.2% p
**P5**	1.9806	-0.53394	62.18%	25.3% s - 74.6% p
**P1'**	1.9787	-0.54302	62.08%	25.2% s - 74.7% p

Table S2: Characteristics of the lone-electron (spin-up) NBO on radical carbon in **P1** to **P1'**. Nature of the acceptor NBO are indicated along with the second-order stabilisation.

Product	Population	Energy (u.a.)	s-p contrib.	2nd order stab. (kcal/mol)
**P1**	0.9525	-0.29777	19.2% s - 80.8% p	3.84, σ*(C-H)
				1.76, σ*(C-O)
**P2**	0.9075	-0.24294	8.6% s - 91.4% p	5.01, σ*(C-H)
				4.68, σ*(C-H)
**P3**	0.9165	-0.25280	9.6% s - 90.4% p	5.03, σ*(C-H)
				4.99, σ*(C-H)
**P4**	0.9230	-0.24848	11.4% s - 88.6% p	4.70, σ*(C-H)
				4.49, σ*(C-H)
**P5**	0.8974	-0.21492	0.3% s - 99.7% p	11.45, σ*(C-H)
				4.36, σ*(C-H)
**P1'**	0.9281	-0.26574	10.5% s - 89.5% p	4.12, σ*(C-H)
				4.00, σ*(C-H)

In Figure 1 we provide a rudimentary illustration of the hyperconjugation interaction beneath the planarity of radical **P5**.

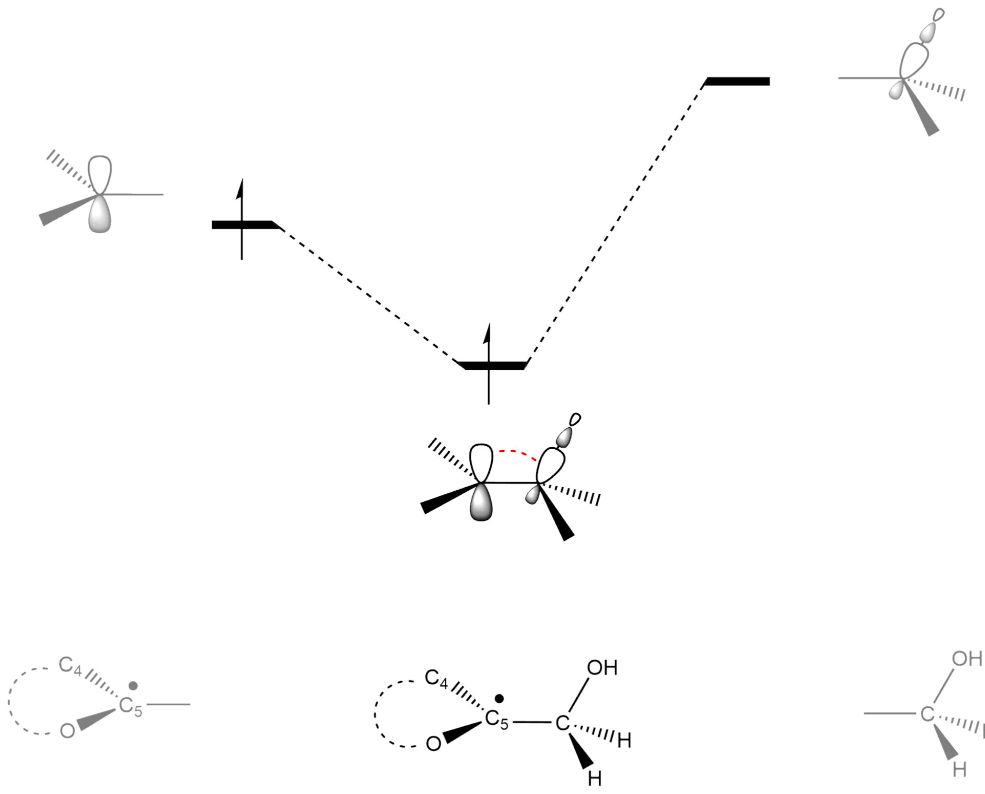

Figure S1: Schematic illustration of the hyperconjugation interaction between the lone-electron orbital and neighbouring anti-bonding σ^*(C-O) orbital in product **P5**.

S2 Reaction profiles for the hydrolysis reactions

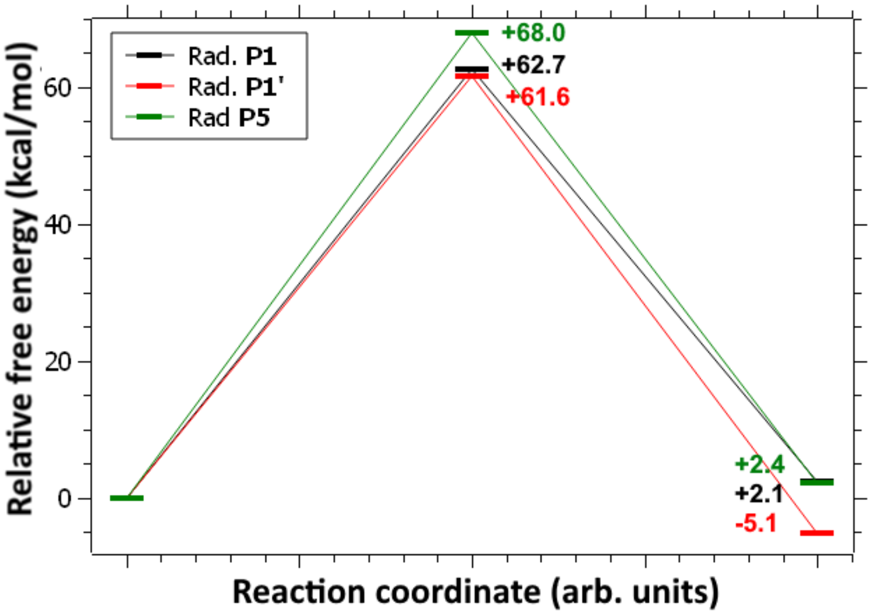

Figure S2: Reaction profiles for the hydrolysis of radicals P1 (black), P5 (green) and P1' (red), in free enthalpy and relative to the pre-reacting complex formed by one H_2O molecule and target radicals (*cf.* Scheme 1 in main manuscript).

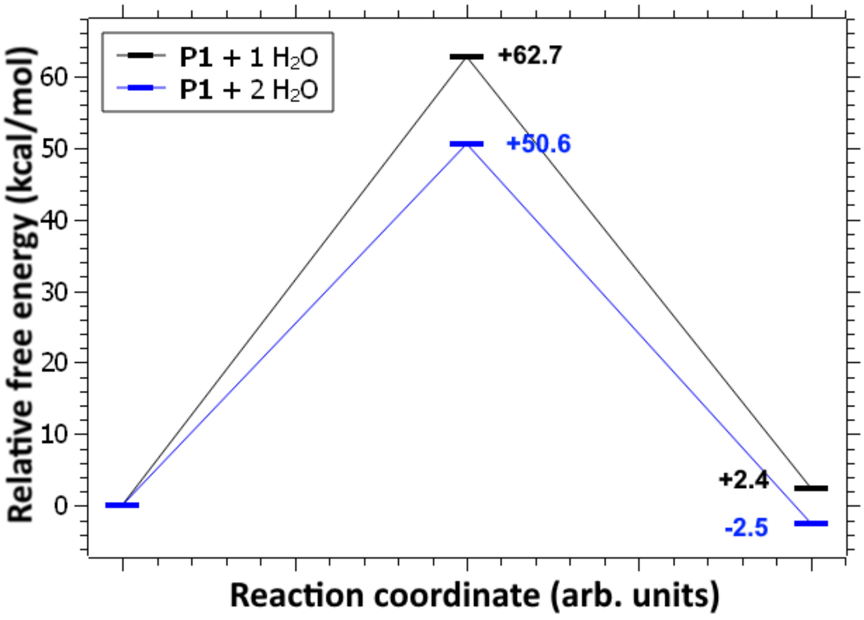

Figure S3: Reaction profiles for the hydrolysis of radical P1, involving one (black) and two (blue) explicit water molecules.

S3 Reaction profiles for the fragmentation reactions

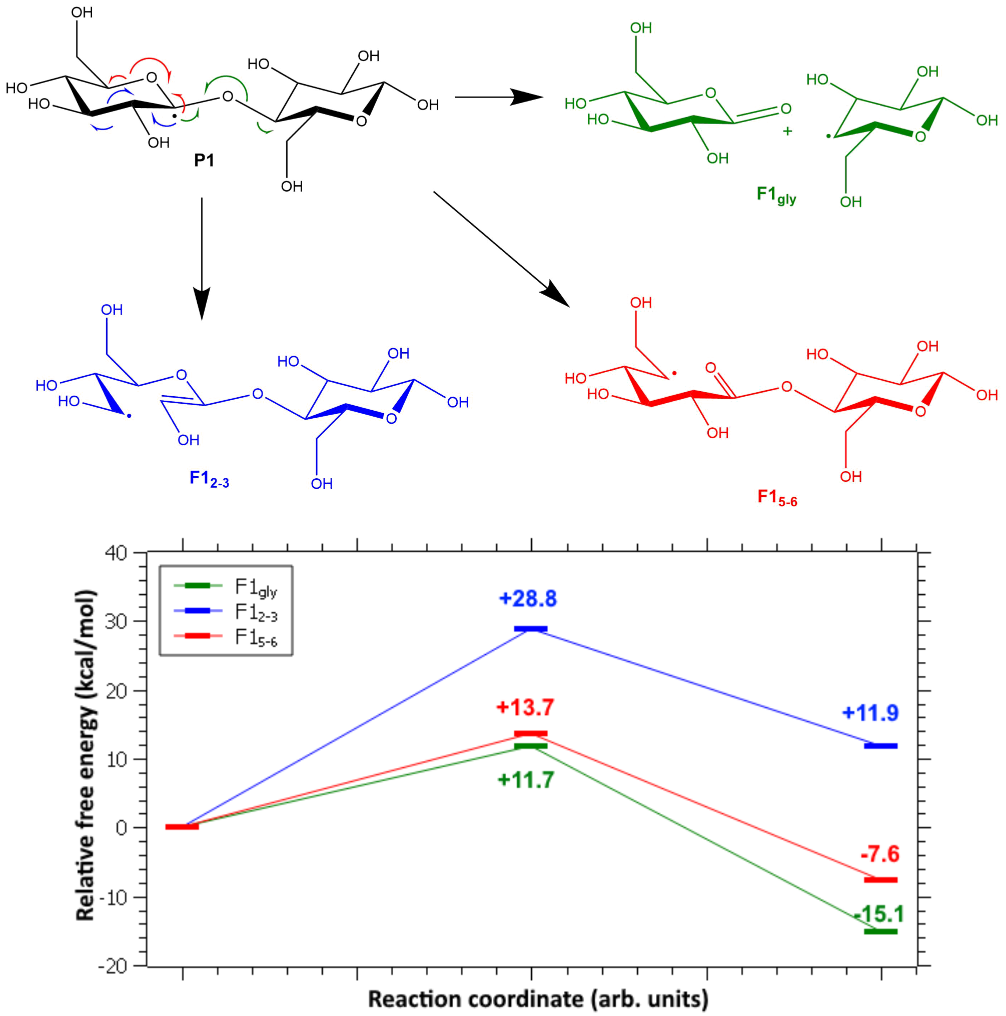

Figure S4: Top: studied fragmentation reactions for carboradical **P1**. Bottom: associated reaction profiles in free enthalpy (relative to **P1**).

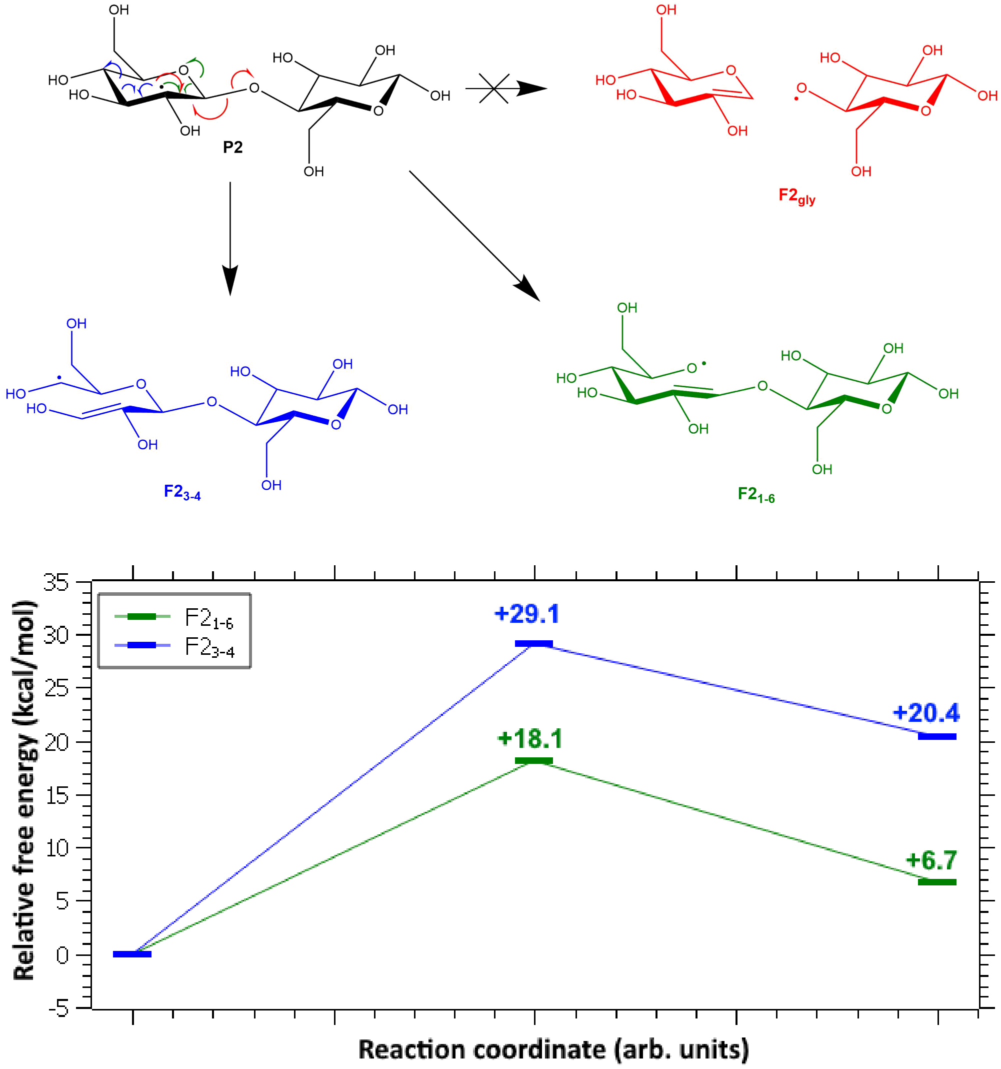

Figure S5: Top: studied fragmentation reactions for carboradical **P2**. Bottom: associated reaction profiles in free enthalpy (relative to **P2**). Transition state for the glycosidic bond cleavage (leading to $F2_{gly}$) could not be isolated.

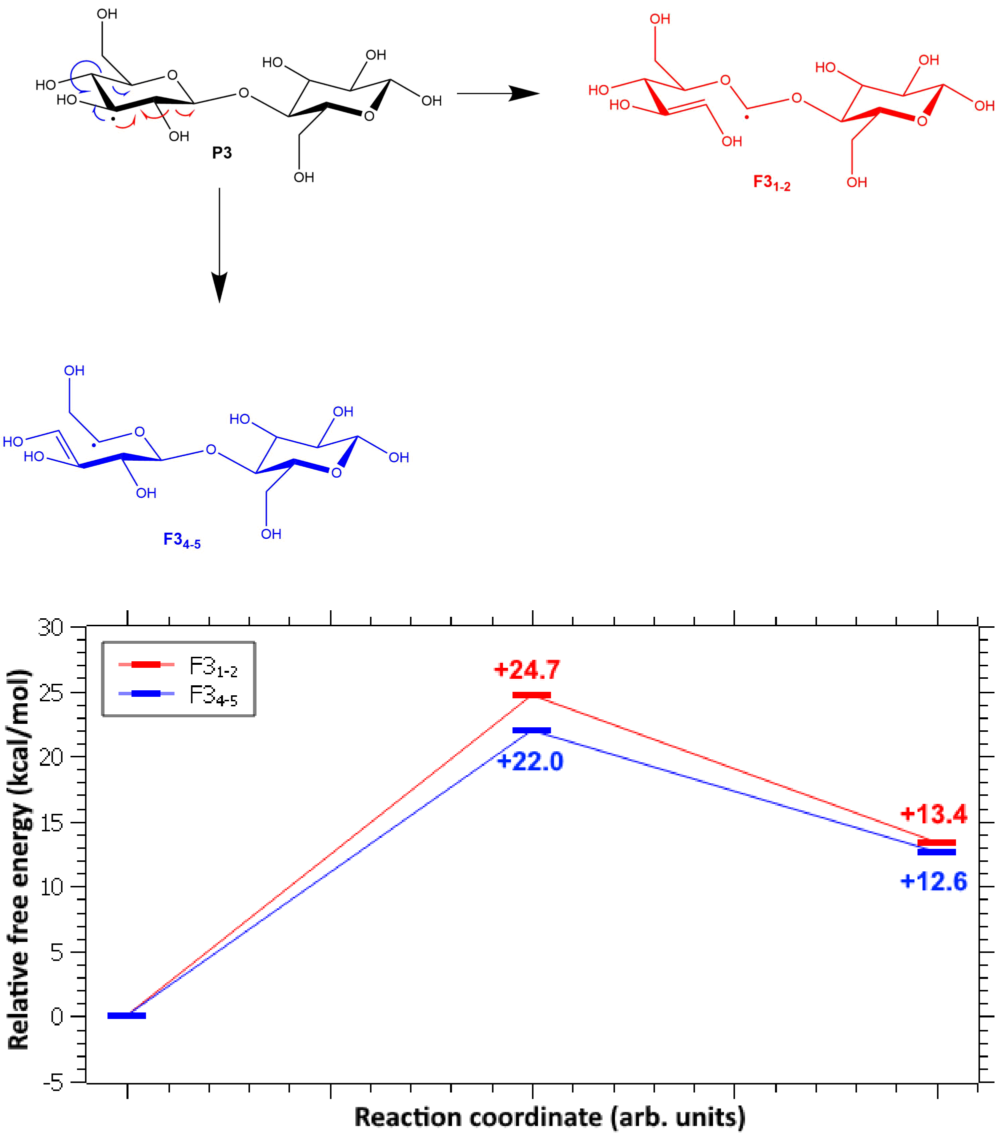

Figure S6: Top: studied fragmentation reactions for carboradical **P3**. Bottom: associated reaction profiles in free enthalpy (relative to **P3**).

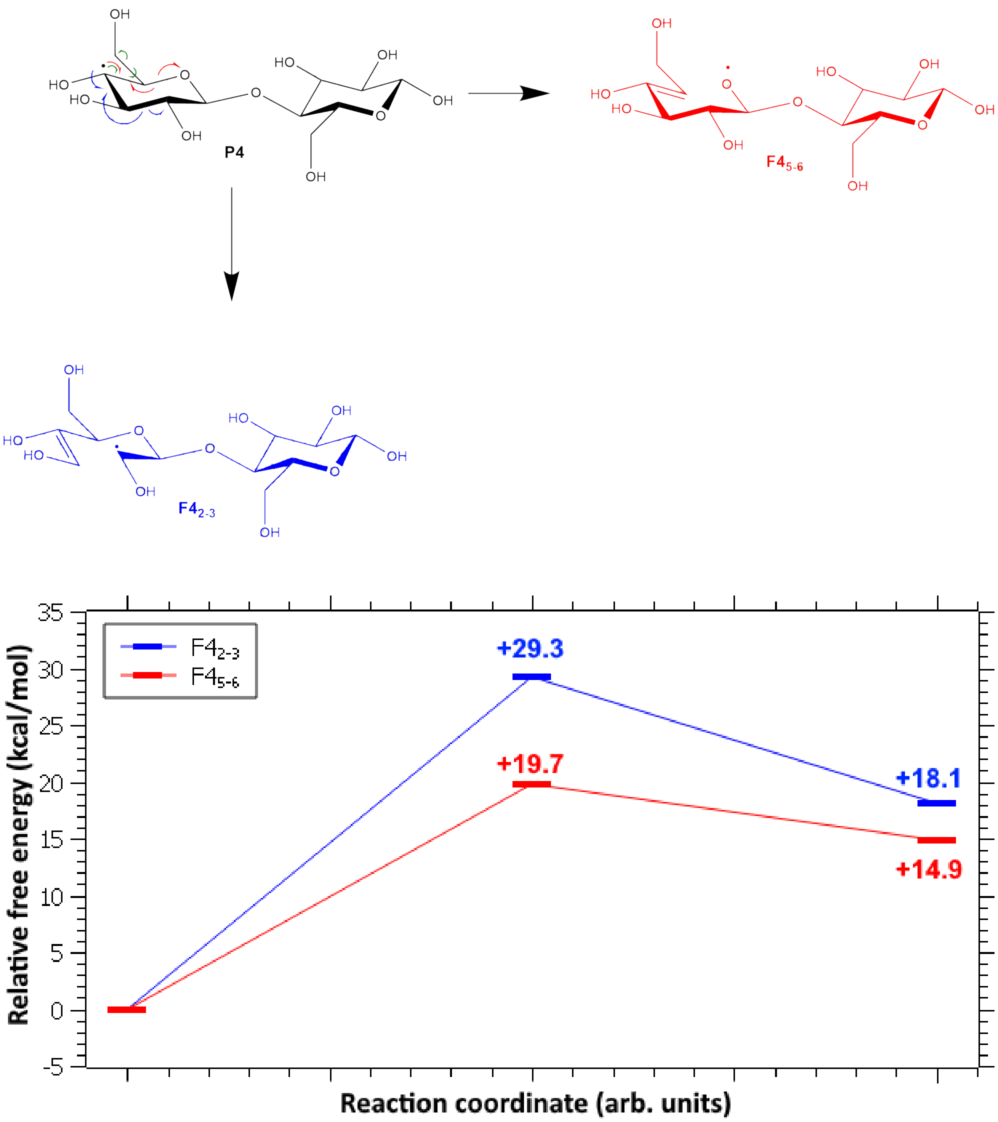

Figure S7: Top: studied fragmentation reactions for carboradical **P4**. Bottom: associated reaction profiles in free enthalpy (relative to **P4**).

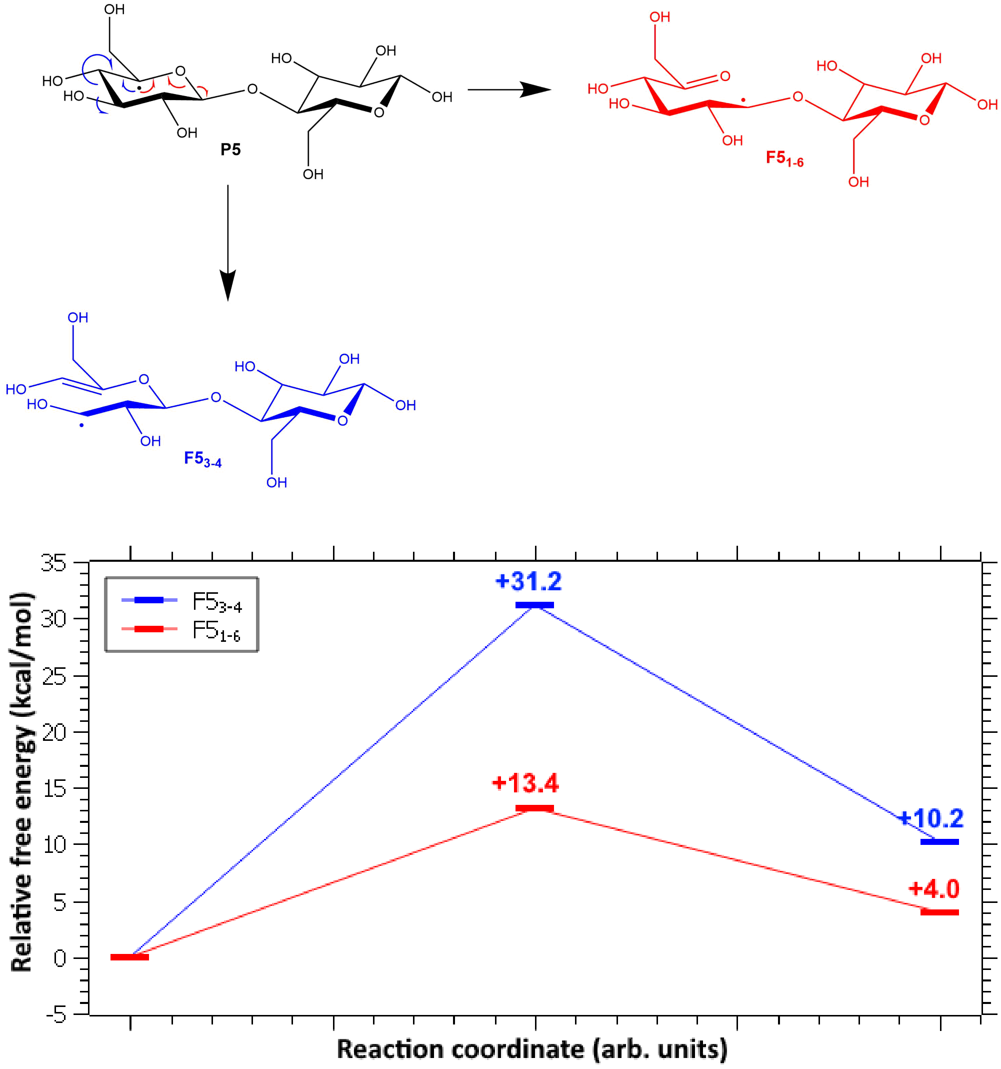

Figure S8: Top: studied fragmentation reactions for carboradical **P5**. Bottom: associated reaction profiles in free enthalpy (relative to **P5**).

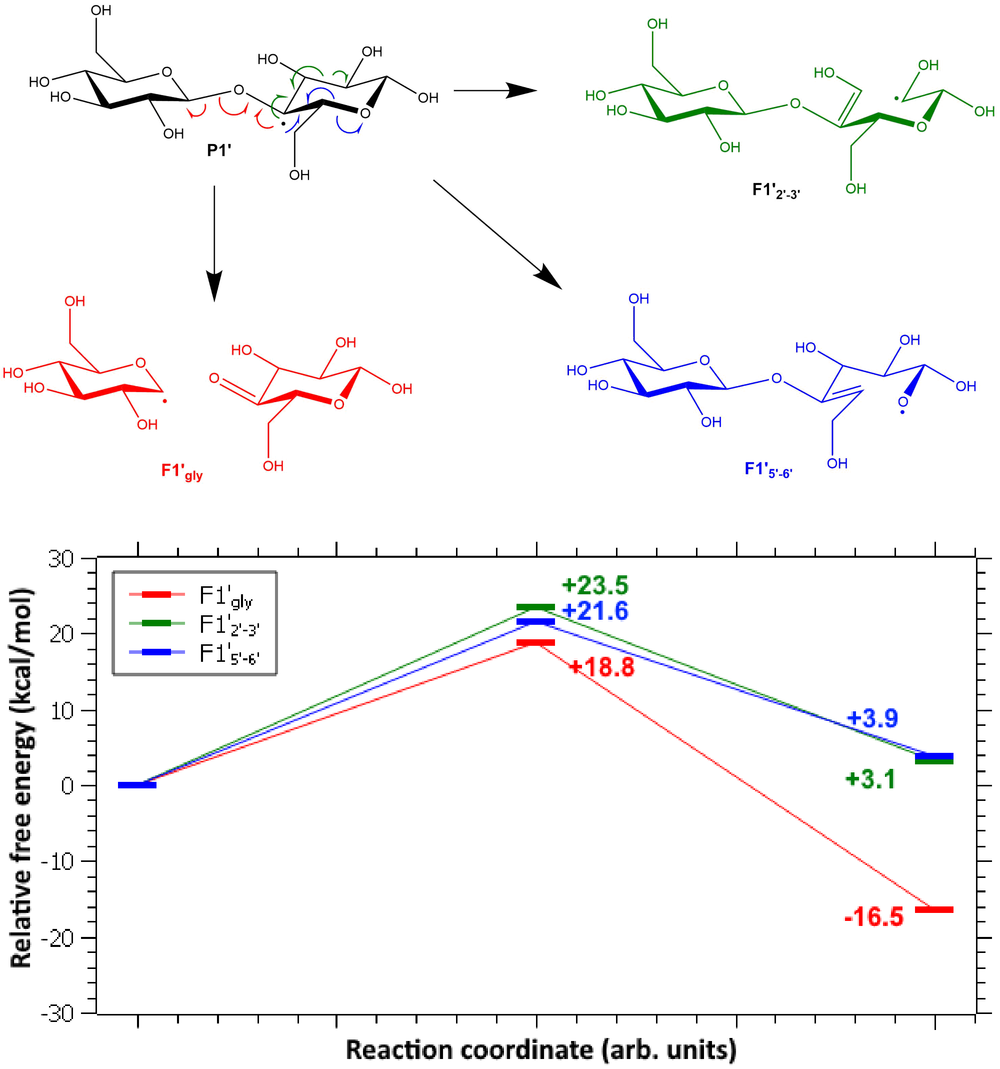

Figure S9: Top: studied fragmentation reactions for carboradical **P1'**. Bottom: associated reaction profiles in free enthalpy (relative to **P1'**).

S4 Geometries

In the following we provide the Cartesian coordinates for the optimised geometries of the reagents, transition states and products, along with their free enthalpy (300K, 1 atm) and first vibration frequency.

```
OH radical, G = -47531.87 kcal/mol, 3701.83 cm -1
O                  0.00000000    0.00000000    0.10889000
H                  0.00000000    0.00000000   -0.87112200

IB Network I, G =-814316.11 kcal/mol, 26.48cm -1
C                  0.90601100    0.40277800   -0.12063600
H                  0.80172800    0.39851500   -1.21867800
C                  1.84797400    1.52530300    0.32449300
H                  1.84798600    1.54382700    1.42433400
C                  3.26071000    1.24624300   -0.17795200
H                  3.26200400    1.30016300   -1.27809100
C                  3.70375400   -0.14936100    0.24666200
H                  3.74185500   -0.18853900    1.34586000
C                  2.69526700   -1.20108500   -0.24160100
H                  2.65084600   -1.18691000   -1.34143200
C                  3.04151800   -2.62409500    0.21159800
H                  3.22721800   -2.62676100    1.29679700
H                  2.18545100   -3.27595500    0.01462700
O                  1.43694500    2.77189700   -0.20772700
H                  0.50682900    2.93813700    0.04829500
O                  4.21127600    2.16929000    0.34722500
O                  4.98566300   -0.47159100   -0.29308700
O                  1.40317800   -0.87547800    0.30568200
O                  4.13596200   -3.16259100   -0.50971500
H                  4.86858500   -2.52831400   -0.45908300
O                 -0.31873000    0.63094300    0.49400900
C                 -4.31111000   -0.39364000    0.20370900
H                 -4.35106300   -0.27928800    1.30294100
C                 -3.34141300   -1.51628700   -0.16754700
H                 -3.31805000   -1.59251000   -1.26468200
C                 -1.94445400   -1.17984000    0.34339300
H                 -1.96584300   -1.14993700    1.44428100
C                 -1.52009000    0.20672800   -0.16075300
H                 -1.35635000    0.16251100   -1.24692200
C                 -2.60419000    1.25894500    0.15001100
H                 -2.66502000    1.39411600    1.24143400
```

C	-2.37621400	2.62900700	-0.49548300
H	-2.09990900	2.51025300	-1.55156700
H	-3.32849800	3.16668100	-0.44456000
O	-3.84692300	-2.71921300	0.40106200
H	-3.18866200	-3.41457900	0.25728300
O	-1.08083100	-2.21642000	-0.10131600
H	-0.16938800	-2.00497400	0.17419300
O	-3.85756100	0.81870200	-0.37298100
O	-1.35607100	3.35336600	0.21229500
H	-1.47685400	4.29601600	0.04168000
O	-5.59085300	-0.60716900	-0.31437300
H	3.90952600	3.06308200	0.13042700
H	5.58373800	0.26277100	-0.08943700
H	-5.78914400	-1.55038000	-0.21165400

R-C1, G = -861845.64 kcal/mol, 25.57 cm -1

C	0.87454000	-0.27426000	-0.03696400
H	0.76860000	-0.34913400	1.06232000
C	1.81762200	-1.37197500	-0.54788000
H	1.80276500	-1.34756100	-1.64580600
C	3.23928100	-1.12542600	-0.05244800
H	3.26567300	-1.25427000	1.04048900
C	3.67057300	0.29987400	-0.38564700
H	3.69027100	0.41495700	-1.48025500
C	2.66762900	1.31143500	0.19002300
H	2.62976100	1.21491000	1.28521200
C	3.00782900	2.76459700	-0.16035700
H	3.18530700	2.84798600	-1.24389100
H	2.15093100	3.39662900	0.09010900
O	1.40260600	-2.65086800	-0.07383000
H	0.50042400	-2.85194700	-0.40834800
O	4.18335100	-2.00001800	-0.66603500
O	4.95909100	0.58626800	0.15435900
O	1.37164600	1.02487300	-0.37259400
O	4.10512100	3.25282300	0.59058400
H	4.84151800	2.62984600	0.48624600
O	-0.34804200	-0.45702500	-0.66834500
C	-4.35955700	0.43153000	-0.24113100
H	-4.41547100	0.40023800	-1.34556800
C	-3.41217100	1.55034700	0.19767400
H	-3.36665200	1.54305700	1.29655300
C	-2.01795800	1.29398600	-0.36563000
H	-2.06368100	1.34424200	-1.46526200
C	-1.55192400	-0.11163900	0.03175500
H	-1.37290200	-0.14682500	1.11451200

C	-2.61044900	-1.16882400	-0.34098500
H	-2.69373000	-1.20735400	-1.44191200
C	-2.30240400	-2.58002500	0.18535400
H	-1.91690400	-2.53434100	1.20648800
H	-3.23815000	-3.14925000	0.19330700
O	-3.96420500	2.77702600	-0.26745400
H	-3.32940600	3.48143100	-0.07195600
O	-1.17086400	2.31919100	0.13453100
H	-0.25951000	2.14966400	-0.16502700
O	-3.86579900	-0.81078700	0.23117400
O	-1.30220500	-3.27302100	-0.57823200
H	-1.66153500	-3.50948300	-1.44332800
O	-5.63462000	0.57152200	0.31008900
H	3.93404600	-2.91194700	-0.46057100
H	5.55739400	-0.12707900	-0.11331500
H	-5.85339000	1.51572700	0.29481600
O	0.60419700	-1.97843700	2.52461500
H	0.98435600	-2.55959800	1.82170700

TS-Abs-C1, G=-861844.60 kcal/mol, i 106.51cm -1

C	0.92105200	0.34357300	-0.17849500
H	0.80481200	0.33533400	-1.31529400
C	1.85830100	1.51004600	0.17643200
H	1.82695900	1.64449200	1.26726600
C	3.28982100	1.20443800	-0.25646400
H	3.33706700	1.20706700	-1.35544200
C	3.71456100	-0.17139500	0.24496800
H	3.69805200	-0.17097600	1.34590800
C	2.73360200	-1.24166100	-0.25544100
H	2.71236100	-1.24749600	-1.35405100
C	3.06922900	-2.65285300	0.24021600
H	3.22315200	-2.63137700	1.33038000
H	2.22032300	-3.31024700	0.03142100
O	1.45077700	2.69342500	-0.49336800
H	0.53514600	2.93020500	-0.22988500
O	4.21997300	2.14738200	0.27147000
O	5.02002200	-0.50757400	-0.21891000
O	1.42114900	-0.91054300	0.25199100
O	4.18457500	-3.20242800	-0.43708900
H	4.91705300	-2.56952900	-0.37214200
O	-0.29836200	0.57836400	0.42837700
C	-4.31152800	-0.39127800	0.17752100
H	-4.36628000	-0.16642900	1.25937300
C	-3.35877900	-1.56409200	-0.06086900
H	-3.31516200	-1.74951900	-1.14396000

C	-1.96270000	-1.20837200	0.44260400
H	-2.00354500	-1.06457200	1.53431500
C	-1.50496900	0.10609300	-0.20022500
H	-1.32274500	-0.04575700	-1.27079600
C	-2.56825700	1.20748800	-0.01232900
H	-2.64722600	1.43739100	1.06532500
C	-2.27335200	2.50646500	-0.77967900
H	-1.88870400	2.28218300	-1.77713100
H	-3.21519400	3.05503700	-0.88777400
O	-3.90275700	-2.69284900	0.61435900
H	-3.26856000	-3.42020600	0.53636600
O	-1.11943400	-2.30702300	0.12658100
H	-0.20000200	-2.07503100	0.34511600
O	-3.82390200	0.75066300	-0.50709000
O	-1.28161100	3.33394100	-0.15265300
H	-1.64901500	3.72757700	0.64948300
O	-5.58653800	-0.63155000	-0.33767100
H	3.98109200	3.02598300	-0.05586500
H	5.60398200	0.24094400	-0.02546500
H	-5.79916300	-1.56056600	-0.16062600
O	0.65571700	1.01786000	-2.77205300
H	0.96208300	1.90187500	-2.47425000

P-C1, G = -861867.44 kcal/mol, 19.32cm -1

C	0.90457700	-0.22911200	-0.29646600
H	0.33104700	-2.76995500	3.21364400
C	1.84049400	-1.37932600	-0.59273000
H	1.96152900	-1.47636900	-1.68743900
C	3.21339900	-1.12536600	0.02405200
H	3.12790000	-1.20940100	1.11747400
C	3.69199500	0.27876700	-0.32410000
H	3.77949800	0.36351200	-1.41887400
C	2.67850200	1.32003500	0.16838900
H	2.55700400	1.24016600	1.25595800
C	3.06603800	2.75952600	-0.18833300
H	3.32405200	2.81734100	-1.25723400
H	2.20410100	3.41021900	-0.01461500
O	1.35372300	-2.60911500	-0.06075500
H	0.48275000	-2.82743600	-0.46252200
O	4.19800300	-2.03475900	-0.45980200
O	4.94979600	0.56238700	0.28364900
O	1.40532500	1.05379400	-0.47018500
O	4.11291500	3.24561000	0.63199500
H	4.84750200	2.61353600	0.58731100
O	-0.32523400	-0.37886800	-0.85090100

C	-4.31969500	0.47664000	-0.20123400
H	-4.41102800	0.49921200	-1.30361600
C	-3.35817100	1.57120600	0.26228400
H	-3.27332400	1.50593000	1.35670200
C	-1.98560200	1.34593500	-0.36367700
H	-2.07371700	1.44910500	-1.45731400
C	-1.49951500	-0.07483400	-0.05461300
H	-1.24335300	-0.17012900	1.00612000
C	-2.57358800	-1.11608300	-0.43342700
H	-2.69514000	-1.10411300	-1.53216100
C	-2.26505400	-2.55051300	0.02380800
H	-1.87206200	-2.55336200	1.04267400
H	-3.20240500	-3.11706100	0.00202300
O	-3.92250400	2.82154500	-0.11845300
H	-3.27686000	3.51290200	0.08785100
O	-1.12947600	2.35731200	0.15127600
H	-0.22621800	2.19924900	-0.17174300
O	-3.80901900	-0.78350000	0.19527600
O	-1.26958700	-3.20855300	-0.77787600
H	-1.60340700	-3.33164500	-1.67618100
O	-5.57737100	0.58793200	0.39577600
H	3.90300900	-2.93487900	-0.26216900
H	5.54153500	-0.18152300	0.09582900
H	-5.79204300	1.53204300	0.44137400
O	0.08515800	-2.20063100	2.47500300
H	0.67552500	-2.43382500	1.73317600

R-C2, G = -861846.73 kcal/mol, 22.98cm -1

C	0.60430700	-0.27366500	0.16365100
H	0.46856200	-0.23102100	1.25716400
C	1.61582000	-1.35541000	-0.21493900
H	1.64480100	-1.40482000	-1.31390600
C	3.00392300	-0.99665600	0.30729700
H	2.99586300	-1.02255600	1.40638800
C	3.40794100	0.40752400	-0.14697200
H	3.49749000	0.41633500	-1.24396400
C	2.31030700	1.41174300	0.26069800
H	2.23324400	1.43433700	1.35873700
C	2.59536500	2.83757900	-0.22886200
H	2.84984900	2.81271400	-1.30013800
H	1.68623800	3.43521000	-0.11351300
O	1.28067200	-2.61278300	0.34497400
H	0.37940500	-2.87678600	0.06763400
O	3.95209300	-1.96571500	-0.17088200
O	4.61776700	0.85486800	0.45504900

O	1.05153600	1.00416100	-0.30657300
O	3.60296400	3.47343100	0.53503000
H	4.36048000	2.86652100	0.57538300
O	-0.59041000	-0.58649200	-0.47470700
C	-4.64454500	0.18885600	-0.23698700
H	-4.66949200	0.06897100	-1.33633300
C	-3.74848500	1.37212600	0.13520700
H	-3.73717400	1.45466500	1.23205300
C	-2.32949100	1.12188600	-0.36501600
H	-2.34096700	1.08494400	-1.46596800
C	-1.82742900	-0.23228200	0.15362000
H	-1.68698900	-0.17219100	1.24204200
C	-2.83906800	-1.35162400	-0.16369600
H	-2.88736400	-1.47833000	-1.25984900
C	-2.51083000	-2.70828500	0.48031900
H	-2.21141100	-2.57327300	1.52293300
H	-3.42276700	-3.31546800	0.46693700
O	-4.32615500	2.53675400	-0.44323900
H	-3.71980600	3.27628400	-0.29192900
O	-1.53258300	2.21057600	0.07927100
H	-0.61046100	2.05983500	-0.19918500
O	-4.12125500	-0.99366800	0.34511500
O	-1.43065400	-3.40573100	-0.15042800
H	-1.71171800	-3.71874500	-1.02020800
O	-5.93778400	0.32402700	0.27130100
H	3.57706400	-2.84259900	0.00682900
H	5.37645900	0.35730100	0.09030800
H	-6.19427600	1.25274200	0.16490200
O	6.50975800	-1.06375500	-0.51855500
H	5.68849600	-1.62019200	-0.44308500

TS-Abs-C2, G= -861843.21 kcal/mol, i 86.62cm -1

C	0.88168800	0.37506600	-0.11503500
H	0.78350900	0.34324100	-1.21424900
C	1.81539700	1.52698300	0.28421400
H	1.78405400	1.59864300	1.41910700
C	3.25783200	1.23651400	-0.11843100
H	3.33983100	1.28376100	-1.21464600
C	3.68061300	-0.15201000	0.34942800
H	3.67014300	-0.17639100	1.44824700
C	2.68976000	-1.20828300	-0.16506500
H	2.67706500	-1.20137900	-1.26609200
C	3.02659300	-2.62839700	0.30563900
H	3.19178400	-2.62356500	1.39370600
H	2.17296700	-3.27965800	0.09687200

O	1.42986200	2.73866500	-0.29985700
H	0.49110000	2.93619400	-0.08787500
O	4.16287700	2.18153600	0.46482900
O	4.98111800	-0.48507300	-0.13849200
O	1.38262200	-0.88386500	0.34378200
O	4.13238500	-3.17329700	-0.39275000
H	4.87050200	-2.54844000	-0.31609400
O	-0.34607100	0.61229600	0.48659200
C	-4.33816800	-0.39775600	0.14718200
H	-4.38669800	-0.31314100	1.24886500
C	-3.36710800	-1.51329900	-0.24500400
H	-3.33507000	-1.56200900	-1.34353600
C	-1.97387000	-1.19281700	0.28668500
H	-2.00503600	-1.18724300	1.38754100
C	-1.54341200	0.20291600	-0.18424500
H	-1.37427200	0.18340400	-1.27032500
C	-2.62642300	1.24968700	0.14483500
H	-2.69506100	1.34217900	1.24324500
C	-2.38204100	2.64118900	-0.46021800
H	-2.13087800	2.55432400	-1.52062700
H	-3.31560800	3.20938200	-0.38052500
O	-3.88120500	-2.72756300	0.28955600
H	-3.22477400	-3.42225100	0.13459900
O	-1.10592900	-2.21987300	-0.17235900
H	-0.20425900	-2.03287800	0.14732000
O	-3.87808600	0.83025400	-0.39216100
O	-1.30157200	3.36321500	0.14092900
H	-1.51933900	3.57135500	1.05962500
O	-5.61316800	-0.59422900	-0.38711700
H	3.95591000	3.05507000	0.10147200
H	5.58754300	0.22449000	0.11878600
H	-5.81740900	-1.53834000	-0.30630100
O	2.41720300	1.94676500	2.89459700
H	3.27257000	2.26564600	2.53644200

P-C2, G = -861871.45 kcal/mol, 21.66cm-1

C	-0.56317100	-0.27232900	-0.37524700
H	-0.31937100	-0.17152500	-1.45419900
C	-1.58424400	-1.33860500	-0.12952800
H	-7.10200700	-1.22956200	0.80754100
C	-3.03566000	-1.05263000	-0.33585500
H	-3.30679900	-1.14054500	-1.40478000
C	-3.37211700	0.37565000	0.11670500
H	-3.33305700	0.40618700	1.21489600
C	-2.32104700	1.35943100	-0.42831700

H	-2.30373200	1.31678600	-1.52880000
C	-2.58977900	2.81174600	-0.01282400
H	-2.76664900	2.85349800	1.07329800
H	-1.69995100	3.40919500	-0.23329800
O	-1.23316500	-2.62961500	-0.37778900
H	-0.26925900	-2.78246200	-0.23415700
O	-3.84601600	-1.99645500	0.39720300
O	-4.64404200	0.80442100	-0.35224100
O	-1.02842400	0.99530900	0.09305100
O	-3.66032200	3.38325900	-0.74122100
H	-4.40963400	2.76804900	-0.66722300
O	0.59570100	-0.61970000	0.32708600
C	4.64689400	0.19924800	0.40079900
H	4.59388000	0.03870200	1.49381100
C	3.76833400	1.38779600	0.00659400
H	3.83408100	1.50741800	-1.08496400
C	2.31833000	1.11329700	0.39421800
H	2.24933700	1.03912500	1.49117500
C	1.86564300	-0.22576100	-0.20459700
H	1.79763900	-0.12547500	-1.29741200
C	2.86550100	-1.34705200	0.14059700
H	2.83612000	-1.51638600	1.23160500
C	2.59789100	-2.67781900	-0.57841700
H	2.41100800	-2.50603000	-1.64169900
H	3.49670200	-3.29772600	-0.49006700
O	4.29260000	2.53641700	0.66273600
H	3.68580900	3.27334800	0.50031300
O	1.55209700	2.21440200	-0.07132700
H	0.61063200	2.05126500	0.12490900
O	4.17793700	-0.96478600	-0.26047500
O	1.45145300	-3.38541500	-0.08682200
H	1.63690700	-3.72325900	0.79962700
O	5.97213500	0.36185100	-0.00724800
H	-3.40960600	-2.85943000	0.34648500
H	-5.34732400	0.34142300	0.15343300
H	6.20841000	1.29000500	0.14229000
O	-6.26641400	-0.87327300	1.13186000
H	-5.58090100	-1.55176100	0.97395600

R-C3, G = -861844.27 kcal/mol, 22.13 cm -1

C	-0.66850000	0.42569800	-0.06981400
H	-0.64139800	0.41805300	1.03232800
C	-1.56435800	1.55732600	-0.58176300
H	-1.49475300	1.56643900	-1.67977000
C	-3.00756200	1.29557500	-0.16428400

H	-3.07158300	1.36065500	0.93307200
C	-3.44153200	-0.09757600	-0.60442200
H	-3.43970900	-0.14222700	-1.70248800
C	-2.47778000	-1.16809000	-0.06775900
H	-2.49956800	-1.17014000	1.03248500
C	-2.79728800	-2.58102800	-0.57207700
H	-2.86985500	-2.56821400	-1.67026300
H	-1.97363300	-3.24528600	-0.29655200
O	-1.17899100	2.80297600	-0.03276600
H	-0.22941800	2.95713400	-0.21603900
O	-3.92057500	2.21654700	-0.75560400
O	-4.76938700	-0.38505000	-0.12819600
O	-1.15493300	-0.84603700	-0.53413200
O	-3.96417600	-3.11956300	0.02587400
H	-4.70455700	-2.52206600	-0.15634100
O	0.59686400	0.63527800	-0.60012600
C	4.55432500	-0.41008100	-0.03021500
H	4.68021500	-0.27041800	-1.11997800
C	3.55600300	-1.53755000	0.23774900
H	3.44949200	-1.64175600	1.32758500
C	2.20249400	-1.18200300	-0.36864800
H	2.30667800	-1.12714100	-1.46388500
C	1.74654300	0.19421600	0.13395400
H	1.50186900	0.12776600	1.20329500
C	2.85370800	1.24791900	-0.07233900
H	2.99980200	1.40337800	-1.15296800
C	2.57655100	2.60695000	0.57702700
H	2.19581600	2.47227900	1.59800000
H	3.53347900	3.13598000	0.63002600
O	4.10054000	-2.72729400	-0.32225600
H	3.43692400	-3.42701700	-0.23665200
O	1.30323400	-2.22355100	-0.01471400
H	0.41448000	-1.99598100	-0.34393100
O	4.06071100	0.79078400	0.53755100
O	1.63662300	3.35404600	-0.21407300
H	1.74595300	4.29218300	-0.01313000
O	5.78905800	-0.64090500	0.58088200
H	-3.64091000	3.11168400	-0.51572800
H	-5.34348900	0.33516200	-0.43441300
H	5.99511200	-1.58125500	0.46777900
O	-4.83135200	-0.25391500	2.76728700
H	-4.88749700	-0.42387500	1.79531600

TS-Abs-C3, G= -861842.89 kcal/mol, i 70.51cm -1

C	0.88719200	0.44545300	-0.13145900

H	0.77618000	0.46202500	-1.22709000
C	1.81679500	1.56614600	0.33934400
H	1.84507200	1.52948600	1.43980900
C	3.22272600	1.32423400	-0.20049800
H	3.18211700	1.41101500	-1.32743700
C	3.70995100	-0.08226200	0.12891400
H	3.84068200	-0.16808500	1.21769000
C	2.68917400	-1.13762700	-0.33221600
H	2.60105900	-1.10727000	-1.42722200
C	3.06352600	-2.56134600	0.09510600
H	3.27233000	-2.57799800	1.17600200
H	2.21128300	-3.22019700	-0.09322600
O	1.38839400	2.83184500	-0.11842500
H	0.45092100	2.96602400	0.13181700
O	4.16936500	2.24440500	0.29956900
O	4.95899400	-0.34786900	-0.52608500
O	1.41804200	-0.83034400	0.26935600
O	4.14844100	-3.07996300	-0.65644500
H	4.88001800	-2.44547300	-0.60996600
O	-0.33268300	0.63721100	0.50302600
C	-4.31931900	-0.41332800	0.20862500
H	-4.36333800	-0.30288100	1.30813800
C	-3.33971400	-1.52784500	-0.16272700
H	-3.31343900	-1.60250600	-1.25981800
C	-1.94663800	-1.18059500	0.35162700
H	-1.97075600	-1.15158300	1.45252600
C	-1.53419300	0.20947900	-0.15107300
H	-1.37224400	0.16968500	-1.23737800
C	-2.62543800	1.25226100	0.16505700
H	-2.68938900	1.38042800	1.25719800
C	-2.40450000	2.62807200	-0.47029800
H	-2.10693700	2.52004300	-1.52160800
H	-3.36407200	3.15371700	-0.43269500
O	-3.83749400	-2.73513300	0.40368100
H	-3.17759800	-3.42762600	0.25413300
O	-1.07196700	-2.20869000	-0.09130800
H	-0.16167200	-1.98262000	0.17456400
O	-3.87413000	0.80475800	-0.36255800
O	-1.40680300	3.36034300	0.26184000
H	-1.52085500	4.30083900	0.07459800
O	-5.59567400	-0.63545300	-0.31385200
H	3.83023800	3.13850700	0.14163000
H	5.58555500	0.33810800	-0.24924600
H	-5.78730100	-1.58029500	-0.21352600
O	3.59779000	0.94833300	-2.91239200

H	4.33695900	0.36492200	-2.63787200

P-C3, G = -861867.46 kcal/mol, 22.04 cm-1

C	-0.71150700	0.39459600	-0.17754200
H	-0.71143300	0.41588900	0.92323200
C	-1.60282800	1.50462900	-0.73706200
H	-1.43013400	1.52695000	-1.83135300
C	-3.02915800	1.19073800	-0.43088100
H	-3.88597300	1.07471600	2.95816500
C	-3.52438800	-0.20360600	-0.61884600
H	-3.71169500	-0.41011900	-1.69003000
C	-2.48671100	-1.22163000	-0.09582500
H	-2.46684400	-1.17013000	1.00147600
C	-2.79040300	-2.65933500	-0.53041800
H	-2.90574300	-2.69251500	-1.62530000
H	-1.94185200	-3.29488300	-0.26180700
O	-1.29515400	2.77310700	-0.17659200
H	-0.34885500	2.96889800	-0.33793600
O	-3.96062100	2.16581700	-0.66950300
O	-4.75577600	-0.43643400	0.10030200
O	-1.19234900	-0.88201300	-0.62675300
O	-3.92276500	-3.19871800	0.13048900
H	-4.64588800	-2.55467000	0.08765700
O	0.56136800	0.59949600	-0.69176700
C	4.51910900	-0.35021800	0.00174800
H	4.67326500	-0.21294200	-1.08469400
C	3.53832100	-1.49870900	0.24661800
H	3.39946600	-1.59832600	1.33320700
C	2.19804600	-1.17618600	-0.40514700
H	2.33677900	-1.12260700	-1.49670600
C	1.69795700	0.19078900	0.07800800
H	1.42404100	0.12223200	1.13995500
C	2.78496600	1.26970900	-0.09944400
H	2.95996200	1.42605900	-1.17567400
C	2.45186700	2.62297100	0.53648100
H	2.03288600	2.48115300	1.54108800
H	3.39369200	3.17320800	0.62723900
O	4.12731300	-2.67925500	-0.28734400
H	3.47742200	-3.39345900	-0.21736600
O	1.30808000	-2.23369100	-0.07705500
H	0.42263100	-2.01957100	-0.42337900
O	3.98395600	0.84316300	0.54794900
O	1.52665300	3.35233700	-0.29048000
H	1.64593800	4.29538200	-0.12151800
O	5.74059600	-0.55128200	0.64884700

H	-3.52156200	3.02632200	-0.56206800
H	-5.40571000	0.21755600	-0.19688500
H	5.96682000	-1.48882600	0.55195300
O	-3.95501100	0.11302700	2.91704300
H	-4.35915700	-0.07721600	2.05351600

R-C4, G = -861846.64 kcal/mol, 22.03cm -1

C	0.60895500	-0.28494400	0.16907500
H	0.48648400	-0.26311700	1.26502700
C	1.61964500	-1.35353900	-0.24562400
H	1.64034200	-1.37285700	-1.34548100
C	3.00977700	-1.00725600	0.27751600
H	3.00997600	-1.06202900	1.37555200
C	3.40619300	0.40969700	-0.14379100
H	3.49108900	0.44439000	-1.24058200
C	2.30769100	1.40197200	0.29051900
H	2.23434400	1.40016300	1.38908000
C	2.58767500	2.83921000	-0.16779500
H	2.83875800	2.83928300	-1.24009300
H	1.67750400	3.43164000	-0.03554000
O	1.27780500	-2.62525200	0.27793100
H	0.35893000	-2.84547400	0.01865700
O	3.95958400	-1.95864500	-0.23342500
O	4.61725800	0.84615400	0.46390300
O	1.04803900	1.00300900	-0.28078300
O	3.59642700	3.46003500	0.60713600
H	4.35566400	2.85441100	0.63069200
O	-0.58883500	-0.59807800	-0.46035000
C	-4.63844400	0.19537100	-0.25832700
H	-4.65238200	0.05509300	-1.35515900
C	-3.74320200	1.38151700	0.10319500
H	-3.74377700	1.48240700	1.19857700
C	-2.31980600	1.11717000	-0.37693700
H	-2.31931800	1.06430400	-1.47712000
C	-1.82503200	-0.23138300	0.16415200
H	-1.68437700	-0.15585200	1.25168900
C	-2.83973200	-1.35188600	-0.14250000
H	-2.87520300	-1.51085400	-1.23190200
C	-2.54003100	-2.69339300	0.53261100
H	-2.28753000	-2.53981300	1.59004500
H	-3.45664000	-3.28935700	0.47809200
O	-4.30808400	2.53923500	-0.50118600
H	-3.69660600	3.27643000	-0.35938300
O	-1.52604600	2.21110500	0.06084000
H	-0.60083000	2.05489500	-0.20387800

O	-4.12440000	-0.97540100	0.35258900
O	-1.46652200	-3.36781600	-0.14649400
H	-1.54072400	-4.31422800	0.02980000
O	-5.93704900	0.34332800	0.23439700
H	3.58767100	-2.84221700	-0.08638800
H	5.37597500	0.36073600	0.08337900
H	-6.19031800	1.27002000	0.10534200
O	6.51367400	-1.03713000	-0.56771100
H	5.69401600	-1.59756000	-0.50773800

TS-Abs-C4, G= -861843.25 kcal/mol, i 123.97cm -1

C	0.91377100	0.35995300	-0.07369100
H	0.82879400	0.37167800	-1.17372400
C	1.85540000	1.46471900	0.41154400
H	1.84724500	1.44457500	1.51036500
C	3.26936700	1.19973800	-0.09258100
H	3.28531800	1.31762600	-1.18729700
C	3.71296700	-0.21774900	0.25123700
H	3.79848500	-0.26674500	1.39521400
C	2.68257300	-1.26576100	-0.19765800
H	2.63745300	-1.26870100	-1.29898400
C	3.02242800	-2.68663600	0.27439800
H	3.23450400	-2.67351300	1.35299400
H	2.15114300	-3.32557500	0.10580000
O	1.46114300	2.73119800	-0.08600800
H	0.52732500	2.89630500	0.15914100
O	4.21976900	2.09490100	0.49225000
O	4.96543200	-0.53995400	-0.31232100
O	1.39767000	-0.92546800	0.34485200
O	4.08792900	-3.25479200	-0.46759100
H	4.85298000	-2.66378600	-0.39840000
O	-0.31778600	0.58923700	0.52347500
C	-4.31432700	-0.39422900	0.15844500
H	-4.37070400	-0.29777800	1.25859700
C	-3.34921800	-1.52000000	-0.21603200
H	-3.31039000	-1.57946400	-1.31380400
C	-1.95693800	-1.20422100	0.32067500
H	-1.99356500	-1.19225900	1.42132000
C	-1.51358500	0.18613900	-0.15497800
H	-1.33521800	0.15908200	-1.23942300
C	-2.59232600	1.24257100	0.15980100
H	-2.66858600	1.35698200	1.25258800
C	-2.34076600	2.62287900	-0.45414500
H	-2.04447600	2.52328400	-1.50675300
H	-3.28920200	3.16781500	-0.41082700

O	-3.87403500	-2.72630100	0.32653400
H	-3.21973900	-3.42558900	0.18370000
O	-1.09547900	-2.24081000	-0.12888400
H	-0.19020700	-2.05047300	0.17870500
O	-3.84094800	0.82332300	-0.39076300
O	-1.32849800	3.32440700	0.28783200
H	-1.44352900	4.27175900	0.14114700
O	-5.58733500	-0.58700100	-0.38372100
H	3.92214200	2.99913700	0.31319000
H	5.59543800	0.15638400	-0.07218800
H	-5.79908400	-1.52853200	-0.29303500
O	4.20786700	0.23676100	2.83074400
H	4.40569200	1.15599200	2.55409300

P-C4, G = -861870.89 kcal/mol, 20.19 cm-1

C	0.59677200	-0.27819400	0.13678100
H	0.48634300	-0.26215700	1.23499900
C	1.60721700	-1.33593900	-0.30003900
H	1.65485300	-1.30867500	-1.39764100
C	2.99430200	-1.02866000	0.26004700
H	2.98188000	-1.24583900	1.34596100
C	3.34949000	0.41319100	0.03325800
H	7.03938400	-0.88269200	-0.88448400
C	2.26465300	1.43899400	0.27296100
H	2.10823500	1.58739500	1.35985900
C	2.59308700	2.81636300	-0.33153500
H	2.89358200	2.68888300	-1.38111300
H	1.68679000	3.42810300	-0.30466800
O	1.24915000	-2.62514100	0.16763300
H	0.32938600	-2.82441400	-0.10326300
O	3.95413400	-1.91991100	-0.34851100
O	4.57927000	0.87799700	0.39887500
O	1.02051800	1.00999500	-0.31535400
O	3.57331800	3.51129800	0.41808600
H	4.35783500	2.94344000	0.46608100
O	-0.60913700	-0.59668000	-0.47868700
C	-4.65583400	0.19707500	-0.21366300
H	-4.68590700	0.07756000	-1.31261000
C	-3.75403200	1.37540000	0.15705400
H	-3.73823100	1.45528900	1.25408200
C	-2.33838300	1.12004100	-0.34880300
H	-2.35379600	1.08503700	-1.44969400
C	-1.83762600	-0.23794600	0.16300500
H	-1.68645500	-0.18115500	1.25044700
C	-2.85661300	-1.35191800	-0.15225500

H	-2.90704300	-1.48950700	-1.24396900
C	-2.54442700	-2.70654800	0.48981800
H	-2.25249300	-2.57290400	1.53986000
H	-3.46676800	-3.29550700	0.45737000
O	-4.32715500	2.54559500	-0.41566500
H	-3.70859400	3.27715200	-0.27505000
O	-1.53766200	2.20618100	0.09490800
H	-0.61509200	2.04859600	-0.18094700
O	-4.13498900	-0.98584000	0.36731300
O	-1.50064900	-3.37347800	-0.24003900
H	-1.55385300	-4.31923300	-0.05277300
O	-5.94752200	0.33777600	0.30004000
H	3.60690900	-2.81636800	-0.22094900
H	5.31550400	0.23674600	0.22489400
H	-6.19940800	1.26770900	0.19378600
O	6.48450800	-0.96696200	-0.09944000
H	5.76502600	-1.59526100	-0.31321500

R-C5, G = -861847.00 kcal/mol, 10.13cm -1

C	0.62761700	-0.70721800	0.05683200
H	0.58327700	-0.55018000	1.14731900
C	1.37601200	-2.00335400	-0.27118300
H	1.31337400	-2.15735400	-1.35864700
C	2.83939600	-1.87970100	0.14045800
H	2.89214700	-1.79685900	1.23757400
C	3.45305800	-0.62987700	-0.47855400
H	3.43931200	-0.73388900	-1.57376100
C	2.63245500	0.61168100	-0.09477900
H	2.64941600	0.73829000	0.99882400
C	3.15339400	1.89723300	-0.74446500
H	3.24312500	1.75612800	-1.83042500
H	2.44420400	2.70870300	-0.56107300
O	0.82290400	-3.09871900	0.43413700
H	-0.13367300	-3.16199400	0.23330500
O	3.61893400	-2.98954200	-0.29458200
O	4.79510300	-0.43238500	-0.02886900
O	1.28214500	0.42376500	-0.54840300
O	4.39778100	2.30783900	-0.18331300
H	4.99177000	1.53785900	-0.15223300
O	-0.64479200	-0.82430100	-0.48354800
C	-4.44374500	0.77013000	-0.18788000
H	-4.55516400	0.51896700	-1.25904600
C	-3.31639500	1.78772400	-0.00594700
H	-3.22457800	2.00021600	1.06938500
C	-2.00543100	1.19866600	-0.51621500

H	-2.08842200	1.03352800	-1.60207100
C	-1.74291200	-0.15770500	0.15330300
H	-1.51440500	0.00239600	1.21648700
C	-2.97192500	-1.08126000	0.02783000
H	-3.11114500	-1.34625700	-1.03220500
C	-2.88770200	-2.37593800	0.84176700
H	-2.52303300	-2.16610700	1.85585100
H	-3.90489000	-2.77382300	0.91392500
O	-3.68907000	2.96451900	-0.71366400
H	-2.94524200	3.58310100	-0.67543000
O	-0.98846100	2.15238300	-0.24273600
H	-0.13064600	1.78537500	-0.52329000
O	-4.12408000	-0.40708300	0.53312000
O	-2.02689400	-3.32280800	0.18587800
H	-2.27145400	-4.21078300	0.47572100
O	-5.65529800	1.22313800	0.33884600
H	3.20537300	-3.79613400	0.04524800
H	5.27681900	-1.26396200	-0.15194600
H	-5.73242600	2.16360600	0.11709600
O	4.38228400	4.24899800	1.84848500
H	4.44888000	3.58044700	1.11958400

TS-Abs-C5, G=-861844.58 kcal/mol, i 140.37cm -1

C	0.89501400	0.43746900	-0.13528100
H	0.78340700	0.44274600	-1.22997300
C	1.82374600	1.56649400	0.32529200
H	1.82576300	1.57004900	1.42561400
C	3.24316300	1.32249400	-0.18186600
H	3.24759200	1.38804900	-1.28000900
C	3.70571900	-0.06864800	0.23250300
H	3.72104300	-0.12336400	1.33312900
C	2.71178800	-1.12260600	-0.27496600
H	2.66397300	-1.05366700	-1.42444500
C	3.10735100	-2.56708000	0.03806800
H	3.38559300	-2.66056400	1.09846200
H	2.25210000	-3.22069800	-0.15150900
O	1.39185500	2.81197000	-0.18869400
H	0.45872100	2.96246200	0.06742200
O	4.17538000	2.25270400	0.36324200
O	4.99960700	-0.37475300	-0.27819800
O	1.42512600	-0.85327100	0.25605300
O	4.15750200	-3.00977100	-0.81248900
H	4.90754500	-2.40454100	-0.68971800
O	-0.32275800	0.61868100	0.50076400
C	-4.31787500	-0.40992600	0.21695700

H	-4.36297500	-0.27415100	1.31359400
C	-3.34541400	-1.53825800	-0.12827200
H	-3.31723500	-1.63656900	-1.22335900
C	-1.94979500	-1.19081900	0.38043500
H	-1.97469000	-1.13783200	1.48053500
C	-1.52556200	0.18526400	-0.15119300
H	-1.35832900	0.12136000	-1.23521800
C	-2.61180300	1.24118200	0.14072900
H	-2.67744500	1.39116100	1.23001000
C	-2.38180400	2.60373000	-0.51972900
H	-2.07105500	2.47555500	-1.56491900
H	-3.34154600	3.12997900	-0.50447000
O	-3.85194800	-2.72992400	0.46290500
H	-3.19981400	-3.43153200	0.32210000
O	-1.08753400	-2.23831000	-0.03985000
H	-0.17140200	-2.01080200	0.19879400
O	-3.86214600	0.79153400	-0.38062300
O	-1.39299200	3.35020000	0.21017600
H	-1.51611400	4.28868100	0.01899000
O	-5.59491500	-0.63446700	-0.30250800
H	3.87603600	3.14398100	0.13336000
H	5.57326600	0.38646000	-0.10472700
H	-5.79082200	-1.57678700	-0.18772800
O	2.74528400	-1.38078800	-2.91685300
H	3.33919100	-2.14984700	-2.78427500

P-C5, G = -861869.32 kcal/mol, 21.73 cm-1

C	-0.87988300	0.40498200	-0.16624300
H	-0.85817100	0.26003300	0.92060600
C	-1.83117900	1.52705100	-0.57457900
H	-1.78269100	1.64276700	-1.66848800
C	-3.24907900	1.16294100	-0.15140600
H	-3.26616500	1.03895800	0.94141900
C	-3.67704700	-0.15626500	-0.79986100
H	-3.84019700	0.04595500	-1.87464400
C	-2.63489900	-1.22128100	-0.61493000
H	-2.12102500	-1.71939000	2.30888800
C	-2.95053800	-2.59678500	-0.15510800
H	-3.75950400	-3.03901600	-0.74717700
H	-2.06507200	-3.23059700	-0.23012400
O	-1.49329100	2.73648500	0.07964600
H	-0.54146200	2.92564500	-0.05022900
O	-4.19824900	2.14896500	-0.54527800
O	-4.89898300	-0.63886500	-0.22878800
O	-1.31556800	-0.86713300	-0.75569700

O	-3.34607000	-2.63173100	1.24993500
H	-4.17908500	-2.13493700	1.30000100
O	0.37106400	0.70044600	-0.68220800
C	4.37874100	-0.19278300	-0.17012400
H	4.52485900	0.14484700	-1.21300400
C	3.44477300	-1.40302200	-0.14068400
H	3.31566700	-1.70338800	0.90943100
C	2.08650000	-1.02492200	-0.72294900
H	2.21328400	-0.76486800	-1.78621200
C	1.53282200	0.20870100	0.00379400
H	1.26777400	-0.06130500	1.03451500
C	2.57860300	1.34259500	0.01479800
H	2.74303800	1.68544100	-1.01906700
C	2.20027400	2.55830200	0.86531900
H	1.75236000	2.24032100	1.81583000
H	3.12767500	3.09956200	1.07880800
O	4.07523100	-2.44458700	-0.87920000
H	3.44689100	-3.17770000	-0.95069300
O	1.25580800	-2.17028800	-0.59746900
H	0.34443100	-1.93157800	-0.84204600
O	3.79749200	0.85851200	0.58039000
O	1.28872100	3.40008100	0.13829200
H	1.31002400	4.28263200	0.52981400
O	5.60964200	-0.45991800	0.43557500
H	-3.89080200	3.00230600	-0.20615600
H	-5.55389800	0.06989100	-0.30728700
H	5.87264300	-1.35405000	0.16952900
O	-1.49742200	-1.07481800	2.70441300
H	-1.24207200	-1.43580300	3.56124400

R-C1’, G = -861844.78 kcal/mol, 29.75cm -1

C	-0.92479500	0.46886100	0.02849700
H	-0.81327500	0.37226300	1.12045300
C	-1.87530200	1.61487200	-0.32567500
H	-1.89276800	1.70930300	-1.42159500
C	-3.27812700	1.29183800	0.17764900
H	-3.26146100	1.25907200	1.27816200
C	-3.72080700	-0.06814600	-0.35137400
H	-3.77540200	-0.01685900	-1.44951300
C	-2.70339000	-1.15339700	0.03488700
H	-2.64083900	-1.23316500	1.12991700
C	-3.05163500	-2.53176900	-0.53915800
H	-3.24464500	-2.43955000	-1.61938900
H	-2.19363600	-3.19799600	-0.40710000
O	-1.45916300	2.82330600	0.28409200

H	-0.53394600	3.00866200	0.02508400
O	-4.24102300	2.24721200	-0.25977300
O	-4.99314900	-0.44091500	0.17801300
O	-1.41648200	-0.77632300	-0.49991900
O	-4.13931000	-3.13460500	0.13854600
H	-4.86881900	-2.49494900	0.16163900
O	0.29659800	0.75211200	-0.57601000
C	4.27574000	-0.31597000	-0.39410700
H	4.28521000	-0.17355500	-1.49109300
C	3.30682700	-1.44532200	-0.02947300
H	3.31592300	-1.55487800	1.06473600
C	1.90544800	-1.07390800	-0.49845800
H	1.89669000	-1.02935100	-1.59694100
C	1.50270700	0.30427900	0.04685300
H	1.35967900	0.23407200	1.13583900
C	2.59148400	1.35505000	-0.24970400
H	2.62933200	1.53322600	-1.33603000
C	2.39087000	2.69745200	0.46025700
H	2.16262400	2.53138600	1.52114200
H	3.33941400	3.23966300	0.38931400
O	3.79259900	-2.62833500	-0.65526500
H	3.15964600	-3.34242800	-0.49506900
O	1.01222500	-2.10568700	-0.06394200
H	0.11400900	-1.88790900	-0.39059400
O	3.85034900	0.88168800	0.22796300
O	1.33902000	3.44534500	-0.17122900
H	1.45405700	4.37827900	0.04980100
O	5.56597800	-0.55537100	0.08215900
H	-3.94275300	3.12269000	0.02565400
H	-5.59652500	0.30573300	0.04788300
H	5.76275200	-1.49179600	-0.07240700
O	0.40766700	-1.46850100	2.60900700
H	0.64814200	-2.01434900	1.81980200

TS-Abs-C1’, G= -861843.16 kcal/mol, i 276.13 cm-1

C	0.93503800	0.46344000	-0.16131500
H	0.85498100	0.51891200	-1.25601300
C	1.86756300	1.54575800	0.38720500
H	1.82655200	1.50110900	1.48588700
C	3.29495700	1.27472800	-0.07658600
H	3.33903700	1.38549400	-1.17125600
C	3.70207800	-0.14679600	0.29449300
H	3.69250900	-0.24101000	1.39102600
C	2.70465000	-1.15921500	-0.28947700
H	2.70762300	-1.08793700	-1.38739700

C	3.01741900	-2.60712000	0.10619800
H	3.16225200	-2.66579700	1.19605600
H	2.16250700	-3.23902900	-0.15309600
O	1.49121200	2.82598800	-0.08568700
H	0.54272300	2.97204900	0.10381800
O	4.23554000	2.15443600	0.53334300
O	5.00116100	-0.46163100	-0.20601100
O	1.39022000	-0.84625800	0.21795400
O	4.13064700	-3.12432900	-0.60046500
H	4.86833400	-2.50295500	-0.49295000
O	-0.31256300	0.69148700	0.42877200
C	-4.25053300	-0.39929200	0.26677200
H	-4.17957800	-0.33908900	1.36927600
C	-3.31010600	-1.48519700	-0.26042700
H	-3.37992000	-1.48228100	-1.35716900
C	-1.87839300	-1.17333600	0.16284800
H	-1.80315900	-1.27063200	1.25740400
C	-1.50345200	0.27190900	-0.20190400
H	-1.35314300	0.27527000	-1.37105000
C	-2.59603500	1.29786100	0.15047200
H	-2.59087000	1.43531000	1.24537800
C	-2.41191200	2.66922500	-0.50979600
H	-2.18948200	2.54694700	-1.57670400
H	-3.36440800	3.19914800	-0.40565700
O	-3.76123700	-2.72827600	0.26753800
H	-3.14894900	-3.42015500	-0.02055100
O	-1.03088200	-2.12788200	-0.46792000
H	-0.11634200	-1.96856700	-0.16126300
O	-3.86927900	0.84475900	-0.29214100
O	-1.36156000	3.39631400	0.14970100
H	-1.44570700	4.32890300	-0.08680900
O	-5.57194100	-0.60919600	-0.12890200
H	3.95994000	3.06310200	0.34541700
H	5.59793300	0.25686000	0.05132800
H	-5.74272600	-1.56184300	-0.07450000
O	-1.01471600	-0.08319100	-2.72440300
H	-0.94731400	-1.04826700	-2.57182700

P-C1’, G = -861864.67 kcal/mol, 27.29 cm-1

C	-0.85410300	0.48109200	-0.06579100
H	-0.67935800	0.32664300	1.00624100
C	-1.80631500	1.65076300	-0.32237700
H	-1.86504200	1.80326900	-1.41059500
C	-3.19111800	1.30480800	0.21640700
H	-3.13159200	1.21274200	1.31170000

C	-3.66184800	-0.02205600	-0.36736100
H	-3.75982300	0.08845200	-1.45808500
C	-2.63543500	-1.12765000	-0.07736900
H	-2.52064900	-1.26319300	1.00673700
C	-3.01004700	-2.47255700	-0.71021800
H	-3.24405600	-2.32463500	-1.77605400
H	-2.15071500	-3.14717500	-0.64642400
O	-1.36971600	2.82629800	0.33449300
H	-0.45468800	3.03335600	0.05510500
O	-4.16238800	2.28841400	-0.13199600
O	-4.91514900	-0.41882900	0.19090800
O	-1.36767700	-0.72473600	-0.64781100
O	-4.07419800	-3.10805300	-0.02361500
H	-4.79619400	-2.46586000	0.06707200
O	0.35473800	0.80619700	-0.71910300
C	4.27433900	-0.35269700	-0.37077100
H	4.28358000	-0.24218700	-1.47200400
C	3.28248800	-1.44336300	0.04017000
H	3.24347300	-1.46052500	1.13823800
C	1.89968300	-1.10934400	-0.50951600
H	1.92018500	-1.26843600	-1.60398600
C	1.55685100	0.33458300	-0.24179000
H	-0.21282000	-2.08236800	3.30671500
C	2.64779300	1.37971500	-0.30452700
H	2.79228500	1.70517700	-1.35363000
C	2.36562400	2.63719800	0.53320000
H	2.00880200	2.35240000	1.52985600
H	3.31761400	3.16818100	0.63363500
O	3.77595200	-2.67960100	-0.46533400
H	3.13349900	-3.37290200	-0.25838400
O	0.97553400	-2.04856900	0.05805000
H	0.09446100	-1.87323000	-0.33200200
O	3.87335700	0.86833500	0.21854700
O	1.39676100	3.46500100	-0.13190000
H	1.52345700	4.37773100	0.15614700
O	5.56124400	-0.60670200	0.10697600
H	-3.84317500	3.14512400	0.18605600
H	-5.51808900	0.33660200	0.12647200
H	5.71473600	-1.56082800	0.02971000
O	-0.22567900	-1.44837500	2.58008600
H	0.35440200	-1.80632200	1.88273500

R-C1-Hydro-1H2O, G=-861868.07 kcal/mol, 20.77cm -1

C	-0.94051500	-0.26172000	0.00012600
C	-1.80917800	-1.50133600	0.05377100

H	-1.96563300	-1.77287900	1.11797500
C	-3.17789700	-1.20917800	-0.55986600
H	-3.05065800	-1.08854200	-1.64571000
C	-3.75334400	0.07445300	0.02129300
H	-3.86765800	-0.04675500	1.11039300
C	-2.79926500	1.24492600	-0.24499600
H	-2.65788600	1.37333400	-1.32549100
C	-3.27721600	2.57061700	0.35779700
H	-3.55640500	2.41639900	1.41175600
H	-2.45275700	3.28912800	0.33016300
O	-1.26006600	-2.59166700	-0.65357300
H	-0.37673400	-2.83048700	-0.28966000
O	-4.11166900	-2.24984200	-0.29233100
O	-5.01387300	0.39387700	-0.56239900
O	-1.51719300	0.94338000	0.36409600
O	-4.33669600	3.13967600	-0.38883200
H	-5.03240000	2.46984700	-0.48262600
O	0.29072100	-0.40693900	0.58119400
C	4.25576800	0.64131200	-0.12034300
H	4.39193200	0.61374800	0.97683200
C	3.23062900	1.71443800	-0.49104900
H	3.11750600	1.70855800	-1.58493000
C	1.88757800	1.38686300	0.15503900
H	2.00034600	1.44467800	1.24964100
C	1.46295700	-0.04196300	-0.20536600
H	1.19451400	-0.10032200	-1.26571700
C	2.59544800	-1.04198700	0.10887200
H	2.76682300	-1.05362700	1.19756600
C	2.36685900	-2.48214300	-0.37859700
H	2.14289000	-2.47917400	-1.44914500
H	3.30652800	-3.02786300	-0.23118900
O	3.74509900	2.96597100	-0.05095300
H	3.07095800	3.64067800	-0.21666000
O	0.96507800	2.37233600	-0.29113100
H	0.08977300	2.17907800	0.08439000
O	3.78089300	-0.61637600	-0.56338100
O	1.28777600	-3.16077000	0.26157100
H	1.34861700	-3.05346600	1.23005100
O	5.48060300	0.83708500	-0.76269600
H	-3.73684100	-3.07701300	-0.62849600
H	-5.55258600	-0.41170500	-0.55700700
H	5.67219300	1.78677700	-0.73579000
O	1.04711900	-2.04942600	2.83420100
H	0.59146400	-2.23710800	3.66361800
H	0.56490300	-1.33253000	2.38700400

TS Hydro -C1 -1H2O, G= -861805.35 kcal/mol, i 227.93 cm -1

C	-1.14241100	0.01742500	0.03750200
C	-1.74210900	-1.36255300	0.26358400
H	-1.59933200	-1.61151500	1.32148300
C	-3.24138200	-1.38924100	-0.05204700
H	-3.37320000	-1.48111900	-1.13968400
C	-3.87269200	-0.08993200	0.43064400
H	-3.62821200	0.05284000	1.49402400
C	-3.32156400	1.07852600	-0.38271000
H	-3.61717700	0.98900900	-1.43168000
C	-3.70360600	2.46221000	0.14308300
H	-3.51784100	2.51461300	1.22636200
H	-3.08158700	3.21534000	-0.34833900
O	-1.10968300	-2.28399100	-0.59651100
H	-0.15074300	-2.33074100	-0.38706700
O	-3.91789200	-2.45132000	0.61249800
O	-5.28516100	-0.07705000	0.24869600
O	-1.81428500	1.01620400	-0.40102500
O	-5.04853200	2.77072500	-0.17707700
H	-5.61404200	2.04511500	0.13153900
O	0.89480700	-0.67949800	0.75916200
C	4.84045100	0.41957200	0.14265500
H	5.03884700	0.05454700	1.16849400
C	3.84924900	1.58572700	0.19849400
H	3.67144500	1.91428000	-0.83812500
C	2.54072600	1.11280600	0.82600300
H	2.72315800	0.85525500	1.87629300
C	1.98414600	-0.12869700	0.10051500
H	1.70842900	0.17968200	-0.92806400
C	3.10682500	-1.19010900	-0.05120200
H	3.33830400	-1.58857400	0.95329800
C	2.74186800	-2.35109400	-0.98635400
H	2.42539000	-1.95735800	-1.95615000
H	3.64401200	-2.95535300	-1.14517700
O	4.46388200	2.63198200	0.94686100
H	3.78525900	3.29216700	1.14939100
O	1.59085900	2.19381500	0.88241400
H	1.21724600	2.31478000	-0.00331700
O	4.28646800	-0.62323500	-0.62902600
O	1.66274500	-3.16532200	-0.52425700
H	1.87046400	-3.48878900	0.36273100
O	6.03764700	0.77310300	-0.49042300
H	-3.58265500	-3.29195800	0.27109000
H	-5.62405000	-0.92887700	0.56545000

H	6.29293200	1.64569800	-0.15512200
O	-0.81357200	0.54253800	2.11042300
H	-0.55976200	1.47860000	2.10896300
H	0.04166800	0.07861900	1.80400000

P-C1Hydro-1H2O, G=-861865.65 kcal/mol, 9.82 cm -1

C	-1.92852000	0.65699500	0.67295200
C	-1.94888000	-0.70663400	0.00972900
H	-2.09449700	-1.48051000	0.78530700
C	-3.14121000	-0.75635100	-0.95352600
H	-2.93267200	-0.07347300	-1.79045100
C	-4.41943000	-0.31180700	-0.25468600
H	-4.62586500	-1.00300800	0.57776200
C	-4.24397000	1.10170200	0.31353600
H	-4.04314200	1.80815900	-0.50215800
C	-5.45567400	1.59349800	1.11129700
H	-5.75684500	0.82307200	1.83782600
H	-5.16817100	2.48893500	1.66923600
O	-0.77184600	-0.97756400	-0.73222600
H	-0.21443800	-1.63856900	-0.26441700
O	-3.36532700	-2.07277000	-1.44399700
O	-5.52861300	-0.29249900	-1.15149800
O	-3.11758200	1.08907000	1.21948900
O	-6.52936300	1.96430600	0.26424400
H	-6.71228600	1.22079500	-0.33164900
O	1.70382200	-0.28962000	1.67685400
C	5.08848800	-0.21725300	-0.64502500
H	5.59484300	-0.45636700	0.30894300
C	4.40000100	1.14612000	-0.53806200
H	3.89487300	1.33033200	-1.50038100
C	3.37428200	1.12090400	0.59606200
H	3.90358400	1.02118300	1.55023200
C	2.41238400	-0.06544100	0.45377200
H	1.70111000	0.13110500	-0.36260000
C	3.17724000	-1.37135100	0.14337700
H	3.70678100	-1.66733500	1.06501800
C	2.25590900	-2.51072400	-0.31663900
H	1.94204200	-2.30878800	-1.34627900
H	2.81387900	-3.45317900	-0.30757300
O	5.41266500	2.12146100	-0.31770200
H	4.98432900	2.94783500	-0.05045600
O	2.69427900	2.37758300	0.69427000
H	2.10373500	2.47510100	-0.06757900
O	4.10780800	-1.19698800	-0.91952300
O	1.07398200	-2.64434600	0.48965000

H	1.20273800	-2.10870400	1.29503700
O	5.99236300	-0.27297400	-1.70952300
H	-2.55363600	-2.36476600	-1.88372400
H	-5.53906100	-1.13847300	-1.62368800
H	6.50386600	0.55005100	-1.69901900
O	-0.90047100	0.85300300	1.56906100
H	-1.09704400	1.64891400	2.08712200
H	0.86825900	0.21139100	1.66178200

R-C1-Hydro-2H2O, G= -909820.46 kcal/mol, 17.06cm -1

C	-0.86475600	0.08230900	0.55096600
C	-1.79551600	-0.74375100	1.40561000
H	-2.29110700	-0.07600900	2.12007600
C	-2.87063700	-1.40721600	0.52224800
H	-2.39834400	-2.16573500	-0.12350300
C	-3.52264400	-0.34435600	-0.35845100
H	-3.99900400	0.40330300	0.29392500
C	-2.46961900	0.35575600	-1.22302700
H	-1.99012500	-0.37113400	-1.89130500
C	-3.03183600	1.50557200	-2.06148500
H	-3.64195200	2.16249400	-1.42315000
H	-2.19836100	2.09594800	-2.45185300
O	-1.10584100	-1.72563500	2.18679800
H	-0.42529300	-2.12625500	1.62365700
O	-3.90421700	-2.00381200	1.29484400
O	-4.49497700	-0.91577200	-1.23373700
O	-1.45397900	0.93316400	-0.33751600
O	-3.76077800	1.03373900	-3.18179900
H	-4.43002600	0.40817000	-2.86235800
O	0.23298500	-0.63134800	0.08999600
C	4.20413900	0.27835600	-0.81993700
H	4.48964200	-0.70936600	-0.41267700
C	3.57784100	1.12907900	0.29044600
H	3.32658200	2.10940600	-0.14358000
C	2.30623500	0.44865200	0.80806700
H	2.58680400	-0.50085700	1.27973200
C	1.35142100	0.15863000	-0.35724600
H	0.97373300	1.09442100	-0.78857700
C	2.09803000	-0.62928100	-1.45039700
H	2.40249300	-1.60966100	-1.04985200
C	1.28088700	-0.84830100	-2.72076500
H	0.37663100	-1.41282800	-2.48404100
H	0.98705600	0.12891000	-3.13283500
O	4.55435600	1.26934700	1.31200000
H	4.14906200	1.72319800	2.06509100

O	1.72694800	1.22295800	1.86690200
H	1.11413200	1.88379700	1.51036100
O	3.25547500	0.10800800	-1.85314400
O	2.00185100	-1.61213700	-3.67090000
H	2.82922200	-1.14458700	-3.85698300
O	5.30191600	0.90577500	-1.41319500
H	-3.50018400	-2.60583900	1.93598500
H	-5.09655500	-1.45245400	-0.69655400
H	5.87207200	1.23228500	-0.70086300
O	-2.00503000	1.63009800	3.72153600
H	-2.43496000	1.99945700	4.50134400
H	-1.20951900	1.15209200	4.04139500
O	0.24165400	0.02120200	4.09785900
H	0.85939600	0.40744300	3.45233100
H	-0.16260300	-0.73079600	3.62836800

TS Hydro -C1 -2H2O, G= -909769.87 kcal/mol, i 309.24 cm -1

C	-1.26541600	0.11754500	1.36728000
C	-1.91728400	-1.25317800	1.33325700
H	-2.73761700	-1.24378800	2.06297400
C	-2.53348500	-1.50051700	-0.05389400
H	-1.73576200	-1.60444000	-0.80438900
C	-3.42327300	-0.31719900	-0.40206600
H	-4.14008600	-0.16374800	0.42119000
C	-2.56693300	0.93397600	-0.58302800
H	-1.96658300	0.84116300	-1.49217500
C	-3.35636100	2.24246000	-0.62486300
H	-4.05090200	2.28499600	0.22934300
H	-2.66188900	3.08332000	-0.54277100
O	-1.02821500	-2.28045200	1.68855500
H	-0.11910300	-1.92314700	1.50582700
O	-3.35300500	-2.65840900	-0.04730800
O	-4.13338500	-0.49693900	-1.62269100
O	-1.56598600	1.05762800	0.52616200
O	-4.03222900	2.39110300	-1.85923500
H	-4.52648100	1.57734500	-2.04775700
O	0.91947700	-0.60968700	1.32588500
C	4.40794000	0.19225600	-0.82807500
H	4.94938000	-0.49510300	-0.15171500
C	3.83961900	1.36394500	-0.02271100
H	3.32782300	2.02990200	-0.73623300
C	2.83907400	0.85436200	1.01462300
H	3.37264700	0.25800800	1.76402400
C	1.74673700	-0.02571200	0.37064100
H	1.16362900	0.62518100	-0.31644400

C	2.44109100	-1.09876800	-0.50739300
H	3.00213000	-1.78399600	0.14756500
C	1.46629500	-1.90777800	-1.35447000
H	0.78286900	-2.45514800	-0.70288800
H	0.88182600	-1.21504200	-1.98253600
O	4.94098100	2.04007800	0.57760500
H	4.58998600	2.67190000	1.22161100
O	2.30273600	1.96802400	1.75791900
H	1.64892800	2.41446900	1.19980100
O	3.34338500	-0.49124500	-1.44803700
O	2.13143400	-2.87268500	-2.15384500
H	2.83858900	-2.41103800	-2.62875700
O	5.24540700	0.62168400	-1.86554300
H	-2.81916500	-3.40149600	0.27273100
H	-4.50502500	-1.39288800	-1.61575700
H	5.85777900	1.27247500	-1.49109000
O	-1.69405700	0.76917000	3.10345000
H	-1.85020000	1.72207100	3.00900700
H	-0.71994400	0.65732000	3.49193200
O	0.70290900	0.36500100	3.71593600
H	1.28174000	1.14156000	3.72695700
H	0.90269600	-0.05973800	2.80887900

P-C1-Hydro-2H2O, G=-909823.00 kcal/mol, 10.65 cm -1

C	-2.31375900	0.41959600	1.57703900
C	-2.52498300	-1.02736100	1.16891900
H	-3.40272700	-1.41553000	1.71167800
C	-2.82864800	-1.07987800	-0.33601100
H	-1.91779800	-0.80277800	-0.89193700
C	-3.93473200	-0.09714100	-0.69555400
H	-4.84797100	-0.37846300	-0.14819800
C	-3.52808100	1.32060600	-0.27689900
H	-2.61572200	1.61764100	-0.81073400
C	-4.61314200	2.37136300	-0.53263800
H	-5.57616100	2.01419200	-0.13664200
H	-4.34755700	3.28713100	0.00288400
O	-1.46717900	-1.89931500	1.50373100
H	-0.60372400	-1.54005300	1.20524000
O	-3.26150400	-2.37391600	-0.73292100
O	-4.19616600	-0.08315100	-2.09810800
O	-3.26384900	1.31901200	1.14450900
O	-4.70226800	2.70944700	-1.90542300
H	-4.82398100	1.88870600	-2.40849300
O	1.15460500	-0.99715300	1.11979000
C	4.55319600	0.10692600	-1.03646000

H	5.16160700	-0.41542100	-0.27511900
C	3.80319600	1.27329500	-0.38196400
H	3.23875000	1.78130200	-1.18058800
C	2.83313900	0.75737100	0.68363600
H	3.40936400	0.35678400	1.52573100
C	1.92567700	-0.34532200	0.12104400
H	1.24991200	0.10795400	-0.62600700
C	2.78636300	-1.40609200	-0.59664800
H	3.41837400	-1.91446800	0.14802800
C	1.95315800	-2.44798800	-1.33530200
H	1.31835800	-2.97653100	-0.62151600
H	1.31124800	-1.93573700	-2.06958800
O	4.78188000	2.14590800	0.16840400
H	4.33344000	2.82217300	0.69590400
O	2.09786500	1.86287300	1.24929400
H	1.48300300	2.20139600	0.58092500
O	3.61258100	-0.78486800	-1.58937600
O	2.76875000	-3.42220700	-1.96430300
H	3.39317800	-2.95350300	-2.53714200
O	5.35134900	0.53125400	-2.10357700
H	-2.64480900	-3.02089000	-0.35763000
H	-4.31199200	-1.00089300	-2.38640500
H	5.88908300	1.27346700	-1.78951900
O	-2.04828000	0.62283800	2.91305800
H	-2.42381500	1.48286600	3.16216000
H	-0.13086100	0.46295000	3.42742000
O	0.84195000	0.42400900	3.40107700
H	1.14296500	1.26930600	3.03007100
H	1.14875700	-0.50273700	1.97925100

R-C1'-Hydro-1H2O, G= 861863.24 kcal/mol, 24.48cm -1

C	0.88765000	0.22701000	-0.22163400
H	0.68843000	-0.04994500	-1.26659000
C	1.81135100	1.44654500	-0.13846600
H	1.88174300	1.74765400	0.91703700
C	3.19962200	1.07561400	-0.65077000
H	3.13071500	0.84602400	-1.72585600
C	3.71523400	-0.15601900	0.08503100
H	3.82017500	0.08966600	1.15275900
C	2.71741500	-1.31550400	-0.05186800
H	2.60604400	-1.58418300	-1.11310200
C	3.13869300	-2.56806000	0.72612100
H	3.39977600	-2.28748700	1.75809100
H	2.29209200	-3.25953400	0.77007000
O	1.33037300	2.51042600	-0.94080400

H	0.45979400	2.79786500	-0.60425800
O	4.14462700	2.11908000	-0.44085900
O	4.97080400	-0.58571700	-0.43854000
O	1.44370900	-0.88734600	0.47830400
O	4.19494700	-3.26027500	0.08562300
H	4.91281000	-2.62792700	-0.07637200
O	-0.31942600	0.58872100	0.41484400
C	-4.22927900	-0.66821200	0.03364100
H	-4.28616100	-0.54830000	1.13204200
C	-3.19589200	-1.73578800	-0.32958200
H	-3.11183400	-1.76423900	-1.42475200
C	-1.83774500	-1.36675100	0.26271600
H	-1.90335600	-1.48564900	1.36230400
C	-1.52091700	0.07758000	-0.03782400
C	-2.63740000	1.09686500	0.01591300
H	-2.82123900	1.40035800	1.06560100
C	-2.37345100	2.37071700	-0.80239400
H	-2.02997500	2.10785900	-1.80842400
H	-3.32433800	2.90548800	-0.87679300
O	-3.67415700	-2.97904100	0.16971200
H	-3.00659900	-3.65460700	-0.01733500
O	-0.89297900	-2.30174100	-0.24950500
H	-0.02810200	-2.12138200	0.16063300
O	-3.83281300	0.56213300	-0.54781100
O	-1.38497200	3.19922900	-0.15091700
H	-1.59125300	4.12704800	-0.32264400
O	-5.48877600	-0.95293800	-0.49445400
H	3.79932200	2.92384700	-0.85346000
H	5.55800000	0.18430600	-0.46760900
H	-5.61787000	-1.91185700	-0.43223000
O	-0.69582100	2.79249400	2.71403600
H	-0.91816900	2.91915200	1.77744500
H	-0.32580700	1.90166000	2.75110500

TS Hydro -C1’ -1H2O, G= -861801.64 kcal/mol, i 590.02 cm -1

C	-0.91304900	0.76348400	-0.49755800
H	-0.50376000	0.72506000	0.52962800
C	-2.13799300	1.69396700	-0.54314800
H	-2.53658300	1.67751400	-1.56732800
C	-3.20735400	1.20692400	0.42728100
H	-2.81414800	1.27626600	1.45342000
C	-3.55691600	-0.24788000	0.12869500
H	-4.00648700	-0.29939800	-0.87448000
C	-2.29215100	-1.12070800	0.13622000
H	-1.84255200	-1.10222600	1.14134600

C	-2.57302200	-2.58031900	-0.24018300
H	-3.16514100	-2.61358500	-1.16778900
H	-1.62214100	-3.08525300	-0.43249800
O	-1.79094500	3.02470000	-0.16596800
H	-1.08298900	3.30592300	-0.76297400
O	-4.41804800	1.95602800	0.32560000
O	-4.47218000	-0.76771700	1.09448600
O	-1.37113500	-0.58406400	-0.82470300
O	-3.20706900	-3.29270100	0.80997700
H	-3.98303900	-2.78157800	1.08926000
O	0.01568700	1.17461300	-1.39729000
C	3.51686500	-0.94345300	0.69886800
H	4.40902300	-0.93009800	0.04583400
C	2.38319600	-1.71041300	0.01357800
H	1.50969800	-1.69151400	0.68076900
C	1.99939700	-1.04251000	-1.33728400
H	2.85415800	-1.15992600	-2.02298400
C	1.76115300	0.39091400	-1.00427400
C	2.88381800	1.10333400	-0.28756300
H	3.81430900	1.08654800	-0.88963700
C	2.59309200	2.55610800	0.07523500
H	1.60593800	2.64158300	0.53955300
H	3.36577600	2.90409600	0.76859500
O	2.82933700	-3.04100800	-0.18937400
H	2.16429400	-3.49540200	-0.72895700
O	0.92775200	-1.73824500	-1.94061800
H	0.08052300	-1.40290400	-1.57590500
O	3.09957900	0.39689500	0.93253200
O	2.64880500	3.30334500	-1.15690600
H	2.38313500	4.21780100	-1.00118300
O	3.83162700	-1.46697300	1.95338400
H	-4.20536900	2.88946200	0.46575200
H	-5.21396700	-0.14848300	1.16058500
H	3.77528700	-2.43238500	1.88835400
O	2.01340200	1.19261100	-2.72386400
H	2.18100800	2.12714700	-2.45805800
H	1.05674200	1.15730700	-2.92955500

P-C1'-Hydro-1H2O, G= -861868.33 kcal/mol, 15.04 cm -1

C	1.85942900	-1.11447900	-0.49361800
H	1.57273400	-1.48746400	0.50514400
C	3.37926300	-1.17019000	-0.67032400
H	3.60798400	-0.82008600	-1.68678500
C	4.06089300	-0.26568900	0.34816700
H	3.87683300	-0.66506300	1.35795200

C	3.48325500	1.14269400	0.26234900
H	3.71915000	1.55824900	-0.72883600
C	1.95486600	1.10782400	0.42134300
H	1.69921900	0.71221800	1.41677700
C	1.30929800	2.49033100	0.27096700
H	1.67178000	2.96554200	-0.65351800
H	0.22636600	2.36652600	0.18281500
O	3.87983400	-2.48757100	-0.46772300
H	3.45882200	-3.06692500	-1.11894100
O	5.46442400	-0.15796200	0.12381300
O	4.02422800	1.98509000	1.28099900
O	1.41275800	0.25403200	-0.60031300
O	1.53159400	3.31107300	1.40477900
H	2.48586100	3.32449100	1.57992200
O	1.29116000	-1.86475600	-1.50524100
C	-4.19881500	0.82099900	0.04698000
H	-4.89106100	0.22224900	-0.57463400
C	-2.97046200	1.22108200	-0.76996900
H	-2.27572800	1.73738100	-0.09062500
C	-2.28964800	-0.02877700	-1.34388000
H	-2.96701800	-0.45264700	-2.10718800
C	-2.08882700	-1.03182100	-0.24505400
C	-3.21278800	-1.21291600	0.75719400
H	-4.00337100	-1.84883100	0.30578400
C	-2.77067000	-1.88137300	2.06243000
H	-1.99736800	-1.27159500	2.54323200
H	-3.63498100	-1.96684300	2.72916900
O	-3.40337400	2.09913700	-1.80045400
H	-2.65962000	2.22892600	-2.40806400
O	-1.11879900	0.32175600	-2.06972300
H	-0.34507100	0.38838600	-1.47837900
O	-3.76898900	0.03523700	1.14856700
O	-2.25992000	-3.18470700	1.71925300
H	-1.90944400	-3.62001200	2.50622600
O	-4.86184700	1.91990900	0.59435200
H	5.83781400	-1.05082200	0.12021400
H	4.98936700	1.91330900	1.24079400
H	-4.79197100	2.64815600	-0.04146500
O	-1.39379500	-2.16401400	-0.61124500
H	-1.48209400	-2.82182600	0.11134300
H	0.33884500	-1.99721900	-1.32695600

R-C5-Hydro-1H2O, G= -861865.93 kcal/mol, 21.33cm -1

C	0.84941500	0.44153700	-0.20256900
H	0.77646400	0.44670600	-1.29856700

C	1.75847200	1.56712500	0.29176300
H	1.76190000	1.55227100	1.39141700
C	3.17488700	1.34730200	-0.22791900
H	3.15980000	1.37831500	-1.32797500
C	3.68407900	-0.02302100	0.21607900
H	3.84180000	0.01374800	1.31044100
C	2.70001100	-1.11140900	-0.11174000
C	3.10888400	-2.55117600	-0.10702900
H	3.71839600	-2.77290700	0.78476500
H	2.21865700	-3.18458900	-0.06991200
O	1.30316200	2.81326700	-0.20403800
H	0.38575800	2.96666400	0.10135500
O	4.08295700	2.32085500	0.27321900
O	4.91819200	-0.35849200	-0.42372100
O	1.36898600	-0.86085700	0.19909800
O	3.82189800	-2.92122400	-1.29029000
H	4.57641300	-2.31671200	-1.36256100
O	-0.39209800	0.59720600	0.39370400
C	-4.36106200	-0.42819000	-0.09481600
H	-4.41139400	-0.43577400	1.01000300
C	-3.37597800	-1.49242500	-0.57953500
H	-3.33879000	-1.44153700	-1.67760100
C	-1.98814000	-1.20418700	-0.01568300
H	-2.02284200	-1.31234400	1.08003400
C	-1.57233900	0.23739200	-0.34072400
H	-1.37670900	0.32224000	-1.41844000
C	-2.67566600	1.23911400	0.05978100
H	-2.75710700	1.25799000	1.15820800
C	-2.45264600	2.67199000	-0.43430800
H	-2.15098000	2.66723400	-1.48990000
H	-3.41305200	3.19024500	-0.34970300
O	-3.87423400	-2.75638100	-0.15769900
H	-3.22034300	-3.42931000	-0.39652200
O	-1.11209500	-2.17245200	-0.57792300
H	-0.21211000	-2.03131100	-0.23764800
O	-3.91362600	0.84382200	-0.52968100
O	-1.45778900	3.32907600	0.36847200
H	-1.59669000	4.28276300	0.30869600
O	-5.63314000	-0.59494300	-0.64557600
H	3.73011600	3.19510100	0.05366400
H	5.56846200	0.31387200	-0.17596200
H	-5.82102600	-1.54585900	-0.65578300
O	1.81304100	-0.96962700	3.10629300
H	1.31094800	-0.98150400	2.27677800
H	1.16032000	-0.93811800	3.81596900

TS Hydro -C5 -1H2O, G=-861797.89 kcal/mol, i 843.16 cm -1

C	0.78897800	0.38619000	0.02789000
H	0.74991200	0.37273400	-1.07295800
C	1.72771200	1.50219000	0.48704200
H	1.67259200	1.58343900	1.58414400
C	3.15921500	1.19960400	0.04928800
H	3.15158600	0.88827400	-1.00534900
C	3.83952100	0.10367700	0.89069200
H	3.90100100	0.46920100	1.92505800
C	3.21704200	-1.25353000	0.88509500
C	3.32692800	-2.31832500	-0.13251600
H	4.29217600	-2.81988200	0.04638300
H	2.53677600	-3.05877800	0.00984100
O	1.37339900	2.73654500	-0.12028900
H	0.42317900	2.91089400	0.04147800
O	3.99795800	2.34229600	0.20581000
O	5.15680300	-0.13471500	0.36863400
O	1.18765000	-0.86793000	0.49186200
O	3.25140700	-1.87253400	-1.48552100
H	4.06729700	-1.38568600	-1.67229900
O	-0.48929000	0.74346500	0.53330300
C	-4.45202000	-0.25920300	0.01960300
H	-4.53371300	-0.15029500	1.11712700
C	-3.47491700	-1.38335900	-0.32288200
H	-3.39827000	-1.44079100	-1.41859200
C	-2.10366500	-1.06739100	0.26253700
H	-2.18459600	-1.04859900	1.36171000
C	-1.63977000	0.32373400	-0.19792000
H	-1.40633000	0.28279600	-1.27237600
C	-2.73418400	1.38428600	0.04162000
H	-2.84559700	1.53916100	1.12643100
C	-2.46297100	2.73739200	-0.62226100
H	-2.18413800	2.58788900	-1.67377700
H	-3.39894000	3.30497600	-0.58850400
O	-4.01552400	-2.59533600	0.19661400
H	-3.34900400	-3.28697100	0.07461800
O	-1.22768200	-2.10847600	-0.14636500
H	-0.30741900	-1.86318900	0.09256500
O	-3.96935500	0.95239300	-0.53256000
O	-1.42597800	3.44533600	0.07736300
H	-1.49119900	4.38146600	-0.15008000
O	-5.71422300	-0.46325200	-0.54621200
H	3.47256200	3.11549700	-0.05549600
H	5.55367000	0.74207300	0.24040500

H	-5.90733900	-1.41078400	-0.48064300
O	2.17971800	-1.39112800	2.51261700
H	1.41364500	-1.12766500	1.80013600
H	2.13583600	-2.33250000	2.73891000

P-C5-Hydro-1H2O, G= -861863.80 kcal/mol, 7.05 cm -1

C	0.44852000	0.03674500	-0.49188300
H	0.28292800	0.17237200	-1.56764400
C	1.55592900	0.97672400	0.00661500
H	1.63448200	0.84259800	1.09578300
C	2.94304200	0.71036400	-0.66175100
H	2.83336400	0.00403100	-1.49437700
C	3.98141600	0.13031000	0.34386100
H	3.91384600	0.74038100	1.26367200
C	3.78489200	-1.31458600	0.70825600
C	4.85009200	-2.33974800	0.54265900
H	4.43619200	-3.34450100	0.68642700
H	5.25829900	-2.28041600	-0.47112300
O	1.19184500	2.31727500	-0.28405300
H	0.30200300	2.51466100	0.07908800
O	3.51146500	1.91276400	-1.17230200
O	5.28821600	0.24142500	-0.21117000
O	0.75212600	-1.33143200	-0.31226300
O	5.90972800	-2.22103100	1.51719500
H	6.37641800	-1.39536400	1.31782800
O	-0.70572100	0.41907600	0.22894900
C	-4.80659300	-0.06229800	0.12313000
H	-4.75026700	-0.13724100	1.22511800
C	-4.02560800	-1.20830400	-0.51908200
H	-4.08699600	-1.08641500	-1.61055200
C	-2.56385600	-1.14590500	-0.08922800
H	-2.50658800	-1.31430100	0.99826900
C	-1.98653800	0.24856000	-0.38367800
H	-1.89761900	0.37557700	-1.47269400
C	-2.90214300	1.35432200	0.18274100
H	-2.87027800	1.31152100	1.28305800
C	-2.53334700	2.77327900	-0.25795800
H	-2.34388200	2.79694200	-1.33917200
H	-3.39546700	3.41210000	-0.04049700
O	-4.64908200	-2.42544100	-0.12238300
H	-4.09704900	-3.15477100	-0.44029000
O	-1.89269100	-2.18761400	-0.78405800
H	-0.94150200	-2.15748400	-0.56403900
O	-4.23677500	1.16795600	-0.28755700
O	-1.37856200	3.23277400	0.46437400

H	-1.34458900	4.19625800	0.40823700
O	-6.13836400	-0.02734000	-0.29818000
H	2.85967800	2.62242200	-1.03441600
H	5.31376800	1.09938500	-0.66654400
H	-6.44868900	-0.94431700	-0.34671000
O	2.79986300	-1.50826800	1.66942000
H	1.22196600	-1.46072900	0.53382500
H	2.96781400	-2.32887100	2.15561100

R-C1'-Hydro-2H2O, G= -909823.95 kcal/mol, 24.76 cm -1

C	-0.94681500	0.25881200	0.24195500
H	-0.74435600	0.39712000	1.31327900
C	-1.83120300	1.39240200	-0.30133800
H	-1.91729500	1.26352200	-1.39053700
C	-3.22385000	1.27749300	0.32203200
H	-3.13739400	1.46785500	1.40383000
C	-3.79524300	-0.11821600	0.11298700
H	-3.91118100	-0.29545300	-0.96743000
C	-2.83258400	-1.17094000	0.67967000
H	-2.71378900	-1.01895000	1.76282600
C	-3.30179800	-2.61088600	0.43864000
H	-3.56158100	-2.73808000	-0.62382400
H	-2.48045800	-3.29629400	0.66807100
O	-1.32674900	2.66615400	0.02961400
H	-0.69465300	2.99262200	-0.65874500
O	-4.13256800	2.20455600	-0.25716000
O	-5.05566700	-0.26989100	0.76467000
O	-1.55513800	-1.01686200	0.02306400
O	-4.37649800	-2.96729100	1.28864700
H	-5.06650600	-2.29076100	1.19965300
O	0.27305400	0.27293900	-0.47190200
C	4.11970400	-0.97403100	0.05381300
H	4.06536900	-1.04752000	-1.04886600
C	3.10625300	-1.92211800	0.70208400
H	3.11929700	-1.73617600	1.78471200
C	1.70857500	-1.63518300	0.15890900
H	1.67880100	-1.98848300	-0.89041000
C	1.44349000	-0.15358900	0.15205900
C	2.56805100	0.83072100	-0.06072800
H	2.66577800	1.01392700	-1.14915100
C	2.30045500	2.18603200	0.61349400
H	1.23004500	2.38736300	0.63888000
H	2.68016200	2.14181000	1.64079400
O	3.52119100	-3.25366200	0.41705300
H	2.87772400	-3.86123300	0.80882000

O	0.78493600	-2.39816300	0.93170700
H	-0.11214900	-2.22208100	0.59510200
O	3.80874300	0.34827900	0.45452200
O	2.87648200	3.28113900	-0.10181100
H	3.84046100	3.21002000	-0.07768400
O	5.42150600	-1.21488000	0.49568800
H	-3.73044500	3.08386200	-0.18980500
H	-5.61069200	0.48378000	0.51444600
H	5.51782800	-2.17394600	0.60095300
O	0.49307900	3.57692600	-1.81110700
H	1.36551000	3.73744100	-1.41025900
H	0.63443500	2.88507600	-2.48487700
O	0.73137200	1.11550300	-3.18908900
H	0.34084200	0.73553700	-3.98524800
H	0.40546500	0.60269900	-2.42920000

TS Hydro-C1'-2H2O, G= -909760.81 kcal/mol, i 652.48 cm -1

C	-0.89934400	0.66406400	-0.51411800
H	-0.40497900	0.57903200	0.47112600
C	-2.06178600	1.66548500	-0.42955200
H	-2.54221000	1.72090600	-1.41774400
C	-3.08472900	1.19847000	0.59860300
H	-2.61571500	1.20214900	1.59467900
C	-3.53882300	-0.22034100	0.26927700
H	-4.05759600	-0.20278900	-0.70149100
C	-2.33205400	-1.16647000	0.15500900
H	-1.81936500	-1.22100000	1.12820600
C	-2.72793700	-2.58744100	-0.26347500
H	-3.38048600	-2.54011500	-1.14893900
H	-1.82505100	-3.13798400	-0.54218400
O	-1.60029600	2.95167700	-0.02690400
H	-0.90292800	3.21660400	-0.64560900
O	-4.25406300	2.01694800	0.61580800
O	-4.41689100	-0.72809000	1.27487200
O	-1.43993300	-0.64733400	-0.84281600
O	-3.33602200	-3.30936600	0.79548800
H	-4.05884400	-2.76665800	1.14797100
O	-0.02397800	1.04654900	-1.48576200
C	3.46517500	-0.84092800	0.73977300
H	4.36904200	-0.75081200	0.10914700
C	2.40695000	-1.68070900	0.02318400
H	1.51433400	-1.71227600	0.66434900
C	2.01692100	-1.05044000	-1.34243900
H	2.87974100	-1.15710200	-2.02015800
C	1.71345300	0.37916500	-1.05507200

C	2.71481500	1.16666600	-0.24349600
H	3.67568400	1.25997700	-0.78835900
C	2.24203300	2.57076800	0.14485500
H	1.26757800	2.52035600	0.63348300
H	2.96500200	3.00594200	0.84067400
O	2.93992800	-2.98342000	-0.15404800
H	2.31102000	-3.48769200	-0.69241100
O	0.97551800	-1.80584100	-1.93559900
H	0.11381900	-1.49351900	-1.59269800
O	2.93696800	0.45832700	0.97452200
O	2.09742900	3.39570900	-1.03225000
H	2.84363100	4.00483000	-1.09037500
O	3.79141300	-1.35472800	1.99591600
H	-3.97924000	2.92867900	0.78746500
H	-5.11067800	-0.06798300	1.42012300
H	3.78638300	-2.32142100	1.92474600
O	2.13825600	1.21647300	-2.69822700
H	2.00485300	2.15228800	-2.42996000
H	1.42291300	1.00609500	-3.36456800
O	-0.02294600	0.67084400	-4.15369900
H	-0.49105100	1.30943200	-4.70485900
H	-0.41815200	0.70810900	-3.25257300

P-C1'-Hydro-2H2O, G= -909825.78 kcal/mol, 14.34 cm -1

C	-1.78635900	1.50948100	0.46846000
H	-1.88556800	1.82621900	1.51646900
C	-3.12097100	1.69476500	-0.27348000
H	-2.96700200	1.42314300	-1.32848700
C	-4.19592300	0.79705500	0.32464000
H	-4.39605200	1.12381500	1.35702400
C	-3.70851700	-0.64628300	0.35055000
H	-3.55397700	-0.98669500	-0.68441600
C	-2.36840900	-0.73620100	1.09771300
H	-2.50554900	-0.39899200	2.13673000
C	-1.79182900	-2.15617000	1.12906300
H	-1.76741000	-2.56507400	0.10728700
H	-0.76477000	-2.10501200	1.50126800
O	-3.58793600	3.03753600	-0.16379100
H	-2.86800200	3.62892400	-0.42642200
O	-5.40714000	0.81993000	-0.42870100
O	-4.65254800	-1.49681200	1.00326800
O	-1.41688500	0.11666500	0.43692400
O	-2.50119300	-3.00405400	2.01664100
H	-3.44410000	-2.94472300	1.79480700
O	-0.77511900	2.27374100	-0.08774300

C	3.80216800	-1.55282300	0.69206300
H	4.46289500	-1.89693800	-0.12614800
C	2.33619300	-1.78591600	0.32432400
H	1.72674800	-1.34036800	1.12486000
C	2.00524200	-1.10134900	-1.00706000
H	2.47032600	-1.69313500	-1.81423800
C	2.56089400	0.29072100	-1.01651500
C	3.86364500	0.58970400	-0.30567200
H	4.71248500	0.36215600	-0.98454500
C	3.98947600	2.05636600	0.12041200
H	3.15783600	2.31443200	0.78478900
H	4.94045200	2.19784000	0.64510500
O	2.13123300	-3.19289900	0.26478100
H	1.24162100	-3.35697700	-0.08042400
O	0.60990200	-1.16823400	-1.29533000
H	0.10655800	-0.61671800	-0.66598200
O	3.99655100	-0.16283000	0.89263600
O	3.95057100	2.85092600	-1.08267900
H	3.86760900	3.78433300	-0.85143800
O	4.15293200	-2.16962000	1.89414100
H	-5.71271500	1.73657500	-0.47975700
H	-5.51946600	-1.33640800	0.60199300
H	3.66235900	-3.00430400	1.94476300
O	2.32229400	0.99151200	-2.17760800
H	2.79580500	1.84735700	-2.10611300
H	0.47867000	1.19851900	-2.69552200
O	-0.40014400	1.62642100	-2.73507100
H	-0.37782200	2.23276000	-3.48490300
H	-0.68607400	2.07136500	-1.04929600

TSF1gly-C1, G=-861855.70 kcal/mol, i 551.85 cm -1

C	0.98926100	-0.22784100	-0.87829300
H	0.43104000	-1.87539100	3.27078800
C	1.87375300	-1.44729500	-0.68684100
H	2.14917500	-1.82243300	-1.68680000
C	3.17480200	-1.15100900	0.05967800
H	2.97023600	-1.15261400	1.13907100
C	3.70400100	0.22056400	-0.32780600
H	3.85717000	0.26409800	-1.41845200
C	2.67652700	1.28040700	0.07731100
H	2.47148400	1.20335500	1.15276600
C	3.11612400	2.71403700	-0.24654400
H	3.47564000	2.76250700	-1.28609900
H	2.24859200	3.37443500	-0.16077600
O	1.21149100	-2.49609300	0.02586600

H	0.37099900	-2.74466200	-0.42549200
O	4.19597000	-2.09997500	-0.24554900
O	4.92618600	0.51103200	0.34213200
O	1.44212600	1.05309800	-0.64771800
O	4.08401500	3.19265400	0.66889200
H	4.81879700	2.55995700	0.68570700
O	-0.22246800	-0.33713600	-1.25410900
C	-4.35953600	0.44361500	-0.14576900
H	-4.44635300	0.58731000	-1.23939800
C	-3.37684500	1.46979000	0.42790200
H	-3.29329200	1.30027000	1.51068200
C	-2.00734200	1.26792600	-0.22694900
H	-2.11784800	1.46407300	-1.30669700
C	-1.58477500	-0.16359300	-0.03961700
H	-1.10064000	-0.39376600	0.90968500
C	-2.62434300	-1.15351500	-0.51703900
H	-2.73682400	-1.05272100	-1.61234000
C	-2.35780400	-2.62225100	-0.17048500
H	-2.08806900	-2.71902000	0.88325900
H	-3.27797300	-3.18953600	-0.34690300
O	-3.91770400	2.76006800	0.16400400
H	-3.25500100	3.41674300	0.42279600
O	-1.12039800	2.21507900	0.35124000
H	-0.23969800	2.08266900	-0.03783700
O	-3.88705500	-0.86599900	0.11372200
O	-1.27115900	-3.19901500	-0.91076800
H	-1.45248600	-3.12270900	-1.85741100
O	-5.61743200	0.52200200	0.45855100
H	3.92455500	-2.96653000	0.08685100
H	5.49270900	-0.27201500	0.26884000
H	-5.81493700	1.46205300	0.58816200
O	0.38219700	-1.37436400	2.44828500
H	0.71413500	-1.95471300	1.73472800

F1gly-C1, G=-861882.57, 9.01kcal/mol cm -1

C	1.86596600	0.06939400	-1.42228600
H	0.42413200	0.52457700	3.33972600
C	2.04163200	-0.93440900	-0.26142100
H	2.29004900	-1.88848600	-0.75504800
C	3.18839100	-0.59189600	0.68191000
H	2.89294000	0.25051800	1.32569600
C	4.40685000	-0.18979200	-0.13467100
H	4.66928500	-1.01528300	-0.81610200
C	4.07506800	1.05510000	-0.95693500
H	3.82193800	1.88572600	-0.28767300

C	5.20950800	1.50429800	-1.88602500
H	5.58479800	0.63958100	-2.45494400
H	4.80675800	2.22796400	-2.59897400
O	0.86650800	-1.08133400	0.51171300
H	0.14263500	-1.46529300	-0.04403400
O	3.56474400	-1.70969100	1.47841100
O	5.51809300	0.12539500	0.69457200
O	2.92345300	0.80440400	-1.81283900
O	6.24302300	2.15839700	-1.17428300
H	6.54845800	1.56864100	-0.46794000
O	0.83492600	0.12839000	-2.06029400
C	-5.04879700	0.25033700	-0.34952900
H	-4.73447100	1.13285800	-0.93890600
C	-4.70702500	0.48500500	1.13186600
H	-5.02160400	-0.39990900	1.70302700
C	-3.18490500	0.66046900	1.29169900
H	-2.91161900	1.58680100	0.75237600
C	-2.53279800	-0.51238300	0.64019200
H	-2.19764600	-1.34116600	1.26003400
C	-2.93924400	-0.75089800	-0.77764600
H	-2.65672000	0.11395600	-1.40572200
C	-2.34177100	-2.01910500	-1.38401000
H	-2.62751700	-2.88990300	-0.78871400
H	-2.71609400	-2.15996200	-2.40363700
O	-5.43635200	1.63457600	1.54746600
H	-5.15352100	1.85332600	2.44711900
O	-2.90600500	0.80360600	2.67440700
H	-1.93685300	0.89449500	2.76665600
O	-4.37879200	-0.89663800	-0.84028700
O	-0.90921200	-1.97043200	-1.37524100
H	-0.58954500	-1.24413600	-1.94173100
O	-6.41386900	0.00281100	-0.53200700
H	2.78687300	-2.02849600	1.95740100
H	5.63193700	-0.59883900	1.32836500
H	-6.89396700	0.61439100	0.04668100
O	-0.04419000	0.78671500	2.53776500
H	0.23174200	0.16114300	1.83864600

TSF12-3-C1, G= -861838.56kcal/mol, i 432.47cm -1

C	0.84882000	-0.46437700	-1.24376000
H	2.89069300	-5.13521000	0.68671700
C	1.64547300	-1.58079800	-1.14678100
H	2.63332900	-1.59151100	-1.59143200
C	2.82685400	-1.05749600	0.71905600
H	2.06367200	-1.14975100	1.49648400

C	3.24689100	0.31357300	0.30455300
H	3.88199700	0.23109400	-0.59149800
C	2.07257700	1.24496700	-0.02562400
H	1.34811500	1.25250600	0.79764100
C	2.51263600	2.69050500	-0.30469000
H	3.36676000	2.68508600	-0.99888200
H	1.68806200	3.22037000	-0.78790800
O	1.11363200	-2.84599600	-1.02438000
H	0.16990300	-2.81214200	-0.74310500
O	3.90135800	-1.90485500	0.82630000
O	4.02095600	0.97749800	1.34145300
O	1.39577900	0.79593800	-1.25344500
O	2.80471400	3.40286800	0.88280000
H	3.49537700	2.90720500	1.35097800
O	-0.51320400	-0.56922700	-1.29012800
C	-4.07374300	0.88856300	0.27205900
H	-4.54666400	0.57114200	-0.67606100
C	-3.01277000	1.95299900	-0.00660500
H	-2.56129400	2.24043200	0.95430100
C	-1.92889400	1.38184600	-0.91782200
H	-2.37962700	1.12479400	-1.89038700
C	-1.36088300	0.08929000	-0.31480600
H	-0.78879300	0.31098900	0.59397800
C	-2.51217600	-0.88124700	0.02318200
H	-2.99923000	-1.17619600	-0.92348300
C	-2.09429100	-2.15125200	0.77710600
H	-1.38218200	-1.91668900	1.57239800
H	-2.99038100	-2.57749500	1.24059100
O	-3.67668300	3.06497300	-0.59493000
H	-3.00534500	3.71731600	-0.84239600
O	-0.96100200	2.40424000	-1.08825600
H	-0.16163800	2.01938300	-1.48865400
O	-3.45232400	-0.23433200	0.87611800
O	-1.44888700	-3.12403400	-0.05368700
H	-2.08293800	-3.47730100	-0.69224500
O	-5.03963600	1.32544100	1.17986200
H	3.58059100	-2.81388900	1.02738000
H	4.68740800	0.34857000	1.65243400
H	-5.23616300	2.25107800	0.96959700
O	2.54625300	-4.26220100	0.90883000
H	1.99790300	-3.96311800	0.15236800

F12-3-C1, G= -861855.57 kcal/mol, 11.99 cm -1

C	0.857494697102	-0.631177844256	-1.121543492710
H	2.719385149315	-5.066700665307	0.913174460383

C	1.511147457020	-1.798259285395	-1.190773521451
H	2.586333366520	-1.836911760985	-1.285167482153
C	3.302622221036	-0.945332635680	1.078035342075
H	2.537368891535	-1.123388011161	1.827098378283
C	3.464009418737	0.344971084938	0.375318018365
H	3.949373398834	0.173845774911	-0.595055080415
C	2.122520638876	1.077026911785	0.123185185402
H	1.439915689963	0.910274984017	0.965190300550
C	2.281724020408	2.597064790482	-0.070775540099
H	3.094534468241	2.796429213708	-0.783969176985
H	1.353308233836	2.993238012136	-0.484215376538
O	0.932420562588	-3.041137794527	-1.138057278236
H	-0.024512750974	-2.980091055941	-0.912383362401
O	4.138306006797	-1.961301137442	0.740202729953
O	4.319086134086	1.279290688114	1.123555873198
O	1.490687901406	0.591693622520	-1.109714315554
O	2.486367208623	3.268082194500	1.159641589102
H	3.313717958128	2.915921157097	1.525652693629
O	-0.508291963674	-0.609118122058	-1.288290610533
C	-4.026270891328	0.942067358766	0.305352632969
H	-4.551252404646	0.625055386004	-0.615224528542
C	-2.947200089399	1.969846854497	-0.044849413759
H	-2.453653901834	2.276619091048	0.888934465292
C	-1.913413129932	1.338766042992	-0.973569367187
H	-2.405983184108	1.055191610205	-1.918006305050
C	-1.357767265769	0.060226836194	-0.334896462237
H	-0.794941661752	0.300953238642	0.574378146977
C	-2.529441423933	-0.875962335946	0.024444877578
H	-3.054912542308	-1.145539860830	-0.909157345005
C	-2.133662053137	-2.170701491188	0.746198711952
H	-1.335199975137	-1.992378441546	1.470670060210
H	-3.011076362373	-2.537465481120	1.289427672133
O	-3.608364852395	3.078131134113	-0.643860551087
H	-2.934740570555	3.713158780661	-0.926966034222
O	-0.920777109440	2.318995261578	-1.226304570428
H	-0.144545390130	1.884494885655	-1.618222357288
O	-3.420249226678	-0.193111224869	0.903336548220
O	-1.635724553205	-3.180316171880	-0.142791702676
H	-2.350820914108	-3.488585850486	-0.715527130705
O	-4.935785055242	1.427860837010	1.246110648705
H	3.716233125277	-2.822558721265	0.966474514328
H	4.940911043594	0.751752634861	1.643052083575
H	-5.125877350718	2.349804152862	1.014822493698
O	2.491747202601	-4.130460400987	0.954806847573
H	1.889518828205	-3.945936246448	0.203053731153

TSF15-6-C1, G= - -861853.69 kcal/mol, i596.65 cm -1

C	0.80529300	-0.24778100	-1.21263700
H	2.50585800	-5.15176500	0.67295600
C	1.78791200	-1.39555400	-1.10318900
H	2.44129500	-1.40585700	-1.98314900
C	2.71414600	-1.18463100	0.14020400
H	2.12514300	-1.39463400	1.04839600
C	3.27351900	0.24354700	0.23024500
H	3.81493200	0.46020300	-0.70401100
C	2.21289000	1.29006200	0.42880300
H	1.49397900	1.08431400	1.22201800
C	2.63181000	2.72993900	0.32682800
H	3.27053900	2.87225100	-0.55701500
H	1.75847800	3.38045000	0.22720100
O	1.13805200	-2.66066900	-1.05351000
H	0.30674300	-2.57831100	-0.54260300
O	3.85563000	-2.01922300	0.08125600
O	4.18881200	0.34036200	1.33292300
O	1.22387600	0.95746000	-1.11057600
O	3.30169300	3.14436600	1.52131300
H	3.98776100	2.48664700	1.72001900
O	-0.48604500	-0.60539200	-0.94423300
C	-4.20245100	0.86869800	0.10327700
H	-4.58332200	0.32584600	-0.78181900
C	-3.21748300	1.95422600	-0.32717700
H	-2.85154500	2.45161300	0.58300700
C	-2.02177700	1.35000500	-1.06816600
H	-2.37248500	0.90297500	-2.01232300
C	-1.40600400	0.23371500	-0.21122100
H	-0.89219700	0.67586500	0.65116800
C	-2.49230200	-0.75156400	0.27383400
H	-2.90089200	-1.26762500	-0.61251000
C	-1.98073100	-1.81063100	1.26451400
H	-1.29095400	-1.36247800	1.98480500
H	-2.84466100	-2.19282600	1.81877800
O	-3.93258600	2.87594600	-1.14074800
H	-3.30559400	3.54095300	-1.46075200
O	-1.14552600	2.43484900	-1.33647900
H	-0.23844300	2.09991700	-1.45957700
O	-3.52608500	-0.04901000	0.94981800
O	-1.26265200	-2.88338100	0.64144700
H	-1.89238700	-3.47459200	0.20786900
O	-5.25862000	1.38144100	0.85686700
H	3.60725900	-2.96383400	0.18115000

H	4.74824000	-0.45345300	1.29267100
H	-5.50735800	2.23245800	0.46484800
O	2.85559000	-4.58727700	-0.02646900
H	2.09365200	-4.15407600	-0.46282400

F15-6-C1, G= -861875.07kcal/mol, 13.52 cm -1

C	0.64662200	-0.20438400	-0.98710500
H	2.77459200	-5.07807100	0.73086100
C	1.66902200	-1.34098700	-0.92242300
H	2.11252400	-1.41548500	-1.91799000
C	2.83374300	-1.10694900	0.08520700
H	2.46317200	-1.34146600	1.09497100
C	3.40593400	0.32570800	0.08437300
H	3.55499400	0.64689100	-0.95995700
C	2.55844200	1.32458400	0.80683100
H	1.90716800	0.97464600	1.60635000
C	2.99338600	2.75369600	0.83515300
H	3.40897600	3.05201800	-0.13848700
H	2.16083400	3.42235500	1.07110000
O	1.01573700	-2.58688700	-0.66405000
H	0.31057500	-2.46761600	0.00527700
O	3.91837000	-1.95336600	-0.25765600
O	4.69181700	0.32316800	0.74145100
O	0.67919400	0.68834900	-1.81360100
O	3.96555200	2.96552100	1.87787000
H	4.64594600	2.28124400	1.77035100
O	-0.26580900	-0.37134600	-0.01980500
C	-4.33904000	0.64034500	-0.20633400
H	-4.45407700	0.03943200	-1.12785100
C	-3.38974700	1.81097900	-0.46453500
H	-3.30204300	2.38415100	0.47034600
C	-1.99466900	1.31762200	-0.87118800
H	-2.07429900	0.79111000	-1.83375500
C	-1.51199400	0.34533300	0.21276000
H	-1.35654900	0.89975100	1.14456500
C	-2.55920100	-0.76883600	0.43885500
H	-2.67735900	-1.33035700	-0.50457700
C	-2.18083400	-1.76696200	1.54607500
H	-1.64935300	-1.26536300	2.35888600
H	-3.10793300	-2.18479300	1.95165100
O	-3.97274600	2.60906700	-1.48348200
H	-3.33705500	3.30414400	-1.71067400
O	-1.19988900	2.48090000	-1.00428300
H	-0.37212400	2.20094000	-1.43684100
O	-3.79723700	-0.18192700	0.81582900

O	-1.31252100	-2.80992700	1.08085100
H	-1.84356600	-3.50191200	0.66545300
O	-5.58229100	1.06322700	0.26423700
H	3.70801900	-2.89291600	-0.06821900
H	5.12736100	-0.50682100	0.47969400
H	-5.83611100	1.84601800	-0.24813100
O	2.92562300	-4.51892800	-0.04029200
H	2.07348500	-4.09952100	-0.27343600

TSF21-6-C2, G= -861853.33 kcal/mol, i 76.45 cm -1

C	-0.42366600	-0.66823600	-0.57417800
H	-0.36254500	-0.07107200	-1.47535600
C	-1.57839100	-1.28996000	-0.16172700
H	-7.24589500	-1.11578000	-0.32716000
C	-2.94671800	-0.84429900	-0.57600300
H	-2.96697600	-0.61821600	-1.64741100
C	-3.35024700	0.46545000	0.17829200
H	-3.42207500	0.23006000	1.25104400
C	-2.27374600	1.55876700	-0.01350300
H	-2.20918800	1.80243600	-1.09124900
C	-2.69171500	2.87721500	0.70516500
H	-2.98621400	2.63920400	1.73836200
H	-1.81902200	3.53386000	0.74184900
O	-1.55556100	-2.19148200	0.84546800
H	-0.63175700	-2.42393300	1.05201400
O	-3.90384500	-1.88938200	-0.35698000
O	-4.58139300	0.96151900	-0.31927200
O	-1.05424800	1.14204300	0.51003900
O	-3.70832500	3.57721600	0.01431000
H	-4.42506500	2.94174700	-0.14899900
O	0.70529100	-1.04974400	0.04558000
C	4.63407000	0.30676700	0.21056200
H	4.58334200	0.29332500	1.31531300
C	3.62140900	1.31357900	-0.34035800
H	3.70550800	1.30926500	-1.43770700
C	2.19869400	0.90965900	0.04958800
H	2.10499500	0.93847100	1.14672700
C	1.97023200	-0.53700300	-0.41373300
H	1.99207900	-0.58451800	-1.51059800
C	3.05836600	-1.46137400	0.16075400
H	3.00368000	-1.42796400	1.26244000
C	2.93922800	-2.92832200	-0.28111200
H	2.60326800	-2.99100000	-1.32041900
H	3.93713500	-3.37738900	-0.22237800
O	3.96707600	2.59129800	0.17574500

H	3.25269700	3.20145900	-0.06254200
O	1.31643200	1.84020200	-0.54379100
H	0.42082500	1.74692200	-0.12258500
O	4.32790100	-0.99005900	-0.28061700
O	1.98886900	-3.65913100	0.49770400
H	2.42625900	-3.99291000	1.29106900
O	5.93563800	0.57723200	-0.21784500
H	-3.57723900	-2.45123800	0.36317500
H	-5.32961800	0.39503200	-0.03466200
H	6.06017100	1.53748300	-0.17479200
O	-6.47172700	-0.99998600	0.23685800
H	-5.76564100	-1.57698600	-0.11140400

F21-6-C2, G= -861864.74 kcal/mol, 20.98 cm -1
C -0.400215735778 -0.861057482430 -0.586161164877
H -0.344381616773 -0.358827195325 -1.544330232798
C -1.580039700202 -1.153653028611 0.030457785113
H -7.228647150836 -1.116236372350 -0.537389013853
C -2.932577004661 -0.759757967095 -0.482654252274
H -2.893611763371 -0.594852381927 -1.563356882551
C -3.419086628694 0.564171176915 0.182536849219
H -3.568546455524 0.367945710652 1.254999335729
C -2.336152782935 1.650709123264 0.029391204396
H -2.215977890231 1.890783259053 -1.043245193482
C -2.785168961615 2.980074885808 0.723397007960
H -3.116090370676 2.746101551000 1.746026821252
H -1.919378293895 3.643608330509 0.783475379987
O -1.619932601344 -1.830090081619 1.203028140898
H -0.714333067184 -2.040723343775 1.485539948674
O -3.875361511920 -1.824402362258 -0.266951982951
O -4.613607904289 1.029070976970 -0.422414255350
O -1.159982285701 1.221328410472 0.615326725007
O -3.779683531033 3.656916118524 -0.015760396386
H -4.479761146839 3.010875806777 -0.210679721641
O 0.738996929557 -1.226148581596 0.033357007517
C 4.584559600786 0.335012206075 0.242318867087
H 4.501911119016 0.283684773307 1.344181094109
C 3.531142260845 1.291941712986 -0.313913762952
H 3.637681378029 1.309172235967 -1.409122286651
C 2.127717991424 0.798867781854 0.041252081896
H 2.011782060650 0.814170597480 1.136438231701
C 1.979470308038 -0.656322105805 -0.436054921283
H 1.984634546447 -0.685714115874 -1.533792245436
C 3.124764602799 -1.532860978586 0.105163064792
H 3.059696286842 -1.559234625959 1.206635358422

C 3.124955129557 -2.979371162001 -0.417392019759
H 2.799505383933 -3.007821116869 -1.461697955298
H 4.158521877135 -3.342595593233 -0.378105905525
O 3.787214866546 2.580008367378 0.227421623313
H 3.044676539168 3.149697491970 -0.024910781380
O 1.207678071918 1.683165693848 -0.564907882619
H 0.328443543165 1.620617321763 -0.104336171492
O 4.365927264592 -0.958221659120 -0.297980384641
O 2.239257082402 -3.830842657222 0.305464728672
H 2.688219534023 -4.148429484367 1.098986569611
O 5.880943503026 0.692399638900 -0.135702228088
H -3.621974704762 -2.284242984583 0.548794429612
H -5.374775061117 0.460786515706 -0.177414646626
H 5.941844419557 1.658222831862 -0.082044531470
O -6.496013344122 -0.951248280292 0.068290481014
H -5.756177785948 -1.529163958096 -0.199892916507

TSF23-4-C2, G= -861842.35 kcal/mol, i 423.60 cm -1

C	-0.61568900	-0.28466900	-0.37536900
H	-0.44878700	0.01615700	-1.42131800
C	-1.57770800	-1.43493700	-0.27802000
H	-6.98825700	-1.43741700	-0.05551900
C	-2.84408000	-1.34160200	-0.79143500
H	-3.08159300	-0.77684900	-1.68401200
C	-3.47277400	0.57439100	0.32058100
H	-3.45500300	0.19628000	1.34557500
C	-2.32119500	1.44778200	-0.10788600
H	-2.31729500	1.53930200	-1.20851300
C	-2.40581600	2.88102400	0.46398400
H	-2.48753000	2.82809900	1.56000200
H	-1.48558200	3.42078600	0.21605900
O	-1.36492400	-2.38787900	0.69137900
H	-0.40597200	-2.57808500	0.74215400
O	-3.76425600	-2.33934800	-0.51802600
O	-4.68389200	1.04102600	-0.13855500
O	-1.07222700	0.89715300	0.34314400
O	-3.47214700	3.61759000	-0.10823200
H	-4.27626800	3.07639400	-0.05918300
O	0.56210200	-0.68529000	0.24824500
C	4.60505000	0.23472800	0.16824400
H	4.62867100	0.20234300	1.27365900
C	3.67512400	1.36443500	-0.28980500
H	3.68668100	1.38638700	-1.38970300
C	2.25125100	1.09679500	0.19136000

H	2.23667700	1.10397500	1.29255500
C	1.82350800	-0.29439800	-0.28686700
H	1.78319000	-0.31035600	-1.38563000
C	2.84209400	-1.34327200	0.19479800
H	2.86239000	-1.33081900	1.29849700
C	2.52524600	-2.77479000	-0.26272100
H	2.15990000	-2.77912000	-1.29330500
H	3.45308700	-3.35654200	-0.22958600
O	4.20360400	2.57701400	0.23435000
H	3.56926900	3.28324300	0.04320000
O	1.42673300	2.13877600	-0.31119500
H	0.52503500	2.00223200	0.03737100
O	4.12929400	-1.00903500	-0.31964100
O	1.50157600	-3.39740600	0.52509200
H	1.88849300	-3.72040100	1.34912300
O	5.89599300	0.37586100	-0.34666400
H	-3.34526500	-2.94356100	0.11975900
H	-5.38902400	0.39868200	0.10768900
H	6.13412000	1.31256100	-0.27375100
O	-6.16469300	-1.18240800	0.37705000
H	-5.45676300	-1.75460300	0.01675000

F23-4-C2, G=-861851.05 kcal/mol, 18.95cm -1

C	-0.78408400	-0.20185000	-0.11408400
H	-0.68934400	0.07075200	-1.17593400
C	-1.67164000	-1.41484400	0.02914800
H	-6.61391800	-1.75674700	-0.66640900
C	-2.58753100	-1.78657600	-0.87162500
H	-2.83854700	-1.21707500	-1.75505700
C	-3.63601100	1.13187300	0.30515700
H	-3.91390100	0.67395500	1.25166700
C	-2.30129700	1.76481100	0.12690300
H	-2.15164800	1.97660500	-0.94130200
C	-2.18818200	3.07709400	0.92632500
H	-2.29649200	2.85140400	1.99739500
H	-1.20690000	3.53362800	0.77107200
O	-1.50606000	-2.20278500	1.14795800
H	-0.54783200	-2.35646300	1.26468800
O	-3.37716300	-2.90274200	-0.68947400
O	-4.30852100	0.76139800	-0.81573400
O	-1.18113300	0.95989700	0.62785000
O	-3.13991400	4.03552000	0.49260900
H	-4.01510800	3.61994200	0.52999800
O	0.45036700	-0.58794100	0.43842900
C	4.49022800	0.21436500	-0.11246700

H	4.65504500	0.21850800	0.98142700
C	3.53567200	1.35664100	-0.48527400
H	3.41359800	1.35363700	-1.57859300
C	2.17758200	1.13318400	0.17277400
H	2.29968700	1.15887800	1.26711800
C	1.66515100	-0.25482400	-0.22234300
H	1.52236000	-0.29998100	-1.31187300
C	2.70592200	-1.31255600	0.18597400
H	2.85819000	-1.25429700	1.27766900
C	2.30280300	-2.75263500	-0.16156000
H	1.78616700	-2.78895500	-1.12459000
H	3.21641500	-3.35215200	-0.24415800
O	4.15310600	2.56663400	-0.06031900
H	3.51844400	3.28608100	-0.19090000
O	1.31131900	2.18134700	-0.23915500
H	0.45732500	2.04452800	0.21246500
O	3.93027200	-1.03217500	-0.49072300
O	1.39973900	-3.31787400	0.79714800
H	1.90511000	-3.63207900	1.55787900
O	5.70744400	0.30516200	-0.79266500
H	-3.03816600	-3.35661500	0.10314400
H	-4.98981100	0.08332900	-0.60241200
H	5.98211400	1.23430200	-0.76773700
O	-5.79321400	-1.46861000	-0.24968300
H	-5.12007800	-2.15579100	-0.42490500

TSF31-2-C3, G= -861842.77 kcal/mol, i 393.87 cm -1

C	-0.61142500	0.37469000	-0.07418700
H	-0.61471300	0.56785600	1.00486500
C	-1.75161500	2.09430500	-1.13340000
H	-1.30580400	1.70968300	-2.04564700
C	-2.99040500	1.68785700	-0.73364800
H	-4.42114900	-2.50118200	3.57775100
C	-3.47718900	0.30221700	-1.01666900
H	-3.26592300	0.03499400	-2.05789200
C	-2.73140300	-0.73004100	-0.11273700
H	-2.85088500	-0.45524100	0.94378100
C	-3.23019600	-2.16478700	-0.32221400
H	-3.19884100	-2.41825100	-1.39182200
H	-2.56975500	-2.85245700	0.21198000
O	-1.25347900	3.26110600	-0.61347700
H	-0.27795700	3.28297800	-0.70221400
O	-3.61732900	2.26294700	0.35129400
O	-4.89535400	0.18283700	-0.82908000

O	-1.33373300	-0.74732400	-0.46437100
O	-4.54188900	-2.35963500	0.20378600
H	-5.09485200	-1.64372300	-0.15934300
O	0.61188400	0.39402700	-0.68617800
C	4.60997600	-0.35387400	0.08670900
H	4.76432000	-0.39977300	-1.00779600
C	3.64236000	-1.46204900	0.51531300
H	3.50581300	-1.39148900	1.60434400
C	2.30041200	-1.25699500	-0.17737400
H	2.43787300	-1.37069900	-1.26406900
C	1.78981600	0.16073300	0.09882500
H	1.54896200	0.25567800	1.16601500
C	2.85434400	1.20831200	-0.27269600
H	3.02420700	1.17089200	-1.36323600
C	2.49712600	2.64956000	0.12558100
H	2.02739800	2.67117300	1.11222800
H	3.42801400	3.22439600	0.18094900
O	4.25001500	-2.70226700	0.17055500
H	3.61634100	-3.40910200	0.35999900
O	1.41029300	-2.25495000	0.30720300
H	0.54827100	-2.15062500	-0.12714700
O	4.06363500	0.91056000	0.42354400
O	1.56823100	3.28230700	-0.76389400
H	2.00155200	3.47567300	-1.60538100
O	5.83144600	-0.43096000	0.75828200
H	-3.14606700	3.07999400	0.57934800
H	-5.16077000	0.84548300	-0.17169100
H	6.07569900	-1.36779900	0.80641000
O	-4.07189500	-1.87351800	2.93482900
H	-4.38574800	-2.17111300	2.05816900

F31-2-C3, G= -861854.09 kcal/mol, 21.63cm -1

C	-0.64684000	-0.96977000	0.39787500
H	-1.03080300	-1.50335100	1.27415900
C	-1.73070900	2.59906400	-1.37476600
H	-1.36721300	2.21932000	-2.32417100
C	-2.69836900	1.99168300	-0.67680300
H	-4.28301200	-2.38624000	3.41349100
C	-3.38374700	0.71322500	-1.04969500
H	-3.19454800	0.49788100	-2.10630500
C	-2.89184100	-0.50015300	-0.22327000
H	-2.94502100	-0.27227700	0.84685800
C	-3.69632700	-1.77703200	-0.51484300
H	-3.79712800	-1.92117300	-1.59964200
H	-3.15694000	-2.63450600	-0.10552300

O	-1.19610700	3.77255600	-0.88719300
H	-0.21917500	3.70708300	-0.88422900
O	-3.15676200	2.49591200	0.52065800
O	-4.81214100	0.83497400	-0.89300200
O	-1.52719000	-0.73378700	-0.61697600
O	-4.97814600	-1.76411400	0.11512700
H	-5.41624600	-0.94745000	-0.18228200
O	0.55271000	-1.39794800	-0.11794800
C	4.43540100	0.02301000	-0.20252600
H	4.38523700	-0.14924500	-1.29362200
C	4.22011900	-1.29806300	0.54028300
H	4.26247300	-1.09070800	1.61940000
C	2.85045500	-1.86289700	0.18056700
H	2.83885200	-2.09871000	-0.89462800
C	1.74800100	-0.83603900	0.45288500
H	1.62387500	-0.70937300	1.53624800
C	2.10306400	0.51511700	-0.21008100
H	2.05666400	0.38897300	-1.30322600
C	1.18698200	1.65599500	0.20275800
H	0.14637200	1.38561700	0.03029500
H	1.32696200	1.86490000	1.27147300
O	5.27559900	-2.17269400	0.15869500
H	5.10347200	-3.04039000	0.55172400
O	2.67504200	-3.05700700	0.93736800
H	1.84935900	-3.47485400	0.65542600
O	3.41939100	0.92978700	0.17821300
O	1.43025200	2.83453300	-0.57512100
H	2.35625300	3.09348400	-0.46156200
O	5.64324800	0.63470200	0.14385900
H	-2.68426000	3.32864100	0.68678700
H	-4.96967200	1.50972500	-0.21408100
H	6.32035400	-0.05825000	0.17229100
O	-3.82960700	-1.96800600	2.67276000
H	-4.46474600	-1.95051400	1.93038600

TSF34-5-C3, G= - 861845.52 kcal/mol, i 496.05 cm -1

C	-0.69933600	0.59126800	-0.37368100
H	-0.74493600	0.84531200	0.69702000
C	-1.51731000	1.61010600	-1.20924300
H	-1.37995100	1.33545200	-2.26424000
C	-2.97116900	1.55990500	-0.83302200
H	-4.28884600	-2.22384600	2.16336900
C	-3.72609900	0.45194900	-1.10802700
H	-3.53091000	-0.13902500	-1.99446800
C	-2.42339000	-0.99250500	0.08270000

H	-2.49663500	-0.61358300	1.10468800
C	-2.96738300	-2.36307600	-0.18076400
H	-2.91369200	-2.59284300	-1.25559600
H	-2.40858600	-3.14798800	0.34984100
O	-1.06071600	2.92550500	-0.93924700
H	-0.08221300	2.96291300	-0.99342700
O	-3.37182200	2.36927100	0.20227500
O	-5.00408000	0.28347900	-0.62043300
O	-1.20445700	-0.74239900	-0.55278300
O	-4.31445100	-2.46209900	0.29936200
H	-4.81330000	-1.69706700	-0.03884200
O	0.60252300	0.64109500	-0.85782500
C	4.48516700	-0.39092600	0.13344000
H	4.67984400	-0.43173500	-0.95439300
C	3.43432500	-1.43363100	0.51453300
H	3.25719300	-1.35357100	1.59713500
C	2.13303600	-1.15146700	-0.22947800
H	2.30676600	-1.27627600	-1.31027600
C	1.69057100	0.29889100	0.01439500
H	1.37678200	0.41203600	1.06153500
C	2.84694200	1.27641100	-0.28444500
H	3.06415200	1.24914800	-1.36404800
C	2.58116100	2.73018900	0.11489700
H	2.12328500	2.77673200	1.11179300
H	3.55198100	3.23425800	0.15338300
O	3.96929200	-2.71280300	0.19354000
H	3.27557500	-3.37109000	0.34476400
O	1.18501200	-2.10519500	0.22622400
H	0.31865500	-1.89647400	-0.16788500
O	3.99781800	0.89676900	0.46916400
O	1.73090500	3.35959000	-0.85802200
H	1.84343400	4.31664800	-0.79562200
O	5.67305400	-0.54872500	0.85094800
H	-2.73523600	3.10348400	0.24902500
H	-5.15097300	0.92616800	0.09230200
H	5.85094200	-1.49983400	0.90819400
O	-4.10041600	-1.87448500	3.05629600
H	-4.46289000	-2.51352800	3.68054100

F34-5-C3, G= -861854.85 kcal/mol, 19.67cm -1

C	-0.70221300	0.42393500	-0.28527100
H	-0.74378700	0.69951600	0.77768400
C	-1.54657900	1.39868900	-1.14303100
H	-1.38142300	1.12349900	-2.19317500
C	-3.00682800	1.32409500	-0.81785000

H	-4.51716000	-1.76806400	2.19057800
C	-3.89468400	0.58489700	-1.49113400
H	-3.64085900	0.02968300	-2.38428800
C	-2.00974900	-1.40386800	0.56072500
H	-2.07541300	-0.85074500	1.49172500
C	-2.90607300	-2.53798900	0.22759100
H	-2.69967100	-2.88616000	-0.79221700
H	-2.75049700	-3.37858100	0.91269500
O	-1.13218600	2.73184000	-0.87152500
H	-0.15442000	2.79570400	-0.88723400
O	-3.40900400	1.96769700	0.33360200
O	-5.18996600	0.38972600	-1.07482300
O	-1.17983600	-0.92422800	-0.43249700
O	-4.31592500	-2.23496600	0.38677300
H	-4.56959800	-1.49231500	-0.18733400
O	0.59416000	0.50948500	-0.78838300
C	4.53518700	-0.37685700	0.15211700
H	4.75350200	-0.30149700	-0.92934900
C	3.54166300	-1.51092900	0.40503700
H	3.34687700	-1.55401500	1.48668200
C	2.23434500	-1.22805300	-0.32886000
H	2.42789400	-1.22641800	-1.41356000
C	1.70311800	0.16062000	0.05573400
H	1.38538100	0.15541900	1.10738800
C	2.80639000	1.22250100	-0.14371800
H	3.03891400	1.29325600	-1.21811000
C	2.45759000	2.62510400	0.35869100
H	1.95559100	2.57443900	1.33408300
H	3.40183000	3.16534200	0.48086300
O	4.15424700	-2.71707900	-0.03649600
H	3.50078100	-3.42751700	0.03846100
O	1.34268000	-2.28092500	0.00550300
H	0.46739600	-2.07970500	-0.36708100
O	3.96583800	0.84214800	0.59634100
O	1.62365800	3.28904000	-0.60408300
H	1.65544300	4.24032600	-0.44042000
O	5.71579400	-0.53479600	0.88157200
H	-2.81983700	2.73140700	0.45229400
H	-5.35122600	0.96387200	-0.30748100
H	5.95583500	-1.47303100	0.84186400
O	-4.33358500	-1.45188100	3.09707500
H	-4.93058900	-1.93581600	3.67912000

TSF42-3-C4, G= -861841.62 kcal/mol, i395.27 cm -1

C	0.60354700	-0.51363700	-0.09927900

H	0.47821500	-0.72893600	0.98070100
C	1.53865000	-1.50314600	-0.72002400
H	2.02197500	-1.24373800	-1.65903400
C	3.32807200	-0.98118700	0.64020900
H	2.74690200	-1.29133100	1.50488500
C	3.39435500	0.35207300	0.33373500
H	6.94598500	-0.78706600	-1.59707200
C	2.17886800	1.17753000	0.65911200
H	1.85542300	0.96840400	1.68804600
C	2.38895200	2.68535500	0.47561200
H	2.51549100	2.89818300	-0.59251700
H	1.50366500	3.21757200	0.83252900
O	1.22170700	-2.83087300	-0.58244900
H	0.30125800	-2.93441300	-0.25826900
O	4.30661600	-1.88453000	0.23414200
O	4.24566700	0.92378300	-0.56395300
O	1.07635600	0.82375900	-0.24100000
O	3.48768200	3.15695000	1.23915100
H	4.29259400	2.81384200	0.82448100
O	-0.65274000	-0.62050400	-0.72861700
C	-4.63886300	0.27727400	-0.25893500
H	-4.72014200	0.00217700	-1.32675400
C	-3.70006400	1.47283000	-0.09645700
H	-3.61912600	1.69480900	0.97778800
C	-2.32450400	1.11472700	-0.64488600
H	-2.41078500	0.94085300	-1.72943800
C	-1.80773100	-0.17926400	-0.00254700
H	-1.54198300	0.02469600	1.04459800
C	-2.86725300	-1.29972000	-0.05060100
H	-2.98065100	-1.63430800	-1.09364100
C	-2.55704700	-2.51203000	0.83213300
H	-2.32598200	-2.17701400	1.85180900
H	-3.46310700	-3.12548800	0.86648800
O	-4.28735200	2.57220500	-0.78562000
H	-3.65125700	3.30198000	-0.77842100
O	-1.46886700	2.22044700	-0.39577700
H	-0.54594400	1.94289700	-0.54647600
O	-4.11103100	-0.82101100	0.46448000
O	-1.46251000	-3.27718500	0.29733300
H	-1.51545000	-4.17355200	0.65186100
O	-5.90422600	0.51879000	0.28234000
H	3.88199900	-2.74637300	0.11147900
H	5.07272800	0.40074600	-0.68923600
H	-6.13517000	1.43676600	0.07342300
O	6.53265800	-0.56851900	-0.75299200

H	6.05289600	-1.35993300	-0.44959000

F42-3-C4, G= -861852.79 kcal/mol, 14.54 cm -1

C	0.65642500	-0.57562200	-0.54647200
H	0.68952800	-0.80779500	0.53776500
C	1.42307700	-1.54841700	-1.37105700
H	2.45260600	-1.36806000	-1.64779800
C	3.55255000	-0.19299400	1.58795400
H	2.84051000	-0.18544900	2.40507200
C	3.30061200	0.45692800	0.43950400
H	5.93043800	-2.20170900	-1.16741500
C	2.03562500	1.26418200	0.28679900
H	1.51041300	1.27491800	1.25122300
C	2.32973000	2.71276000	-0.14723700
H	2.73356000	2.70090200	-1.16561200
H	1.39537000	3.27943300	-0.14724800
O	1.02871600	-2.84641700	-1.36830500
H	0.07612900	-2.90831200	-1.13726100
O	4.69967100	-0.95950300	1.77475900
O	4.11308200	0.51984200	-0.64814300
O	1.14759900	0.75277100	-0.73527400
O	3.19965600	3.37481000	0.75957200
H	4.09368900	3.03038700	0.63089200
O	-0.69157600	-0.65768600	-0.97456900
C	-4.53220200	0.28679900	0.24883100
H	-4.83871500	0.18380700	-0.80854500
C	-3.50978000	1.41715100	0.38372600
H	-3.22336400	1.48981800	1.44339800
C	-2.27924100	1.08975800	-0.45163200
H	-2.56966000	1.04964800	-1.51369100
C	-1.72742200	-0.28785900	-0.06219900
H	-1.33306700	-0.24429500	0.96366800
C	-2.84563700	-1.34840000	-0.12697500
H	-3.16066300	-1.47077700	-1.17520000
C	-2.46622200	-2.72281400	0.42735200
H	-1.96725200	-2.61464700	1.39990300
H	-3.39773900	-3.27924500	0.57559200
O	-4.14889800	2.61783000	-0.03844000
H	-3.47817500	3.31511100	-0.07330800
O	-1.33353500	2.12986900	-0.24874500
H	-0.49450200	1.85899700	-0.66726500
O	-3.94587200	-0.92734500	0.68321300
O	-1.61732400	-3.41256900	-0.50355400
H	-1.60536600	-4.35081200	-0.27540700
O	-5.64913500	0.48430200	1.06618700

H	4.93935400	-0.96326100	2.70956900
H	4.85289300	-0.13053000	-0.62113100
H	-5.88002400	1.42416300	1.01289300
O	6.05405600	-1.43430500	-0.59507300
H	5.79976300	-1.69936400	0.30494800

TSF45-6-C4, G= -861851.16 kcal/mol, i50.31 cm -1

C	0.37830200	0.07209800	0.58330500
H	0.29613200	-0.73038300	1.34646300
C	1.61249700	-0.35403900	-0.34613000
H	1.59837000	0.38781200	-1.14735900
C	2.94189800	-0.34169900	0.45337700
H	2.70216500	-0.14816800	1.50636600
C	3.93502100	0.73054700	0.03772700
H	5.96913300	-1.57335600	2.80304700
C	3.93883800	1.44121300	-1.10509100
C	4.99235200	2.48459900	-1.39793100
H	5.97200300	1.99375000	-1.51574900
H	4.76344400	2.99117500	-2.33875400
O	1.38703400	-1.64175000	-0.84279500
H	0.51210100	-1.70409700	-1.29015800
O	3.58437100	-1.63189800	0.40851200
O	4.90519200	0.99796200	0.96320800
O	0.60922000	1.23328200	1.17611500
O	5.08009800	3.51664200	-0.41457000
H	5.25065100	3.08418700	0.43515500
O	-0.72542100	0.02884800	-0.30539700
C	-4.86315400	-0.01407800	-0.20541900
H	-4.84369500	0.58522400	-1.13463400
C	-4.20558300	0.77128400	0.93171400
H	-4.24198300	0.14724700	1.83704600
C	-2.74994400	1.06705200	0.58367600
H	-2.71979000	1.72875000	-0.29589500
C	-2.02516900	-0.23808200	0.22334600
H	-1.94390300	-0.86618200	1.12325100
C	-2.80912400	-1.00511700	-0.85983000
H	-2.79891000	-0.41494500	-1.79006500
C	-2.26697800	-2.40162800	-1.17376100
H	-2.04509800	-2.94478400	-0.24595100
H	-3.05315600	-2.93903000	-1.71318800
O	-4.96614700	1.95900900	1.11887800
H	-4.49943000	2.51079000	1.76341900
O	-2.18537400	1.72521700	1.71030800
H	-1.27049500	1.98561000	1.50497300
O	-4.14799700	-1.21944000	-0.41588500

O	-1.08490900	-2.30191000	-1.98997000
H	-0.96674200	-3.13455500	-2.46457900
O	-6.17018400	-0.39703200	0.10471100
H	3.04034600	-2.20822100	-0.15531800
H	4.99763300	0.28433400	1.63647200
H	-6.59371800	0.35553000	0.54499600
O	5.09877400	-1.20253700	2.61460400
H	4.68116000	-1.76540900	1.93280200
H	3.17318700	1.26836400	-1.85132400

F45-6-C4, G= -861856.03 kcal/mol, 20.46 cm -1

C	-0.56594300	-0.29731800	-0.36735400
H	-0.38418200	-0.72429100	-1.37318200
C	-1.57984200	-1.20052000	0.36973800
H	-1.48792000	-0.99201800	1.44356600
C	-3.00549600	-0.93755100	-0.09876600
H	-3.02847400	-1.04717600	-1.19437900
C	-3.48747700	0.46289900	0.21550300
H	-6.78356600	-1.40109400	-1.25250900
C	-2.80413300	1.33917400	1.01502100
C	-3.15205600	2.79220200	1.19746600
H	-3.86846500	2.86302300	2.03854600
H	-2.25135100	3.32923900	1.50929300
O	-1.31747600	-2.57295400	0.13029800
H	-0.38690400	-2.76154900	0.37609000
O	-3.89973100	-1.91392600	0.47670100
O	-4.58438900	0.89200100	-0.43408800
O	-0.98133600	0.99262000	-0.49527600
O	-3.64233500	3.47286400	0.05838500
H	-4.31992100	2.92382400	-0.36132600
O	0.62610100	-0.47065000	0.41049900
C	4.67003200	0.26778500	0.20282300
H	4.62871300	0.36506500	1.30367400
C	3.79912000	1.34294700	-0.44856400
H	3.84241300	1.19757700	-1.53808400
C	2.35790800	1.19080700	0.02188300
H	2.31809100	1.37268500	1.10847400
C	1.87455300	-0.24484100	-0.23213100
H	1.77572600	-0.39799900	-1.31756900
C	2.87083200	-1.27793000	0.33040400
H	2.85643500	-1.22384500	1.43061400
C	2.58554000	-2.72285200	-0.09098100
H	2.39850300	-2.76971800	-1.17164300
H	3.48494400	-3.30616100	0.13146000
O	4.35111400	2.60913700	-0.09891800

H	3.73531300	3.29060800	-0.40603300
O	1.58877300	2.16365000	-0.66675700
H	0.63600600	2.00782100	-0.49791700
O	4.18385800	-1.01123700	-0.16656000
O	1.45907100	-3.25506700	0.63238000
H	1.53642000	-4.21736200	0.64295600
O	5.99437600	0.31834900	-0.24235900
H	-3.38827100	-2.74182200	0.50917500
H	-5.07263700	0.15613400	-0.89294900
H	6.22943000	1.25427600	-0.33371300
O	-5.82833000	-1.29588400	-1.33878100
H	-5.39939300	-1.84754100	-0.65107400
H	-2.04670500	0.95028600	1.68209000

TSF54-3-C5, G= -861838.10 kcal/mol, i 354.99cm -1

C	-1.04071600	0.45816700	-0.26693500
H	-1.09327400	0.24034200	0.80528800
C	-1.93625300	1.65437700	-0.64122500
H	-1.84986000	1.77677700	-1.73697700
C	-3.37561500	1.43317100	-0.28065200
H	-3.62339100	1.17833800	0.74710500
C	-3.73843600	-0.64528500	-1.22154700
H	-3.63752400	-0.08638400	-2.14727100
C	-2.65832100	-1.28422400	-0.67344000
H	-1.10186100	-1.30401000	2.60147100
C	-2.71686800	-2.34876900	0.36650800
H	-3.62492100	-2.94574200	0.23836300
H	-1.84614900	-3.00272600	0.27600100
O	-1.48918500	2.83535800	0.02025700
H	-0.50972200	2.87601400	0.00339200
O	-4.31047400	2.28423000	-0.82558900
O	-5.00626100	-1.03461600	-0.87012700
O	-1.41502700	-0.74491600	-1.00946600
O	-2.66181800	-1.86266400	1.74259400
H	-3.48941300	-1.40247000	1.93807100
O	0.24701800	0.79306800	-0.65832200
C	4.19842600	-0.31224900	-0.08984300
H	4.41356900	0.20224000	-1.04499000
C	3.23650300	-1.47486400	-0.32975200
H	3.03535500	-1.95135500	0.64064400
C	1.92683100	-0.95363700	-0.91163300
H	2.12886300	-0.50741300	-1.89920800
C	1.35473300	0.14199400	-0.00117500
H	1.01715300	-0.29320500	0.94728200
C	2.42563100	1.21992500	0.27135500

H	2.66363500	1.73639500	-0.67259100
C	2.01425700	2.27691100	1.29807300
H	1.43419400	1.82162300	2.11073100
H	2.93458900	2.70375000	1.71139600
O	3.88448000	-2.39314500	-1.20616300
H	3.23492500	-3.06618200	-1.45632100
O	1.06932000	-2.07775800	-1.05625300
H	0.16929300	-1.75778500	-1.25402600
O	3.59888300	0.60369600	0.80646500
O	1.24669200	3.29669400	0.63611500
H	1.09655700	4.01933600	1.25943600
O	5.38717100	-0.72737600	0.51983000
H	-3.89100900	2.87980000	-1.46367800
H	-5.64984200	-0.44688100	-1.28599500
H	5.63849500	-1.56879200	0.10982700
O	-0.28173200	-0.85020000	2.87759000
H	0.05706400	-1.33717000	3.63797900

F54-3-C4, G= -861859.15 kcal/mol, 20.92 cm -1

C	-1.062742584073	0.547895052439	-0.321598400969
H	-1.115090471306	0.305608761897	0.740674810798
C	-1.886102853424	1.812399224559	-0.627815204295
H	-1.722620054587	2.046634643666	-1.696200191093
C	-3.361259480148	1.655899862574	-0.328278355735
H	-4.004008212603	1.100976554199	-1.002645939692
C	-3.477978498911	-1.726552369647	-1.563504257591
H	-3.405929205900	-1.355857675194	-2.582232057691
C	-2.573065504798	-1.365015549502	-0.639744736261
H	-1.393634915517	-0.403418030909	2.826542446780
C	-2.569826963777	-1.882380619083	0.768096507165
H	-3.295723225157	-2.697502241978	0.839012890430
H	-1.578899865437	-2.272638450908	1.023696638724
O	-1.433377120837	2.883095126602	0.185970808928
H	-0.453659837748	2.901764492950	0.240563249001
O	-3.982859207710	2.718939873024	0.252239361270
O	-4.500142545336	-2.594308844369	-1.275458475903
O	-1.510789602790	-0.585898849376	-1.106663749419
O	-2.869008071879	-0.888876913282	1.776966530469
H	-3.455340123344	-0.212310415718	1.396372032312
O	0.244527518110	0.824407710547	-0.712126040402
C	4.132863602540	-0.457267338374	-0.107258970514
H	4.398148933547	0.133150575819	-1.004197094403
C	3.144044648867	-1.560605070584	-0.485064325881
H	2.895407719707	-2.117563811897	0.430063512752
C	1.874514466595	-0.939749995730	-1.056582685329

H	2.124442404070	-0.419922710324	-1.995449059711
C	1.309432361153	0.092957310177	-0.074770999471
H	0.929972102320	-0.410513015060	0.822825170413
C	2.400885895441	1.102781786636	0.338221015325
H	2.689462432522	1.702011119674	-0.540257956979
C	1.980684886610	2.060641186414	1.454970272010
H	1.357919047097	1.544894618611	2.195201366836
H	2.897119086935	2.419807821300	1.934828264868
O	3.794496973448	-2.414627283041	-1.422032508817
H	3.139919887339	-3.050585399424	-1.744839772244
O	0.973547442050	-2.008338730244	-1.321392398104
H	0.100975171596	-1.629171659802	-1.530706595638
O	3.532701238910	0.394688544799	0.849506192274
O	1.261601294811	3.168326987937	0.876601843408
H	1.172624761416	3.856112662431	1.549072996418
O	5.284686348563	-0.967444384876	0.500404171511
H	-3.278768429474	3.276723286584	0.635235423827
H	-5.040060249191	-2.737140719564	-2.061063513751
H	5.528363379905	-1.772876309485	0.019553201075
O	-0.475451545081	-0.177394316680	3.075956964441
H	-0.351549034534	-0.498346497843	3.976952618843

TSF51-6-C5, G= -861856.18 kcal/mol, i 345.22cm -1

C	-0.74458400	0.54816400	-0.03744100
H	-0.78063000	0.14176000	0.97250200
C	-1.80807800	1.47238700	-0.52866200
H	-1.71484700	1.58343400	-1.61823000
C	-3.21005600	0.97216600	-0.16984000
H	-3.24353700	0.75981000	0.90829300
C	-3.63233100	-0.29570800	-0.94632200
H	-3.66255500	-0.02800300	-2.01459000
C	-2.63663400	-1.41496700	-0.73943100
H	-2.44468400	-1.48042800	2.36318200
C	-3.02942300	-2.67225200	-0.00556900
H	-3.74134700	-3.25986200	-0.60018900
H	-2.13505700	-3.27292700	0.17036300
O	-1.67141300	2.76476700	0.09781400
H	-0.72862500	3.01176000	0.08716200
O	-4.19437800	1.95229900	-0.49891800
O	-4.93136800	-0.71537800	-0.51720600
O	-1.42828200	-1.13761100	-1.02573800
O	-3.61366600	-2.39983100	1.28946700
H	-4.42534500	-1.89271800	1.11452400
O	0.45394800	0.81020500	-0.57707400
C	4.43242200	-0.18408000	-0.30792200

H	4.50275400	0.16134800	-1.35598500
C	3.47677100	-1.37529600	-0.21348400
H	3.41306000	-1.67233600	0.84371600
C	2.09005900	-0.96980400	-0.70344000
H	2.14985200	-0.71620900	-1.77383300
C	1.62795900	0.27962200	0.05781100
H	1.39388600	0.00983000	1.09515000
C	2.69178200	1.39355100	0.03007700
H	2.78839700	1.76592600	-1.00213800
C	2.38991000	2.57842600	0.95210400
H	2.13476400	2.21376400	1.95596800
H	3.30756100	3.17264700	1.02117200
O	4.03206400	-2.42985800	-0.99104100
H	3.38025500	-3.14538900	-1.02367000
O	1.23904900	-2.08656100	-0.50644900
H	0.32863900	-1.88241700	-0.79967000
O	3.93572000	0.87334200	0.49565300
O	1.31785300	3.36992200	0.41436500
H	1.34571600	4.23710100	0.83810100
O	5.70028100	-0.48369200	0.19593300
H	-3.88769100	2.80040300	-0.14416800
H	-5.50163000	0.06841300	-0.53266900
H	5.91632200	-1.38534500	-0.08679000
O	-1.78349600	-0.85799900	2.73351000
H	-1.63340100	-1.12999000	3.64619800

F51-6-C5, G= -861865.34kcal/mol, 25.24 cm -1

C	-0.89483000	0.73506700	-0.12931700
H	-0.93430100	0.54493400	0.93338200
C	-1.91320800	1.58301800	-0.78561000
H	-1.77789700	1.56076600	-1.87449300
C	-3.28953200	1.03797800	-0.39947100
H	-3.33468800	1.01650600	0.69900500
C	-3.53593300	-0.38527600	-0.91793200
H	-3.72760900	-0.34099500	-1.99651300
C	-2.34973800	-1.34937800	-0.72036700
H	-1.60250500	-0.23844000	2.83531100
C	-2.20372400	-2.06956100	0.62360000
H	-2.71032600	-3.04033600	0.51929600
H	-1.14539700	-2.26834000	0.80196000
O	-1.88176100	2.96350700	-0.34767400
H	-1.04462200	3.12507800	0.11789900
O	-4.35460100	1.83586100	-0.90248600
O	-4.66134000	-0.96339600	-0.23349900

O	-1.67146200	-1.70954700	-1.68078000
O	-2.71380500	-1.36062000	1.75308700
H	-3.66312800	-1.22092700	1.59590300
O	0.29932000	0.63509200	-0.74038400
C	4.24361500	-0.20859000	-0.27540100
H	4.30383000	0.38381900	-1.20747600
C	3.37230600	-1.44614700	-0.49273300
H	3.29161600	-1.97223400	0.46981800
C	1.98962600	-1.01072200	-0.95823800
H	2.08825300	-0.54754000	-1.95144100
C	1.39017200	0.03576400	-0.00853900
H	1.00255500	-0.47116000	0.88296900
C	2.39313700	1.14295000	0.39699400
H	2.50351000	1.82651300	-0.45975500
C	2.00070100	1.93045900	1.66378200
H	2.14019700	1.26346800	2.52209800
H	2.70005000	2.76517700	1.77134000
O	4.02053700	-2.26981400	-1.45546300
H	3.41952300	-2.99635900	-1.67576200
O	1.18700100	-2.18186600	-1.03748300
H	0.32380600	-1.99014200	-1.45031000
O	3.65677000	0.57492200	0.74489600
O	0.67434400	2.45600600	1.64183300
H	0.11792300	1.91637500	2.22818500
O	5.52344400	-0.53738200	0.17957100
H	-4.16604600	2.75454800	-0.65562100
H	-5.41115700	-0.36136300	-0.35472700
H	5.80515000	-1.32958700	-0.30278300
O	-0.92204800	0.38026600	3.17089900
H	-0.88942300	0.26272200	4.12793700

TSF51-6-C5-Frag2, G= -861847.53kcal/mol, i 465.05cm -1

C	-0.52017900	2.00774600	-0.95949300
H	-0.49082900	2.91508600	-0.34536100
C	-1.85231700	1.45198300	-1.40050900
H	-1.64609000	0.59877700	-2.05584600
C	-2.74004900	0.97284800	-0.21636200
H	-2.11024700	0.75041800	0.65028600
C	-3.55172600	-0.31177500	-0.58550400
H	-3.98321400	-0.18825800	-1.58749800
C	-2.62349600	-1.52344800	-0.62201800
H	-1.47234400	-0.72320300	2.59277700
C	-2.52005500	-2.44228700	0.59582500
H	-3.24063900	-3.26233000	0.45282100
H	-1.51680500	-2.87386100	0.60718000

O	-2.65410200	2.42381100	-2.09781200
H	-2.10837400	2.87186000	-2.75780000
O	-3.66224500	1.96592900	0.20505400
O	-4.58656600	-0.51235600	0.37574700
O	-1.94901300	-1.74185300	-1.61660300
O	-2.73460100	-1.79128700	1.84534600
H	-3.60143600	-1.34705200	1.77921900
O	0.54172800	1.34660700	-1.19742900
C	4.25596900	-0.19749600	-0.00127700
H	4.39692200	0.49777900	-0.85036400
C	3.33842100	-1.35137500	-0.41442700
H	3.17141300	-1.98080700	0.47079200
C	2.00041700	-0.78467500	-0.90198500
H	2.19210200	-0.26762000	-1.85530900
C	1.45187000	0.21494300	0.07890700
H	0.63080200	-0.13912900	0.69734000
C	2.43747700	1.16672600	0.72161000
H	2.66879300	1.96700300	0.00220100
C	1.93667600	1.76443100	2.05071100
H	2.02965300	0.98738800	2.81893000
H	2.59191100	2.59437000	2.33107600
O	4.00318900	-2.09121800	-1.43365900
H	3.39900400	-2.78235300	-1.74013200
O	1.14324700	-1.90709300	-1.11209000
H	0.34388600	-1.64164300	-1.59140500
O	3.65649200	0.48463100	1.07708600
O	0.60834900	2.25763100	1.97208300
H	0.00545200	1.55844800	2.28071600
O	5.49342400	-0.65559400	0.46514000
H	-3.78904100	2.56950100	-0.54920500
H	-4.90990400	0.38026300	0.59782100
H	5.75509400	-1.39793700	-0.10048500
O	-0.80136800	-0.07953800	2.90634000
H	-0.68698600	-0.24326600	3.85009800

F51-6-C5-Frag2, G= -861871.11 kcal/mol, 17.09 cm -1

C	-1.04908500	2.92136600	-0.81769800
H	-0.52108000	2.82650600	0.14739500
C	-1.76929500	1.68515500	-1.35176600
H	-1.00018800	0.92248600	-1.55226200
C	-2.77252800	1.11961300	-0.33210700
H	-2.32179600	1.08345200	0.66507200
C	-3.29551200	-0.30123700	-0.72011500
H	-3.54493800	-0.29809300	-1.78927400
C	-2.25407400	-1.39598100	-0.50559500

H	-1.64143200	-0.64242800	2.85859600
C	-2.34032100	-2.29429500	0.72746200
H	-2.99277700	-3.14332100	0.47093800
H	-1.34334900	-2.68778900	0.93723700
O	-2.47696400	2.01134800	-2.53334100
H	-2.16677000	2.90037400	-2.79490200
O	-3.90657900	1.97430600	-0.23914000
O	-4.45761200	-0.59668500	0.05607500
O	-1.37210500	-1.56025600	-1.33585400
O	-2.80579200	-1.63154300	1.89826100
H	-3.67363400	-1.24694100	1.67160700
O	-1.04695100	3.94560200	-1.47556700
C	4.19531600	-0.29987600	-0.26558000
H	4.06099200	0.32913300	-1.16707000
C	3.45419500	-1.63217100	-0.46668000
H	3.59148200	-2.24222600	0.43712000
C	1.95185700	-1.35785400	-0.66024200
H	1.85476700	-0.80136500	-1.61044100
C	1.48897800	-0.51027000	0.47858900
H	0.94299100	-0.97098400	1.29834900
C	2.28797900	0.73407100	0.71168300
H	2.19925600	1.41395400	-0.15493700
C	1.89637900	1.50028700	1.97745100
H	2.08985700	0.86914400	2.85337100
H	2.51899700	2.39582100	2.06016300
O	4.03963900	-2.26932900	-1.59686500
H	3.52315400	-3.06589600	-1.78596400
O	1.29748200	-2.61808700	-0.77370200
H	0.38918700	-2.45616600	-1.08146300
O	3.68651900	0.38144100	0.86064600
O	0.53254900	1.90730900	1.92468000
H	-0.01240500	1.24913800	2.39786400
O	5.55755900	-0.50125100	-0.01131500
H	-4.05632300	2.32275000	-1.13711200
H	-4.94515300	0.24383900	0.13014000
H	5.86153700	-1.19263400	-0.61863600
O	-0.94495900	-0.07307800	3.24843200
H	-1.09202000	-0.05426300	4.20123200

TSF1'gly-C1', G=-861845.90 kcal/mol, i 442.45cm -1

C	-1.09591200	0.39034300	0.24691000
H	-0.61021700	0.42297800	1.22175900
C	-1.87712500	1.57274900	-0.24429700
H	-1.74815200	1.63087700	-1.33774300
C	-3.36711900	1.36233200	0.04297800

H	-3.53002300	1.37341100	1.13130200
C	-3.79550700	0.01224200	-0.52070300
H	-3.63560600	0.02061400	-1.60988500
C	-2.95859500	-1.11802100	0.09206800
H	-3.13208900	-1.16078600	1.17549500
C	-3.25799500	-2.49324900	-0.51200200
H	-3.22900000	-2.42612200	-1.61055300
H	-2.48006400	-3.19395100	-0.19520000
O	-1.47705100	2.77707500	0.37777300
H	-0.52188400	2.92064200	0.21241400
O	-4.18018600	2.35388900	-0.57778800
O	-5.16742900	-0.26340400	-0.23738900
O	-1.54673300	-0.84797000	-0.14819400
O	-4.49074100	-3.01654000	-0.05006900
H	-5.17510300	-2.34372300	-0.19409300
O	0.45442500	0.60267100	-0.91230400
C	4.33749700	-0.34004200	-0.51109800
H	4.29563700	-0.11725000	-1.59432900
C	3.41393100	-1.50631600	-0.15700100
H	3.40235800	-1.57796400	0.93862200
C	2.00542500	-1.21043400	-0.66052500
H	1.94961600	-1.51693600	-1.71791000
C	1.63610500	0.26594300	-0.58988000
H	0.98675200	-1.21627700	3.44024500
C	2.66483000	1.33730700	-0.24920100
H	2.84909500	1.93661100	-1.15890900
C	2.22083600	2.30618400	0.85966500
H	1.75177800	1.75749500	1.68362000
H	3.12885100	2.79579700	1.22585700
O	3.96755600	-2.69079500	-0.73063000
H	3.44309500	-3.45119800	-0.44526900
O	1.09139400	-2.02636700	0.09885700
H	0.18107900	-1.85790900	-0.20992000
O	3.89586600	0.78581400	0.22087800
O	1.31364400	3.28147500	0.31407000
H	1.37732900	4.08742700	0.84197700
O	5.65478600	-0.57501400	-0.11350400
H	-3.85571300	3.22157500	-0.29655700
H	-5.68095200	0.51888200	-0.48858600
H	5.82777800	-1.52080300	-0.23997500
O	0.87453400	-0.72944800	2.61545100
H	0.98998500	-1.37192500	1.88818500

F1’gly -C1’, G = -861881.19 kcal/mol, 7.35 cm-1

C -2.486591569347 -0.288073825560 0.936746682396

H	-1.666258058790	-0.833507917853	1.395645017944
C	-2.469416012139	-0.003284681228	-0.541655370771
H	-2.587533441884	-0.938063454359	-1.120656915966
C	-3.626258081038	0.918260173900	-0.925003612307
H	-3.429926771887	1.919584100094	-0.514482056970
C	-4.920678962109	0.378962479561	-0.329373338435
H	-5.104469887349	-0.627977614826	-0.737303074832
C	-4.799751483245	0.279790073899	1.196164405717
H	-4.623295623729	1.279426473439	1.615248295908
C	-6.036664421256	-0.328052408083	1.865636469936
H	-6.312450716097	-1.261641832249	1.350806938618
H	-5.784134341839	-0.575966589671	2.899963441875
O	-1.260696427239	0.638538288988	-0.964885282734
H	-0.512318452590	0.015478958680	-0.959935591461
O	-3.813722201607	0.994963252645	-2.335452228931
O	-6.025797553142	1.228423036876	-0.630752390879
O	-3.682371959425	-0.569487635668	1.536251725421
O	-7.120604181375	0.584119478282	1.912757570176
H	-7.269228075492	0.919829705294	1.014989688099
O	1.316649146118	-0.546426217428	-0.799143858939
C	4.737260330040	0.426828269728	1.005087048665
H	5.137189791410	1.227393792553	0.353998578965
C	4.655253073960	-0.891392654111	0.220955829311
H	4.243561976185	-1.654909636859	0.897968889321
C	3.678681304885	-0.718577038833	-0.965283848794
H	4.157631375132	-0.045086818974	-1.695504991405
C	2.392834705629	-0.041544822108	-0.501074751215
H	5.527330878068	-4.558802601208	-0.996454689875
C	2.583508328393	1.195987283972	0.375500280298
H	3.064379007498	1.981476533583	-0.232151307071
C	1.306735026533	1.789135738879	0.979674679884
H	0.693914450881	0.997950103899	1.426957161800
H	1.623699740325	2.463920580222	1.781392407304
O	5.969206980697	-1.207775810086	-0.177828186968
H	6.006222576089	-2.130346841063	-0.511138030622
O	3.385548555852	-1.957003736529	-1.591958094218
H	2.415757935630	-2.008072832564	-1.673863758926
O	3.442045810504	0.794100610063	1.446332047991
O	0.595201683728	2.529454241341	0.007092061642
H	-0.183190309426	2.020140823467	-0.283353264760
O	5.515321293221	0.286726622245	2.151704868321
H	-3.005448143887	1.351730034992	-2.729620874510
H	-6.000040161373	1.417997731994	-1.580563098784
H	6.287457234737	-0.245489944918	1.901825292305
O	5.591455303166	-3.669310556803	-1.363538865923

H 4.691062327623 -3.382431917447 -1.603254896485

TSF1’2’-3’-C1’, G=-861841.16 kcal/mol, i 401.45cm -1

C	-0.70200000	0.78113200	-0.25939100
H	-0.19822400	0.89712500	0.71073300
C	-1.78966600	1.83754800	-0.46290900
H	-2.18631100	1.71224600	-1.48118100
C	-2.90956900	1.61394200	0.54780400
H	-2.51587700	1.79282200	1.56073500
C	-3.41255800	0.17576100	0.45933500
H	-3.84908000	0.01943900	-0.53903700
C	-2.25242100	-0.81655100	0.64265900
H	-1.81779600	-0.69286200	1.64600600
C	-2.67538600	-2.27997000	0.47249100
H	-3.23443900	-2.39016100	-0.47026400
H	-1.77761000	-2.90118600	0.40819000
O	-1.28796100	3.14991300	-0.27156700
H	-0.56231300	3.31517000	-0.90099600
O	-4.02769300	2.46472100	0.31049500
O	-4.39313000	-0.09180400	1.46166200
O	-1.25484500	-0.53577900	-0.35781400
O	-3.42228700	-2.75262800	1.58039500
H	-4.14457500	-2.12567200	1.74397700
O	0.20168400	0.92560200	-1.32411300
C	3.25832100	-0.35122800	1.08011000
H	4.19322100	-0.30542900	0.48933400
C	2.41321800	-1.49288700	0.57587000
H	1.39762900	-1.57268000	0.96944900
C	1.88620100	-0.73837500	-1.49778600
H	2.90459900	-1.00539700	-1.75913200
C	1.53598500	0.55913500	-1.18301600
H	1.35892200	-4.95471800	-1.31429600
C	2.47607600	1.39653200	-0.36684000
H	3.47784300	1.33998400	-0.82501500
C	2.07811900	2.86968000	-0.15362200
H	1.31143000	2.91363800	0.62166400
H	2.95272400	3.41712900	0.21631400
O	3.12811200	-2.65445000	0.48079600
H	2.53498300	-3.37564300	0.16836500
O	0.96768700	-1.62826500	-2.00693300
H	0.07436400	-1.31354400	-1.76551500
O	2.58355800	0.88381000	0.98745300
O	1.51089100	3.51752700	-1.29094500
H	2.13484500	3.51658800	-2.02808900
O	3.57549300	-0.48143000	2.45519600

H	-3.71119400	3.37957900	0.30854300
H	-5.05668300	0.61278400	1.42012400
H	3.76602800	-1.41561900	2.62380200
O	1.24631600	-4.14601600	-0.80085300
H	1.09295500	-3.41321800	-1.43086500

F1'2'-3'-C1', G=-861861.56 kcal/mol, 16.57 cm-1

C	-0.681383909488	0.604210258849	-0.321129407266
H	-0.135353335330	0.790478443034	0.612442669440
C	-1.735467900237	1.683914833279	-0.584475950492
H	-2.178456381992	1.484637074798	-1.571637681293
C	-2.822568737345	1.602711264479	0.483288081420
H	-2.382183326710	1.870433754329	1.456600198473
C	-3.371613880724	0.181491154146	0.565029792230
H	-3.863364864955	-0.059066200822	-0.389968009880
C	-2.229897208651	-0.823838887829	0.788664780762
H	-1.741975924989	-0.614711110790	1.753437638701
C	-2.703303058251	-2.281341305614	0.793260394835
H	-3.326188335660	-2.466550656867	-0.095785306090
H	-1.827728382371	-2.933152418808	0.732524439113
O	-1.183578769008	2.990263528383	-0.531436994429
H	-0.463195979002	3.069605619903	-1.181485586732
O	-3.921722088834	2.464832684601	0.201026907744
O	-4.306525620548	0.047980784926	1.635893733382
O	-1.281550247110	-0.684124888622	-0.281327111708
O	-3.387030151112	-2.618687256123	1.989038843307
H	-4.083623579797	-1.958445602972	2.131004061701
O	0.192562361689	0.628414712414	-1.426719198132
C	3.112487391937	-0.459603467667	1.463968551522
H	4.110513904730	-0.320206178891	1.026714348761
C	2.546838314334	-1.769168027685	1.023988879668
H	1.470728832774	-1.910016894720	0.959345959240
C	2.020030818310	-0.622319788932	-2.088989088085
H	3.066774306327	-0.890773825540	-2.142790771481
C	1.544358187201	0.329296475939	-1.275172286101
H	1.798835205424	-4.689329965089	-1.952033369376
C	2.408878508367	1.092210265526	-0.302448591827
H	3.449896653188	0.933788125426	-0.624954774590
C	2.112630959108	2.608347644084	-0.222604657772
H	1.294017787939	2.764935194350	0.480907218459
H	2.995217665019	3.120018774627	0.178674145739
O	3.347468888874	-2.542428234187	0.244605194881
H	2.801146002979	-3.170948864574	-0.277770585235
O	1.221411006696	-1.388264955041	-2.900811613718
H	0.306167974095	-1.077764925533	-2.775824993948

O	2.273570973537	0.650886728784	1.058140523186
O	1.683690532738	3.201841481762	-1.447679546559
H	2.389072150324	3.165711447325	-2.106317689986
O	3.255280696501	-0.339991313715	2.856410452486
H	-3.578307590152	3.364829071023	0.105859050264
H	-4.954393924595	0.763287359309	1.553113134184
H	2.608910461283	-0.920848920229	3.281985298133
O	1.655719466448	-3.861848194323	-1.477701501123
H	1.433560147049	-3.180800796728	-2.140414581811

TSF1’5’-6’-C1’, G=-861843.13kcal/mol, i255.26cm -1

C	-0.68480700	0.71807300	-0.20912100
H	-0.18082200	0.71146700	0.76727400
C	-1.75543400	1.80880700	-0.27683900
H	-2.11608700	1.85693500	-1.31570400
C	-2.91558200	1.44441700	0.64728400
H	-2.55625100	1.47327600	1.68754600
C	-3.42751800	0.04172400	0.34286700
H	-3.81604800	0.01905700	-0.68661200
C	-2.27632900	-0.96872600	0.44938100
H	-1.86825400	-0.96153700	1.47066200
C	-2.68424800	-2.40396500	0.09604300
H	-3.20607600	-2.40566100	-0.87396300
H	-1.78445000	-3.01849400	0.00061000
O	-1.25427700	3.05821600	0.15210800
H	-0.39630100	3.22643400	-0.28569900
O	-4.01573600	2.33713400	0.49488600
O	-4.45344000	-0.34319500	1.25617700
O	-1.25379100	-0.56303400	-0.48800600
O	-3.47188600	-2.99356700	1.11563500
H	-4.20777900	-2.39311000	1.31363300
O	0.25629100	1.00345900	-1.23186100
C	3.05704700	-0.21890000	1.17912900
H	4.06752700	-0.29939100	0.72813000
C	2.12645200	-1.24166100	0.48171000
H	1.15920500	-1.21310300	1.00520000
C	1.90570100	-0.86690600	-1.01485700
H	2.85719100	-1.03859100	-1.52936900
C	1.55952700	0.60775000	-1.11977700
H	1.13258100	-5.06846600	-0.95564100
C	2.56854600	1.52338000	-0.94327000
H	3.57603000	1.13333800	-1.06457300
C	2.56973900	3.02090300	-0.94989700
H	3.13861900	3.33634700	-0.06576200
H	3.12425900	3.34268100	-1.84519100

```
 O                  2.74448300   -2.50238600    0.63674100
 H                  2.12236500   -3.22292800    0.40433600
 O                  0.96052900   -1.71534200   -1.65895400
 H                  0.06365500   -1.45170900   -1.36818900
 O                  2.56330900    1.07759200    1.09331600
 O                  1.27387300    3.62417100   -0.95566800
 H                  1.39237700    4.57884600   -0.86728100
 O                  3.13974700   -0.53387600    2.55598200
 H                 -3.69668200    3.23538900    0.66335600
 H                 -5.10005300    0.37738400    1.29187800
 H                  3.23784900   -1.49907300    2.60521200
 O                  0.89897200   -4.23123200   -0.53739700
 H                  0.92163000   -3.53591600   -1.22593100
```

F1’5’-6’-C1’, G= -861860.80 kcal/mol, 23.19 cm -1

```
C -0.498563030287    0.751606140106   -0.258920410544
H 0.006890911172    0.792080696170    0.715110049554
C -1.587902530449    1.820941196528   -0.362886118195
H -1.980815806255    1.797007051870   -1.390733846569
C -2.710654802493    1.496844504228    0.616609542108
H -2.318715994597    1.571471175552    1.642735629155
C -3.216751361812    0.077919755314    0.381365473702
H -3.638749304692    0.017732236562   -0.633389811972
C -2.057952187876   -0.926112928898    0.485878260384
H -1.633345280200   -0.891932783316    1.500387162442
C -2.476733516679   -2.368819463806    0.178952154363
H -3.027627118349   -2.392715198765  -0.774607331998
H -1.579705266858   -2.984393469664    0.065957625086
O -1.083147451961    3.102209784724  -0.039384480259
H -0.291148566125    3.272053014796   -0.586948118012
O -3.825882353737    2.371931962204    0.463870059727
O -4.211258252320   -0.283338735348    1.339403752932
O -1.051586199040   -0.553325868203  -0.477815035027
O -3.232325665689   -2.939182198438    1.233586735834
H -3.956093321550   -2.329506583151    1.447956217519
O  0.408667081442    1.002280519609   -1.302848378061
C  2.783601849845   -0.407875589378    1.538144658795
H  3.671410735988    0.043644421306    1.029733193209
C  2.102120556114   -1.365013515297    0.450250987806
H  1.074782215299   -1.549596797364    0.783640976735
C  2.102374137319   -0.783417748028   -0.993086134532
H  3.119689775636   -0.930229495284   -1.365483758391
C  1.748942355743    0.684179747516   -1.183636660686
H  1.488298511845   -5.011489287022   -1.407374428466
C  2.688472740512    1.611986033941   -1.400652569821
```

H	3.716860957338	1.257336035941	-1.428253051416
C	2.602139301451	3.096250297128	-1.602100532263
H	3.188200916392	3.578972065409	-0.803694392619
H	3.094706685900	3.340524039949	-2.556119782408
O	2.862037420156	-2.552644141491	0.480741486444
H	2.316284383170	-3.306866609620	0.161937089449
O	1.263767492671	-1.610997382487	-1.813955549061
H	0.338340631739	-1.357975876116	-1.631378187719
O	1.938376167563	0.565177646773	1.840231141073
O	1.269032476574	3.619078316141	-1.609147405177
H	1.323494547699	4.565723303716	-1.793696584937
O	3.205600333517	-1.156299960670	2.644254792142
H	-3.505779392654	3.280190704133	0.561861421937
H	-4.863522203355	0.432290910208	1.369410226487
H	3.439802188931	-2.040841092661	2.312715878807
O	1.220806467714	-4.224090871343	-0.918715756350
H	1.224618765250	-3.467127963451	-1.540334191211

S5 Additional calculations

S5.1 Reaction profiles comparison

We provide below the comparison between the reaction profiles computed at the B3LYP/6-31++G(d,p) and wB97xD/6-311++G(2d,p) levels, for the reactions initiated at carbon atoms C1 and C5.

Table S3: Relative free energies (298 K, 1 atm) for the reaction initiated at carbon atom C1, computed at the B3LYP/6-31++G(d,p) and wB97xD/6-311++G(2d,p) levels; with respect to the pre-reacting complexes. Values in kcal/mol. For the fragmentation reaction, cleaved bond is indicated within brackets.

Species	B3LYP/6-31++G(d,p)	wB97xD/6-311++G(2d,p)
R	0.0	0.0
TS-Abs-C1	3.40	2.03
P1	-19.05	-21.09
TS F1(gly)	-7.35	-4.53
F1(gly)	-34.15	-31.43
TS F1(C2-C3)	9.75	11.41
F1(C2-C3)	-7.15	3.40
TS F1(C5-O6)	-5.35	-3.34
F1(C5-O6)	-26.65	-25.93

Table S4: Relative free energies (298 K, 1 atm) for the reaction initiated at carbon atom C5, computed at the B3LYP/6-31++G(d,p) and wB97xD/6-311++G(2d,p) levels; with respect to the pre-reacting complexes. Values in kcal/mol. For the fragmentation reaction, cleaved bond is indicated within brackets.

Species	B3LYP/6-31++G(d,p)	wB97xD/6-311++G(2d,p)
R	0.0	0.0
TS-Abs-C5	3.40	3.41
P5	-21.30	-21.42
TS F5(C1-O6)	-7.90	-4.20
F5(C1-O6)	-17.30	-18.72
TS F5(C4-C3)	9.90	12.08
F5(C4-C3)	-11.10	0.26

S5.2 wB97xD/6-311++G(2d,p) geometries

R-C1, G = -861817.1395 kcal/mol, 22.34 cm-1

C	0.86734300	-0.26739300	-0.10149500
H	0.75850300	-0.32066000	0.99647900
C	1.80954300	-1.36571200	-0.58121100
H	1.84251300	-1.33250600	-1.67649400
C	3.19911700	-1.12435100	-0.02272200
H	3.16142200	-1.23748500	1.07054600
C	3.64785300	0.29003600	-0.34295800
H	3.72169100	0.39690700	-1.43419100
C	2.61812200	1.29803600	0.16587900
H	2.52857800	1.21080300	1.25738400
C	2.97039500	2.73992300	-0.17902200
H	3.18430200	2.81268200	-1.25441500
H	2.10140100	3.36646800	0.03164500
O	1.37468200	-2.63215100	-0.13189000
H	0.47908800	-2.81569700	-0.47738000
O	4.15941000	-2.00324600	-0.56897000
O	4.89359100	0.57646400	0.25870300
O	1.36753400	1.00858600	-0.45152000
O	4.02939700	3.23311300	0.59975300
H	4.75705100	2.60395000	0.54355500
O	-0.34556800	-0.45705600	-0.72221900
C	-4.31139800	0.41317100	-0.17159300
H	-4.40617700	0.36732500	-1.27145000
C	-3.35981900	1.53913400	0.21051900
H	-3.26489400	1.53886200	1.30453900
C	-1.99662500	1.28563500	-0.40426700
H	-2.09189900	1.31871200	-1.49952300
C	-1.51916300	-0.10546000	-0.00720300
H	-1.31791500	-0.12408900	1.07080400
C	-2.58301900	-1.16040100	-0.32285900
H	-2.71259100	-1.20837400	-1.41752200
C	-2.22941300	-2.55506800	0.19324700
H	-1.77098400	-2.48873300	1.18075000
H	-3.15772100	-3.12568100	0.27988800
O	-3.93352900	2.74616800	-0.23923800
H	-3.29810100	3.45054100	-0.08364800
O	-1.13873900	2.30848000	0.04336200
H	-0.24075000	2.12225100	-0.26631400
O	-3.80074300	-0.80946800	0.29355600
O	-1.29267900	-3.24491000	-0.62616700

H	-1.68194100	-3.40820400	-1.48769700
O	-5.55635300	0.55875800	0.41738300
H	3.88526600	-2.90485600	-0.38233700
H	5.49985300	-0.13593700	0.03533300
H	-5.76345000	1.49866900	0.42350700
O	0.51733900	-1.98288700	2.44321600
H	0.90248000	-2.50328500	1.70340600

TS-Abs-C1, G = -861816.3011 kcal/mol, i300.88 cm-1

C	0.91051900	0.36249100	-0.11726300
H	0.79051600	0.36758200	-1.27796900
C	1.84887100	1.51982700	0.21802500
H	1.85477000	1.64091100	1.30909900
C	3.25550900	1.20952700	-0.26211900
H	3.25577400	1.20996900	-1.36071600
C	3.68362900	-0.16570600	0.21210800
H	3.69747200	-0.17663300	1.31122900
C	2.67794900	-1.21121500	-0.26418300
H	2.62075600	-1.20079000	-1.35961700
C	3.01105100	-2.62354100	0.20117500
H	3.18734000	-2.61377800	1.28596100
H	2.14783200	-3.26331100	0.00735100
O	1.43127800	2.69838800	-0.42404300
H	0.51757000	2.91673500	-0.16130000
O	4.20035600	2.13779900	0.22707000
O	4.95703800	-0.50599800	-0.29389400
O	1.40216500	-0.87969700	0.28752100
O	4.09481900	-3.17407100	-0.49983100
H	4.82473500	-2.54639500	-0.45811800
O	-0.29866200	0.59391900	0.47600500
C	-4.26599400	-0.38619800	0.11947400
H	-4.35846300	-0.14142300	1.19288300
C	-3.30921700	-1.55737000	-0.05428800
H	-3.21762200	-1.75596800	-1.13024600
C	-1.94224400	-1.19280200	0.49527400
H	-2.03045800	-1.02109200	1.57821200
C	-1.47381700	0.09925700	-0.16012500
H	-1.27061500	-0.07925800	-1.22103000
C	-2.54018400	1.19142300	-0.03452900
H	-2.65949000	1.43958200	1.03376200
C	-2.19419900	2.46672000	-0.80354800
H	-1.72093700	2.21727600	-1.75393500
H	-3.12770100	2.99613100	-1.01086700
O	-3.87514900	-2.66425500	0.61079900

H	-3.24052100	-3.38546800	0.57664800
O	-1.09332600	-2.28656500	0.24070400
H	-0.18197000	-2.03171600	0.43756400
O	-3.76253100	0.73272500	-0.56383100
O	-1.27874300	3.31349000	-0.12035200
H	-1.68818100	3.64532400	0.68118400
O	-5.51144200	-0.64167900	-0.42935700
H	3.95231400	3.01213700	-0.08438800
H	5.54885200	0.23286600	-0.12358200
H	-5.71311900	-1.56845800	-0.26540000
O	0.64763600	0.93549000	-2.64150400
H	0.92891200	1.82832300	-2.37155700

P-C1, G = -861839.4271 kcal/mol, 17.37 cm-1

C	0.89268900	-0.24717000	-0.36424700
H	0.30732700	-2.43845200	3.13397600
C	1.82708100	-1.39734200	-0.61824500
H	1.98954200	-1.50219500	-1.70421100
C	3.16840000	-1.14007800	0.04465700
H	3.03405500	-1.20895600	1.13267600
C	3.65419200	0.25547000	-0.29282900
H	3.78190800	0.33472600	-1.38214200
C	2.61718700	1.28487300	0.14648400
H	2.44853400	1.20409800	1.22566500
C	3.01141700	2.71754500	-0.19235700
H	3.30588600	2.77318100	-1.24971000
H	2.13797200	3.35827600	-0.05573200
O	1.32005800	-2.60674200	-0.09428500
H	0.45631200	-2.81014200	-0.50477500
O	4.16091400	-2.04744000	-0.38508200
O	4.87210800	0.54562200	0.36024800
O	1.39234900	1.01796200	-0.54620500
O	4.01511200	3.20716800	0.65767900
H	4.74193500	2.57464900	0.65231200
O	-0.32797800	-0.40313600	-0.91077800
C	-4.26407600	0.45425200	-0.14711600
H	-4.37834300	0.49264800	-1.24545000
C	-3.29297600	1.53506500	0.30231600
H	-3.16979000	1.44192300	1.38925000
C	-1.95066000	1.31928000	-0.36919900
H	-2.08023000	1.43145200	-1.45601400
C	-1.46578800	-0.09836900	-0.09094800
H	-1.18732100	-0.20525600	0.96176600
C	-2.55221600	-1.12166600	-0.44225800

H	-2.70459600	-1.09836800	-1.53569900
C	-2.22354300	-2.55089800	-0.01406800
H	-1.79831000	-2.54985600	0.98988600
H	-3.15860900	-3.11708900	-0.00201500
O	-3.86063600	2.78212700	-0.03137700
H	-3.20523400	3.46122500	0.15194300
O	-1.08263200	2.31455200	0.12102100
H	-0.19431700	2.14798800	-0.21992700
O	-3.75525900	-0.80115800	0.21801200
O	-1.26466800	-3.19465300	-0.84615500
H	-1.58806700	-3.21617600	-1.74931500
O	-5.49805500	0.56668500	0.47241900
H	3.86004600	-2.93816300	-0.18745500
H	5.46965600	-0.19274500	0.20867100
H	-5.68842000	1.50567500	0.56388200
O	0.11602500	-1.88183400	2.37780900
H	0.64202300	-2.23501500	1.64214500

TSF1gly-C1, G =-861822.8592 kcal/mol, i724.30 cm-1

C	0.96451700	-0.27779300	-0.90261800
H	0.66768500	-1.03558200	3.24359700
C	1.80375700	-1.51000100	-0.63184000
H	2.00530300	-1.97626600	-1.60560600
C	3.15273900	-1.22265400	0.01636300
H	3.02480400	-1.24777800	1.10401600
C	3.63884500	0.15960700	-0.35557300
H	3.73842000	0.24215600	-1.44824200
C	2.60685200	1.17154700	0.12265700
H	2.39965000	1.01693700	1.18645000
C	3.02394000	2.61995900	-0.10801600
H	3.37487200	2.73862800	-1.14254500
H	2.14545600	3.25516000	0.02022000
O	1.12842900	-2.44642000	0.19510900
H	0.30613300	-2.73768500	-0.25094200
O	4.14752900	-2.14748600	-0.38093200
O	4.87237700	0.44686400	0.26493700
O	1.39283700	0.97971000	-0.61525200
O	3.98146400	3.05173100	0.82260700
H	4.71917700	2.43340900	0.79244500
O	-0.22058900	-0.37873900	-1.31366000
C	-4.33056900	0.40637100	-0.14480600
H	-4.42871900	0.56694100	-1.23348200
C	-3.34498500	1.41920400	0.42642300
H	-3.24454700	1.22716800	1.50239800

C	-1.98955100	1.23133100	-0.24067100
H	-2.11236200	1.43886600	-1.31530000
C	-1.57560100	-0.19831400	-0.08075600
H	-1.08491500	-0.43473800	0.86121400
C	-2.62611300	-1.16140600	-0.56175700
H	-2.75192200	-1.02282600	-1.65036800
C	-2.34851900	-2.63100500	-0.28171600
H	-2.14005800	-2.77470100	0.77949900
H	-3.24499800	-3.20264100	-0.53512400
O	-3.88385500	2.70197800	0.19541300
H	-3.21594600	3.34849200	0.44100000
O	-1.10838500	2.16134300	0.33981200
H	-0.22268300	1.99604200	-0.00784800
O	-3.86296600	-0.89647500	0.08640800
O	-1.22484700	-3.13751400	-0.99231700
H	-1.21791300	-2.75491700	-1.87401600
O	-5.57084500	0.48248000	0.46996600
H	3.95209800	-3.00026100	0.01352900
H	5.44092900	-0.32051100	0.14736300
H	-5.74909500	1.41320100	0.63821600
O	0.72908900	-0.73725800	2.33522100
H	0.81408400	-1.53260900	1.78374800

F1gly-C1, G = -861849.7665 kcal/mol, 12.51cm-1

C	1.54160100	-0.07190000	-1.23757900
H	0.59011800	0.19739200	3.22366600
C	1.92411900	-1.17940400	-0.24115400
H	2.24399200	-2.01787100	-0.87734500
C	3.08778900	-0.82596500	0.66435900
H	2.73901900	-0.14153700	1.44844700
C	4.15449900	-0.12637700	-0.14629300
H	4.46427200	-0.77783600	-0.97746900
C	3.57157500	1.15806500	-0.71368200
H	3.24421200	1.80702200	0.10403400
C	4.54725500	1.93365000	-1.59323600
H	4.99840400	1.24937100	-2.32526200
H	3.98450800	2.68814600	-2.14390500
O	0.84517500	-1.56501700	0.56800800
H	0.12428000	-1.94124100	0.01408100
O	3.67113500	-1.97681000	1.23498800
O	5.26954400	0.21420800	0.64415100
O	2.42988600	0.86927700	-1.54269500
O	5.51586700	2.60895900	-0.83638100
H	5.95118600	1.96923700	-0.26337300

O	0.49176600	-0.10087100	-1.82488900
C	-4.70279900	0.40468000	-0.47035700
H	-4.28223200	1.14089700	-1.17948900
C	-4.30291600	0.79967100	0.95163000
H	-4.71738300	0.05356300	1.64170400
C	-2.77771100	0.78782300	1.08130300
H	-2.39496600	1.57579400	0.40930400
C	-2.30347300	-0.53622400	0.60338600
H	-2.05686000	-1.30439700	1.32950500
C	-2.78307200	-0.90974600	-0.75620800
H	-2.40020800	-0.18422000	-1.49443200
C	-2.37292400	-2.30572600	-1.19184200
H	-2.70996700	-3.04172500	-0.46082900
H	-2.83757100	-2.53734700	-2.15365000
O	-4.85626300	2.07339000	1.19732500
H	-4.52055600	2.37945900	2.04444500
O	-2.46181400	1.08895600	2.41742100
H	-1.49574600	1.04702800	2.50338800
O	-4.21172800	-0.86980600	-0.78601100
O	-0.95921500	-2.42077000	-1.27837100
H	-0.62199800	-1.67901300	-1.80442600
O	-6.08168400	0.32894500	-0.61190300
H	3.00459100	-2.43899600	1.74948200
H	5.52083800	-0.56432100	1.15064200
H	-6.45834800	1.06102900	-0.11351400
O	0.32296400	0.53819100	2.36759300
H	0.35802800	-0.22281600	1.76025900

TSF12-3-C1, G = -861806.9262 kcal/mol, i540.34 cm-1

C	0.89358800	-0.46289500	-1.38996300
H	2.38425900	-4.95868400	1.16208800
C	1.68190900	-1.55958900	-1.20742600
H	2.70522500	-1.55194900	-1.55834700
C	2.59834600	-0.97454000	0.74040900
H	1.73660400	-1.00246400	1.41083800
C	3.10015300	0.36332200	0.32114600
H	3.83413000	0.22410800	-0.48626800
C	2.00155700	1.28959300	-0.19228800
H	1.20792600	1.38427300	0.55586900
C	2.51628000	2.69170800	-0.51446200
H	3.43500900	2.61164200	-1.11151600
H	1.76765300	3.20290200	-1.12119000
O	1.17155500	-2.81890100	-1.09093600
H	0.20070100	-2.79941000	-0.96173800

O	3.62406500	-1.81447100	1.05712900
O	3.74453600	1.04790300	1.40461100
O	1.44350600	0.78418600	-1.43221900
O	2.70638400	3.47334600	0.63510700
H	3.30525300	2.98772100	1.21312300
O	-0.46107600	-0.55965800	-1.43417400
C	-3.87475400	0.79972600	0.44549900
H	-4.41442300	0.56522700	-0.48921900
C	-2.82439400	1.86520000	0.17174200
H	-2.29237000	2.06030700	1.11255100
C	-1.83627100	1.35185300	-0.85954600
H	-2.37357700	1.16157000	-1.80078700
C	-1.24717500	0.02529200	-0.38839000
H	-0.63374600	0.17955500	0.50536600
C	-2.38610400	-0.94451800	-0.04747400
H	-2.94301900	-1.15216800	-0.97740500
C	-1.93421500	-2.27266900	0.55278200
H	-1.13417200	-2.11897300	1.27896500
H	-2.78755700	-2.70930800	1.07770100
O	-3.50107800	3.02032300	-0.26662000
H	-2.84133900	3.67181800	-0.52117500
O	-0.87980100	2.36417600	-1.04639000
H	-0.14849300	2.00839600	-1.56786100
O	-3.24036900	-0.36461700	0.91067200
O	-1.42074900	-3.18133600	-0.40886800
H	-2.08864900	-3.35454700	-1.07614300
O	-4.76147300	1.17660600	1.43919900
H	3.24888500	-2.67921900	1.31240000
H	4.33642700	0.42135400	1.82907900
H	-4.92354000	2.12002700	1.33868700
O	2.09155900	-4.04893900	1.23328300
H	1.74383800	-3.79972900	0.35738000

F12-3-C1, G = -861814.9369 kcal/mol, 11.5cm-1

C	0.86040100	-0.65489800	-1.17471200
H	2.67314000	-5.03461300	0.81681100
C	1.49879200	-1.81697500	-1.23232800
H	2.57570500	-1.85600400	-1.29081000
C	3.25686300	-0.98205500	1.06317500
H	2.41065900	-1.24598100	1.68636500
C	3.38966200	0.34037900	0.42009400
H	3.90827100	0.22632200	-0.53903600
C	2.02805800	1.00407800	0.14060300
H	1.32685300	0.75652500	0.94464300

C	2.09927300	2.52926100	0.03985300
H	2.92194900	2.81715000	-0.62716600
H	1.16541500	2.88381200	-0.39414000
O	0.91033400	-3.04481300	-1.22060100
H	-0.03179700	-2.97243600	-0.96766400
O	4.10517100	-1.95797400	0.68409700
O	4.16984700	1.25746000	1.22731700
O	1.49668200	0.54935800	-1.12490600
O	2.21259600	3.13454900	1.30240000
H	3.03581900	2.80754700	1.68260000
O	-0.49204000	-0.61045200	-1.35343200
C	-3.88941100	0.97237200	0.36477000
H	-4.46517500	0.59626200	-0.49986200
C	-2.86191900	1.98795100	-0.11924800
H	-2.30129500	2.34220200	0.75609000
C	-1.89828200	1.32129800	-1.08446800
H	-2.45750300	0.97909900	-1.96791000
C	-1.29033600	0.09854300	-0.41172800
H	-0.69067400	0.41247500	0.44721800
C	-2.41136300	-0.81948300	0.08307000
H	-2.99893400	-1.14687600	-0.79130200
C	-1.91996500	-2.06333700	0.81885900
H	-1.01876900	-1.84777800	1.39505200
H	-2.70165500	-2.37751600	1.51478800
O	-3.57639800	3.04687000	-0.71512000
H	-2.94128600	3.67433600	-1.07086800
O	-0.94423900	2.28225500	-1.46380500
H	-0.18258200	1.83096400	-1.84668600
O	-3.23518200	-0.10929500	0.97959600
O	-1.57296000	-3.12436400	-0.06052600
H	-2.35664300	-3.41759400	-0.53059600
O	-4.73795400	1.50134100	1.32191000
H	3.69148700	-2.82333600	0.87621100
H	4.68508500	0.72675000	1.83891500
H	-4.92140400	2.41252500	1.07173200
O	2.44124800	-4.10739900	0.88641000
H	1.84872500	-3.91417800	0.13713200

TS-F1(5-6), G = -861821.6713 kcal/mol, i777.05cm-1

C	0.83378100	-0.24101400	-1.34190900
H	2.06486600	-4.85265100	0.91814800
C	1.81083300	-1.37752300	-1.20009300
H	2.53702900	-1.34439800	-2.01597200
C	2.60556700	-1.18849400	0.12136000

H	1.92636100	-1.40080100	0.96236700
C	3.15780700	0.22851500	0.28062400
H	3.77589800	0.45510000	-0.60020400
C	2.08850200	1.26800200	0.39361500
H	1.31703800	1.05748500	1.13181700
C	2.50502900	2.70243100	0.32364000
H	3.20950200	2.84051800	-0.50644200
H	1.63748400	3.33933700	0.14185400
O	1.17819000	-2.63447300	-1.24673000
H	0.34549700	-2.57879200	-0.74565600
O	3.72857200	-2.02851900	0.16609400
O	3.95958400	0.30393000	1.45122000
O	1.23718600	0.94818400	-1.23155700
O	3.06774700	3.12703900	1.55262500
H	3.72571200	2.47335200	1.81505100
O	-0.45489900	-0.59332500	-1.11828200
C	-4.04807900	0.84798500	0.22656100
H	-4.49758900	0.30670700	-0.62474100
C	-3.11216200	1.93070800	-0.28697700
H	-2.66259500	2.41878500	0.58812400
C	-1.99916600	1.32508800	-1.12999400
H	-2.44079700	0.86808600	-2.02750900
C	-1.31591500	0.22588500	-0.32564200
H	-0.75067200	0.67986700	0.49400400
C	-2.34509200	-0.75200800	0.25382800
H	-2.82308200	-1.27873400	-0.58838900
C	-1.72847200	-1.79048200	1.19292700
H	-0.95421000	-1.33209200	1.81092200
H	-2.51833100	-2.14817600	1.85822700
O	-3.89001600	2.84690300	-1.02190100
H	-3.29845300	3.50711300	-1.39425400
O	-1.15232300	2.38866300	-1.49587900
H	-0.24516400	2.06430700	-1.58679300
O	-3.30998800	-0.05997100	1.00691500
O	-1.10540000	-2.87414100	0.51817900
H	-1.78401000	-3.42562000	0.12250100
O	-5.03227800	1.35585800	1.05617900
H	3.45172000	-2.95350500	0.29952800
H	4.50905100	-0.48928800	1.45440300
H	-5.29269500	2.21281500	0.70330300
O	2.61102500	-4.54954400	0.19073300
H	2.00840900	-4.10557000	-0.43059600

P-F1(5-6), G = -861844.2658 kcal/mol, 28.38cm-1

C	0.67439400	-0.33584400	-1.03690000
H	2.74959700	-4.88863100	1.06675900
C	1.70740200	-1.44956300	-0.95655000
H	2.19444900	-1.50501600	-1.92976600
C	2.79866300	-1.16734700	0.10017600
H	2.38480000	-1.40942100	1.08927500
C	3.27156100	0.29077800	0.10716300
H	3.47012300	0.59587300	-0.93146700
C	2.29398200	1.23095200	0.72246000
H	1.73306700	0.88938700	1.58792500
C	2.47210900	2.69420700	0.53524100
H	2.80420900	2.90808000	-0.48925300
H	1.53737100	3.22988800	0.70840600
O	1.07512100	-2.69682100	-0.73001200
H	0.33960300	-2.58509300	-0.10234200
O	3.93628800	-1.94708900	-0.16816800
O	4.48155400	0.39406500	0.85498700
O	0.66712100	0.52252900	-1.88132400
O	3.40772200	3.21484800	1.47953200
H	4.17120500	2.62738300	1.48031300
O	-0.18769900	-0.48441200	-0.03710300
C	-4.15248700	0.78308300	-0.12543200
H	-4.29944200	0.29126700	-1.10353400
C	-3.13156200	1.90309300	-0.27035500
H	-2.99911800	2.36319900	0.71831700
C	-1.78951000	1.35550000	-0.74152900
H	-1.91993200	0.92679600	-1.74283000
C	-1.38055000	0.26302900	0.23976600
H	-1.20766000	0.72283800	1.21697000
C	-2.49421800	-0.78586200	0.36456200
H	-2.64236000	-1.24995800	-0.62499100
C	-2.17705600	-1.89486300	1.36958500
H	-1.64887800	-1.48829500	2.23300200
H	-3.12590000	-2.30845200	1.71961600
O	-3.65735400	2.83289700	-1.18555700
H	-2.97736600	3.49204800	-1.35487800
O	-0.90741900	2.44735400	-0.77804800
H	-0.14101300	2.15868900	-1.29225800
O	-3.67989300	-0.16660800	0.79664400
O	-1.34610500	-2.91412300	0.83257500
H	-1.87971300	-3.50025400	0.29206300
O	-5.35766400	1.23804400	0.37991000
H	3.76634700	-2.87809700	0.06251100
H	4.99003500	-0.40206600	0.65192300
H	-5.53788700	2.09191000	-0.02627900

O	2.98124700	-4.50432300	0.21980400
H	2.16745700	-4.10922200	-0.13658500

R-C5, G = -861815.5914 kcal/mol, 27.55cm-1

C	0.64824900	-0.65568000	0.01322700
H	0.61757600	-0.44512400	1.09389900
C	1.39698600	-1.95658600	-0.24733800
H	1.34079600	-2.15993100	-1.32506600
C	2.85025500	-1.80087200	0.15763100
H	2.89798300	-1.65179100	1.24606800
C	3.44610100	-0.58430700	-0.52210000
H	3.42272500	-0.74030900	-1.60923000
C	2.60891800	0.65126300	-0.19592900
H	2.61327200	0.82406400	0.88938600
C	3.11871200	1.91105800	-0.88275300
H	3.24136400	1.72828500	-1.95650400
H	2.38238400	2.70580700	-0.75381900
O	0.84911300	-3.00756000	0.50223500
H	-0.09936600	-3.08128600	0.29732900
O	3.63317200	-2.91639600	-0.20992700
O	4.77503300	-0.35121600	-0.09431600
O	1.28168200	0.42986700	-0.65167900
O	4.32684300	2.37231200	-0.30808000
H	4.94483700	1.62953400	-0.29088500
O	-0.62003800	-0.80738700	-0.49729200
C	-4.37604200	0.78598600	-0.15078500
H	-4.51599700	0.50022200	-1.20828300
C	-3.25138400	1.80543000	-0.04197400
H	-3.11735800	2.04059000	1.02231600
C	-1.96725300	1.20567900	-0.58164500
H	-2.09793700	0.99813300	-1.65354900
C	-1.68749500	-0.11554700	0.12686000
H	-1.44013800	0.08907900	1.17644400
C	-2.91373000	-1.03280400	0.07511100
H	-3.08630300	-1.33211800	-0.96887000
C	-2.77523100	-2.29491100	0.91709400
H	-2.33098200	-2.05413600	1.88942500
H	-3.78452000	-2.67854100	1.08260000
O	-3.64752800	2.95250300	-0.75950200
H	-2.90362200	3.56116000	-0.77586700
O	-0.95103700	2.16057800	-0.38724700
H	-0.10487400	1.77259700	-0.65318800
O	-4.03901100	-0.35557200	0.59290600
O	-1.98115400	-3.26037600	0.23355300

H	-2.21210500	-4.13007100	0.56411500
O	-5.56267700	1.25832300	0.38663100
H	3.22756600	-3.70095600	0.16925400
H	5.25648800	-1.18128600	-0.16087400
H	-5.62438400	2.19337100	0.16690300
O	3.52994000	2.96472700	2.33104300
H	3.91717300	2.88165400	1.42938500

TS-Abs-C5, G = -861814.9263kcal/mol, i363.79cm-1

C	0.89228200	0.42984200	-0.06610000
H	0.78049400	0.42797800	-1.15995700
C	1.81702400	1.55908500	0.36948800
H	1.85777800	1.55622600	1.46718400
C	3.21040400	1.31973100	-0.18555600
H	3.16703600	1.37435500	-1.28200100
C	3.68734000	-0.06456400	0.20811000
H	3.75092400	-0.12003200	1.30495400
C	2.66940700	-1.10410900	-0.24859100
H	2.56928900	-1.01104000	-1.40649400
C	3.06598900	-2.54744600	0.01361800
H	3.38558800	-2.66211800	1.05780500
H	2.19380500	-3.18235200	-0.14834800
O	1.36447000	2.79074300	-0.12396000
H	0.44163200	2.92844600	0.15220000
O	4.15225100	2.24599200	0.31249000
O	4.94103700	-0.37204900	-0.36167800
O	1.42369100	-0.83766100	0.33037100
O	4.06393500	-2.98032800	-0.88236000
H	4.81118300	-2.37624200	-0.79540600
O	-0.31638900	0.61002700	0.56061100
C	-4.27115600	-0.40549500	0.12860900
H	-4.37870900	-0.23525000	1.21440100
C	-3.29181600	-1.54493700	-0.11471400
H	-3.19413800	-1.67474100	-1.20076000
C	-1.93390000	-1.18973300	0.46115600
H	-2.02576200	-1.09106100	1.55266600
C	-1.48635900	0.15434600	-0.10005800
H	-1.29189100	0.04533500	-1.17417600
C	-2.57446100	1.21248900	0.10517700
H	-2.70164000	1.38703400	1.18337700
C	-2.26624300	2.54938900	-0.55683600
H	-1.80819800	2.39232500	-1.53987900
H	-3.22060700	3.06217900	-0.69573400
O	-3.83733300	-2.70336500	0.47620400

H	-3.18648500	-3.40672800	0.40073900
O	-1.06079200	-2.24481500	0.13502800
H	-0.15613700	-1.98670500	0.35913500
O	-3.78251900	0.76510600	-0.47281000
O	-1.40220000	3.31759900	0.27604000
H	-1.50996300	4.24353300	0.05190200
O	-5.50675900	-0.64581200	-0.45082200
H	3.84153300	3.12916700	0.09557600
H	5.51961600	0.38417900	-0.22599500
H	-5.69750300	-1.58276700	-0.34088200
O	2.54818900	-1.22951400	-2.84042300
H	3.13585400	-2.00301400	-2.78504000

P-C5, G = -861839.7534 kcal/mol, 31.28cm-1

C	-0.94010800	0.45576000	-0.21395200
H	-0.92293200	0.27396600	0.86646400
C	-1.87222500	1.59852500	-0.57090100
H	-1.87159100	1.72556600	-1.66241300
C	-3.26579600	1.24486600	-0.08947600
H	-3.22472300	1.10484900	1.00024100
C	-3.73171200	-0.06193700	-0.71846000
H	-3.95125200	0.15054500	-1.77894700
C	-2.68236400	-1.12049900	-0.61661600
H	-1.37487500	-2.06634800	2.01643800
C	-2.96156800	-2.51265400	-0.21097300
H	-3.88015000	-2.87632600	-0.67802700
H	-2.13683200	-3.16157200	-0.50901200
O	-1.48370100	2.77764800	0.08179500
H	-0.54969900	2.96259400	-0.11857000
O	-4.21704100	2.23228900	-0.41747800
O	-4.89860000	-0.55038600	-0.08240400
O	-1.38682000	-0.76541900	-0.84519800
O	-3.07132400	-2.65496300	1.21832300
H	-3.83534100	-2.13408000	1.48668600
O	0.31336800	0.74306100	-0.69671000
C	4.22241400	-0.25847400	0.02859700
H	4.40453100	-0.05196900	-1.04125500
C	3.24038300	-1.41613600	0.15868300
H	3.05814000	-1.58065300	1.22913700
C	1.93800900	-1.04327300	-0.52041400
H	2.12265000	-0.90876400	-1.59466500
C	1.42499900	0.27388700	0.05090900
H	1.13462100	0.12236400	1.09673600
C	2.51302100	1.34945900	-0.01540200

H	2.71981300	1.58360500	-1.06989500
C	2.14078700	2.64201100	0.69912700
H	1.65597800	2.41853900	1.65580700
H	3.07383300	3.17439100	0.89744700
O	3.84631100	-2.54801800	-0.42458100
H	3.20444500	-3.26312200	-0.42455900
O	1.03644200	-2.11645900	-0.32244700
H	0.19211600	-1.88369600	-0.73675900
O	3.67931100	0.88452900	0.63200800
O	1.28278200	3.42721400	-0.12371300
H	1.34540700	4.34083900	0.16054800
O	5.41577100	-0.50921500	0.68635800
H	-3.88576600	3.07508200	-0.09318500
H	-5.55626300	0.15039500	-0.10775000
H	5.62817100	-1.43809700	0.55261700
O	-0.50585300	-1.67598300	2.19600200
H	0.07919800	-2.06155000	1.53244200

TS F5(1-6), G=-861822.5379 kcal/mol, i512.3143 cm-1
C -0.7425080291 0.5341750888 -0.0727249255
H -0.7691691341 0.096700869 0.9231747685
C -1.8097529343 1.4756853542 -0.4992751553
H -1.7544359403 1.6152072957 -1.5865533078
C -3.1887986736 0.9563659983 -0.1128291553
H -3.1781852576 0.6908192613 0.9530410275
C -3.6173378326 -0.2710583003 -0.9315464271
H -3.653840076 0.037716686 -1.9856081295
C -2.6059172701 -1.3740022065 -0.7804169957
H -2.381954242 -1.4471045258 2.2777298568
C -2.9492228605 -2.6340769371 -0.0363567176
H -3.6213160378 -3.2570399962 -0.6373637969
H -2.0282106831 -3.1877940277 0.1449094158
O -1.6505505947 2.7328873842 0.1542690399
H -0.7131819424 2.9744701554 0.1171727056
O -4.1818116997 1.9327039569 -0.3567458182
O -4.8962514839 -0.713977702 -0.5115380816
O -1.4299827404 -1.0878900695 -1.1170711841
O -3.5468837547 -2.3780511845 1.2358742645
H -4.3542915786 -1.8774242616 1.0561178725
O 0.4450828158 0.8287622934 -0.5990610438
C 4.3754764607 -0.2071384166 -0.2531139243
H 4.4724562951 0.1033746329 -1.3083768
C 3.4063765563 -1.3778117418 -0.1531225616
H 3.3083137856 -1.6385706677 0.909241208
C 2.048151164 -0.9652205887 -0.6865544214

H 2.1439158015 -0.7328897856 -1.757016805
C 1.5964947944 0.2962162599 0.0371324058
H 1.3635314199 0.0481238007 1.0786416428
C 2.6700083068 1.3852664877 0.0001657636
H 2.7898777076 1.7285187825 -1.0376312579
C 2.3466800948 2.5867399414 0.8794719596
H 2.0595155269 2.2420726746 1.879500832
H 3.2657572817 3.1719928415 0.9679462873
O 3.9603015377 -2.4517828617 -0.8790232045
H 3.2959084846 -3.1454566877 -0.9250982717
O 1.177291916 -2.0518600454 -0.4915092869
H 0.2887336802 -1.834943661 -0.819820187
O 3.8848556378 0.8712174403 0.5012896746
O 1.3082013228 3.3671240566 0.2980660648
H 1.3479376646 4.2450139498 0.6808482057
O 5.6177133678 -0.5111415751 0.2808682608
H -3.8633501845 2.7631850147 0.0109314123
H -5.4592075661 0.0652464804 -0.4569305666
H 5.8146507414 -1.4236028578 0.0463634001
O -1.7206197984 -0.8465123202 2.6663084849
H -1.4044860491 -1.2818502856 3.4588194716

F5(1-6), G=-861837.0514 kcal/mol, 35.5377 cm-1
C -0.9168576128 0.7334096824 -0.152426752
H -0.9619739421 0.5243944164 0.9048864533
C -1.9298929396 1.6080522506 -0.7688962492
H -1.8178042931 1.6012426405 -1.858944574
C -3.2840216751 1.043704875 -0.3688352621
H -3.3018181674 0.995246622 0.7286171896
C -3.4931341338 -0.3645967849 -0.9149720608
H -3.7050331369 -0.2975112233 -1.9861629518
C -2.2634877019 -1.2740251136 -0.7765379287
H -1.4543352598 -0.2770811704 2.7604775781
C -2.0450942369 -2.0082100685 0.5426514964
H -2.492206562 -3.0026267178 0.4219985905
H -0.9734759777 -2.1450012216 0.687287884
O -1.8723538559 2.9568531143 -0.3014483154
H -1.0272432143 3.0905866283 0.1458559541
O -4.3647972542 1.8224598102 -0.8282492742
O -4.5653594096 -1.0070723486 -0.2325674533
O -1.6198559427 -1.5951214978 -1.7593594725
O -2.5671455876 -1.3618113727 1.6875984495
H -3.515604933 -1.2497816969 1.5438785367
O 0.2732968976 0.6613226355 -0.7466275424
C 4.1611338651 -0.2459191928 -0.2232072312

H 4.2368958181 0.3177680187 -1.1699032909
C 3.2747049791 -1.4671994921 -0.4175512483
H 3.1626854402 -1.9545359332 0.5602485308
C 1.9196492676 -1.0162035848 -0.9184036844
H 2.0538464278 -0.5754950084 -1.9156834543
C 1.334797532 0.0562492407 -0.007050555
H 0.9370227897 -0.4226379185 0.894018205
C 2.3504382886 1.1418213783 0.3809747059
H 2.4829857953 1.7997466334 -0.4899566759
C 1.9438635934 1.9583520542 1.6138453414
H 2.0701256089 1.3087611885 2.4848413985
H 2.6428381558 2.7907869091 1.7147051591
O 3.913350312 -2.3283378269 -1.3329495105
H 3.2938715609 -3.0291637344 -1.5559522667
O 1.0956531536 -2.156452434 -0.990007932
H 0.2583566251 -1.9498041913 -1.4372468719
O 3.5853591703 0.5691188183 0.7599527263
O 0.6293511683 2.4786409587 1.5697217446
H 0.0742995305 1.9223943483 2.1315950438
O 5.4216765292 -0.5834957322 0.2444935194
H -4.198328152 2.7320126011 -0.5605614033
H -5.3368969894 -0.4374331303 -0.3102551033
H 5.6755330342 -1.4069202876 -0.1843786205
O -0.766378413 0.3331911055 3.0782002114
H -0.6703311526 0.1713957526 4.0177939659

TS F5(4-3), G=-861806.2510 kcal/mol, -455.9585 cm-1
C -1.0128815216 0.4587881335 -0.3535855453
H -1.0633505266 0.2628577799 0.7220766773
C -1.9107902244 1.6393038813 -0.72919331
H -1.8673247438 1.7266241332 -1.8288923612
C -3.326876026 1.4049840902 -0.308531718
H -3.5122878208 1.1953313363 0.7406923061
C -3.7077269145 -0.6656042824 -1.1402389606
H -3.6695836423 -0.1488050576 -2.093642981
C -2.5963956343 -1.2680742382 -0.6422191839
H -1.0962769866 -0.9833778215 2.6169455056
C -2.5872958074 -2.2316752673 0.4884340184
H -3.4236045733 -2.9283245024 0.3953841011
H -1.6551167518 -2.7969879747 0.4714375506
O -1.4553222529 2.8263186766 -0.1204499438
H -0.4825481926 2.8262934507 -0.0895349815
O -4.292234565 2.21481524 -0.8369350235
O -4.9352199192 -1.042079924 -0.6873805386
O -1.3928852269 -0.7325197706 -1.067265385

O -2.6321981694 -1.6165105323 1.7934344201
H -3.4817783263 -1.179111431 1.8874157837
O 0.2664650602 0.7796003015 -0.7420653704
C 4.1445254479 -0.3475560873 -0.0259623401
H 4.4023659067 0.2415063128 -0.9240406448
C 3.2153931929 -1.488033636 -0.4119471678
H 2.9557121638 -2.0306404911 0.506618854
C 1.9472191232 -0.9320090883 -1.0307046919
H 2.207222359 -0.4204736462 -1.9692858422
C 1.329552798 0.0907968776 -0.0860497771
H 0.9493598868 -0.4150597402 0.8077333214
C 2.3694127569 1.1323961828 0.3417630357
H 2.663965764 1.7264726968 -0.5360816851
C 1.8556430077 2.09027482 1.4072777624
H 1.1688260725 1.5730071653 2.0857808398
H 2.7226819233 2.4413399756 1.9731669857
O 3.9180460365 -2.3292571933 -1.3018873653
H 3.2976750284 -2.9825344298 -1.6371092252
O 1.104522736 -2.0306229414 -1.2950532436
H 0.2080947571 -1.7015058042 -1.4547565462
O 3.4953221104 0.4831118643 0.8970409876
O 1.2066423006 3.1833799134 0.7632925063
H 0.9220006204 3.8035943775 1.4375250469
O 5.2942921796 -0.8014315312 0.6044633052
H -3.9175088389 2.7565609957 -1.5370939166
H -5.602306722 -0.4692461601 -1.0706250343
H 5.5607502433 -1.612579172 0.1604359839
O -0.2574459213 -0.5530189019 2.8505415816
H 0.1392328329 -1.0979305803 3.531666209

F5(4-3), G= -861818.0694 kcal/mol, 28.8649 cm-1
C -1.0448904321 0.5652442665 -0.363233624
H -1.0843588256 0.2960325913 0.6921206539
C -1.8787414089 1.8218509207 -0.6107528451
H -1.7537994442 2.0850931743 -1.6741283521
C -3.3354570827 1.6155441516 -0.2817347551
H -3.9786761892 1.0754628848 -0.9649164131
C -3.4396051062 -1.718873953 -1.5293657106
H -3.4189278031 -1.3575941321 -2.5523308854
C -2.5091403469 -1.3292994186 -0.662520183
H -1.3951765413 -0.3374241747 2.7388108769
C -2.4439433268 -1.8015339983 0.753577581
H -3.0578042321 -2.6986846068 0.8460599276
H -1.4098946759 -2.0602475682 1.0022205681
O -1.4277711023 2.8647955611 0.2148355658

H -0.4534677527 2.8809284009 0.2556844077
O -3.9585180535 2.6186951243 0.3712633967
O -4.4316947375 -2.5816199455 -1.1822556241
O -1.5014918005 -0.5244772053 -1.1684658273
O -2.8831859648 -0.8386025001 1.7196105331
H -3.3573711622 -0.1290729886 1.2612828432
O 0.251163548 0.8507424042 -0.7403207891
C 4.0677743314 -0.4852065184 -0.0577568417
H 4.3530916699 0.1004626216 -0.9497477963
C 3.0790235045 -1.5700917864 -0.4585675103
H 2.7928683125 -2.1116034628 0.4528418148
C 1.8445752532 -0.9331083377 -1.0648648259
H 2.1323836183 -0.4176864588 -1.992805073
C 1.2813620234 0.1030504699 -0.1008784074
H 0.8814949316 -0.3996313662 0.7871654551
C 2.3731705006 1.0829604434 0.341157217
H 2.6917453515 1.6778041528 -0.5275886159
C 1.9207322782 2.0399682954 1.4350769818
H 1.25434672 1.5299276344 2.1381673656
H 2.8189097158 2.3714079917 1.9618246746
O 3.7344881665 -2.4326772682 -1.3631385286
H 3.081036938 -3.0486579385 -1.7058537981
O 0.9376441717 -1.9741202057 -1.3499538673
H 0.0823722615 -1.5795635527 -1.5710620952
O 3.4668229747 0.3662831174 0.8788591898
O 1.260594065 3.1567629137 0.837633044
H 1.1718090873 3.8419144787 1.5030119888
O 5.1936611681 -1.0078482086 0.5613428603
H -3.260079051 3.1699564359 0.757110031
H -4.945509579 -2.8108887457 -1.9566321388
H 5.4126900413 -1.82885821 0.1095688603
O -0.466856099 -0.1383004049 2.9495783687
H -0.2769459152 -0.5958050787 3.7697153016
